# Proceedings of the International Cancer Imaging Society (ICIS) 17th Annual Teaching Course: Berlin, Germany. 02 - 04 October 2017

**DOI:** 10.1186/s40644-017-0126-4

**Published:** 2017-09-07

## Monday 2^nd^ October – Morning Session

### 09:00 - 10:30 Structured Reporting and Decision Support

#### O1 PI-RADS: update 2017

This abstract is not included here as it has already been published [1, 2].

**References**

[1] Jelle O. Barentsz , Jeffrey C. Weinreb, Sadhna Verma , Harriet C. Thoeny d, Clare M. et al. Synopsis of the PI-RADS v2 Guidelines for Multiparametric Prostate Magnetic Resonance Imaging and Recommendations for Use. European Urology 2016 (69):41-49.

[2] Jeffrey C. Weinre, Jelle O. Barentsz, Peter L. Choyke , Francois Cornud, Masoom A. Haider , Katarzyna J. Macura, et al. PI-RADS Prostate Imaging – Reporting and Data System: 2015, Version 2. European Urology 2016(69): 16-40

#### O2 LI-RADS: update 2017

##### Jay P. Heiken (heikenj@wustl.edu)

###### Mallinckrodt Institute of Radiology, Washington University School of Medicine, St. Louis, MO 63110, USA

LI-RADS (Liver Imaging Reporting and Data System) is a comprehensive system for standardise interpretation and reporting of CT, MR and US examinations performed on patients at risk for hepatocellular carcinoma (HCC). LI-RADS was developed by a large committee with international and multidisciplinary input and is supported by the American College of Radiology (ACR). The aims of LI-RADS are to:

• Establish minimum technical parameters for CT, MR, and US HCC surveillance

• Standardise: terminology, interpretation, reporting and imaging management

• Enhance communication among radiologists, hepatologists, surgeons and pathologists

LI-RADS (version2017) was released at the end of June 2017. Different from the previous versions, which were PowerPoint based, the new version is a downloadable pdf. New content in v.2017 includes modules on ultrasound surveillance, contrast enhanced ultrasound (CEUS), reporting, management and tumour response. In addition, the CT/MR algorithm has been modified. The details of these changes will be discussed.

**References**

1. https://www.acr.org/quality-safety/resources/LIRADS

### 09:00 – 10:30 Head and Neck

#### O3 MRI in nasopharyngeal carcinoma

##### Ann D King (King2015@cuhk.edu.hk)

###### Department of imaging and interventional radiology, Chinese University of Hong Kong, Hong Kong

**Abstract**

Nasopharyngeal carcinoma (NPC) is most commonly an undifferentiated carcinoma with a strong association with the Epstein Barr virus (EBV) and familial tendency. MRI is the modality of choice for NPC staging of the primary tumour and nodal metastases. The primary tumour spreads to the nasal cavity and infrequently to the oropharynx (T1); parapharyngeal space, medial and lateral pterygoid, and prevertebral muscles (T2); skull base, cervical vertebrae, pterygoid structures and paranasal sinuses (T3); intracranial structures, cranial nerves, orbit, parotid gland, beyond the lateral margin of the lateral pterygoid muscle, and hypopharynx (T4). NPC has a propensity to spread to nodes, the first echelon being nodes in the retropharyngeal region or upper internal jugular chain from where nodes spread in orderly fashion down the neck. Nodal staging differs from that squamous cell carcinoma in that bilateral nodes are down staged to NI when they are retropharyngeal, 6cm is the only criterion for size, and lower neck nodes are designated as N3. The first part of the lecture will focus on MRI staging of primary tumour and nodal metastases using a structured approach and report. The lecture will illustrate the staging process including assessment of the complex anatomy at the skull base, highlight areas important for NPC management, and review the changes in the latest 8^th^ edition of the UICC/AJJC classification.

More recently MRI is also being used for the early detection of NPC. MRI has a high diagnostic accuracy of NPC detection, 10% of MRI detected NPCs are endoscopically occult, and MRI identifies slow growing tumours up to three years before they can be found endoscopically. In the second part of the lecture this new role of MRI for early detection of NPC in symptomatic patients and subjects in EBV population screening studies will be discussed.

**References**

1. Chong VF et al. Nasopharyngeal carcinoma with intracranial spread: CT and MR characteristics. JCAT. 1996;20:563-539

2. King AD et al. MRI of local disease in nasopharyngeal carcinoma: tumour extent vs tumour stage. Br J Radiol. 1999;72:734-741

3. Chong VF et al. Nasopharyngeal carcinoma. Eur J Radiol. 2008;66:437-447.

4. King AD, Bhatia KS. Magnetic resonance imaging staging of nasopharyngeal carcinoma in the head and neck. World Journal of Radiology. 2010;2:159-165

5. Ho FC et al. Patterns of regional lymph node metastasis of nasopharyngeal carcinoma: a meta-analysis of clinical evidence. BMC Cancer. 2012;12:98

6. King AD et al. Primary nasopharyngeal carcinoma: diagnostic accuracy of MR imaging versus that of endoscopy and endoscopic biopsy. Radiology. 2011;258:531-7

7. King AD et al. Detection of nasopharyngeal carcinoma by MR imaging: Diagnostic Accuracy of MRI compared with endoscopy and endoscopic biopsy based on long-term follow-up. AJNR 2015;36:2380-2385

#### O4 Perineural tumour spread in head and neck cancer

##### Robert Hermans (robert.hermans@uzleuven.be)

###### Department of Radiology, University Hospitals Leuven, Leuven, Belgium

Many different malignant neoplasms may be seen in the head and neck, one of the most common arises from the mucosal surfaces, being squamous cell carcinoma. These cancers spread along the mucosal surface, but also submucosally, typically preferring the paths of least resistance. As there are many nerves present in the head and neck region, these structures may provide tumours the opportunity to spread over a considerable distance from their point of origin.

Perineural tumour spread occurs in all head and neck malignancies; adenoid cystic carcinoma, a tumour of salivary gland origin, is notorious for its tendency to spread along nerves. Perineural tumour spread is associated with a decreased survival rate. Symptoms include pain, paraesthesias and muscle weakness, but about 40% of patients do not show particular symptoms. Imaging diagnosis is important to map the full tumour extent, and to avoid tumour progression from unrecognised perineural spread.

Perineural tumour spread occurs most frequently along the maxillary nerve, mandibular nerve and facial nerve. Imaging findings in perineural tumour spread include thickening and/or enhancement of one or more nerve branches; widening, destruction or enhancement of a skull base neural foramen or canal (e.g. foramen ovale, vidian canal); small tumoural lesions at some distance from the primary site, in a neural 'crossroad' such as the pterygopalatine fossa or Meckel's cave; denervation atrophy of muscles supplied by the affected nerve.

#### O5 Molecular imaging in head and neck cancer

##### Wim J.G. Oyen (Wim.Oyen@icr.ac.uk)

###### The Institute of Cancer Research / Royal Marsden Hospital, London, UK

Next to MRI and CT, in recent years, the role of molecular imaging with FDG-PET/CT has significantly increased in staging, monitoring the response to therapy and detection of relapse.

FDG-PET/CT plays an important role at diagnosis in patients with metastatic lymph nodes in the neck from an unknown primary tumour, guiding the choice of biopsy sites at subsequent endoscopy. Furthermore, FDG-PET/CT provides pivotal additional information in patients with equivocal or contradictory findings on other diagnostic modalities to provide clinical decision making on radical or palliative treatment. This is particularly important in patients who have a high risk of metastatic disease such as advanced loco-regional disease and primary sites which are more likely to result in distant metastases such as nasopharyngeal cancer. [1]

Although not yet widely adopted as the standard of care, the role of FDG-PET/CT in radiation treatment planning, monitoring response to (chemo) radiotherapy and adaptation of treatment based on early interim FDG-PET/CT (iPET) scanning is expanding. In a recent systematic review, it was observed that most studies confirm the value of iPET to predict response to chemoradiotherapy. [2] A few studies did highlight that predictive value of iPET was lower as compared to FDG-PET/CT acquired 2 to 4 months after completion of chemoradiotherapy, which correlated well with local and regional control and survival. [2]. The best timing for iPET to assess response during radiation therapy remains to be established, although 1-2 weeks seems the best choice as it leaves the opportunity to adapt the treatment regimen.

It is accepted that in patients with advanced nodal disease (stage N2 or N3) and who have received primary chemoradiotherapy, PET-CT-guided surveillance after completion of chemoradiotherapy results in similar survival with considerably fewer operations as compared to patients who underwent routine neck dissection. [3] This PET/CT-based strategy proved to be high cost-effective. [4] In patients suspected of recurrent laryngeal carcinoma after radiotherapy, FDG-PET/CT is also effective a first-line diagnostic investigation, prior to performing a direct laryngoscopy with biopsy under general anaesthesia, reducing the need for direct laryngoscopy by more than 50% without jeopardizing quality of treatment. [5]

In conclusion, FDG-PET/CT plays an increasingly important role in the assessment of disease activity in patients with head and neck cancer in all stages of the patients’ journey.

**References**

1. **Xi** K, Xie X, Xi S : **Meta-analysis of (18) fluorodeoxyglucose positron emission tomography-CT for diagnosis of lung malignancies in patients with head and neck squamous cell carcinomas.** *Head Neck* 2015, **37**: 1680-1684.

2. Garibaldi C, Ronchi S, Cremonesi M, et al. **Interim 18F-FDG PET/CT During Chemoradiation Therapy in the Management of Head and Neck Cancer Patients: A Systematic Review.** *Int J Radiat Oncol Biol Phys* 2017, **98**: 555-573.

3. Mehanna H, Wong WL, McConkey CC, et al. **PET-CT Surveillance versus Neck Dissection in Advanced Head and Neck Cancer.** *N Engl J Med* 2016, **374**: 1444-1454.

4. Mehanna H, McConkey CC, Rahman JK, et al. : **PET-NECK: a multicentre randomised Phase III non-inferiority trial comparing a positron emission tomography-computerised tomography-guided watch-and-wait policy with planned neck dissection in the management of locally advanced (N2/N3) nodal metastases in patients with squamous cell head and neck cancer.** *Health Technol Assess* 2017, **21**: 17.

5. de Bree R, van der Putten L, van Tinteren H, et al : **Effectiveness of an (18)F-FDG-PET based strategy to optimise the diagnostic trajectory of suspected recurrent laryngeal carcinoma after radiotherapy: The RELAPS multicenter randomised trial.** *Radiother Oncol* 2016**, 118**: 251-256.

### 11:00 - 12:30 Precision medicine

#### O6 Precision medicine in oncology – concepts and promises

##### Aristoteles Giagounidis (aristoteles.giagounidis@vkkd-kliniken.de)

###### Clinic for Oncology, Hematology and Palliative Care, Marien Hospital, Düsseldorf, 40479, Germany

Precision medicine, the promise of targeting diseases by specifically aiming at special properties of tumour cells, has received major attention in the recent past. A huge number of drugs are underway to improve patient outcomes and several recent drug developments have already significantly changed the fate of oncology patients. Different classes of drugs need to be reviewed to allow an understanding of therapeutic interventions.

The oldest targeted drug treatments available are monoclonal antibodies. These antibodies all target structures on the cell surface and are being used in hematology, solid oncology, as well as in rheumatology and neurology. Binding of the antibody to its target structure is specific and leads to complement-induced cell lysis, recognition by the immune system or direct, antibody-dependent cell lysis. One level below, within the cell cytoplasm, the tyrosine kinases that are members of the signal transduction chain, can also be targeted. This disruption can lead to long lasting responses in patients who failed several other lines of therapy. In some instances, tyrosine kinase inhibitors have led to cure in hitherto incurable diseases. Unravelling the immune system to enable it to recognise tumour cells has led to the invention of PD-1 and PD-L1 inhibitors. These were first used in malignant melanoma but have now expanded to a wide variety of solid tumours and have led to impressive results especially in cells with high mutation rate, as these tend to display a lot of PD-L1 proteins on their surface. The most exciting news, however, comes from chimeric antigen receptor T (CAR-T)- cells, one of which has recently been approved for the treatment of juvenile acute leukemia. Here, cytotoxic T-cells from a patient are isolated and genetically modified by implanting into the cell surface a recognition molecule for a tumour antigen. Because T-cells can replicate in vivo, these cells not only recognise and destroy a target cell but will prevail as a long-term memory within the body of the recipient. This development is poised to transform oncology as we know it and may become the most important advance in the treatment of tumours for years to come.

#### O7 Tumour heterogeneity: what does it mean?

##### Dow-Mu Koh (Mu.Koh@icr.ac.uk)

###### Royal Marsden Hospital, Sutton, Surrey, SM2 5PT, UK

Cancers arise from unregulated cellular growth and exhibits a range of pathophysiological characteristics and behaviour. The term “tumour heterogeneity” refers to differences in tumour features that have arisen as a result of mechanistic processes including stem-cell origin, tumour evolution (linked to genetic mutations and adverse tumour microenvironment) and clonal resistance (as a consequence of treatment). Tumour heterogeneity may be inter-cellular, intra-tumoural (i.e. within the same tumour), inter-tumoural (i.e. between lesions in the same patient) and inter-individual (i.e. between patients).

The understanding of tumour heterogeneity is important because it is a barrier cancer cures. Tumour heterogeneity is linked to treatment resistance and the emergence of aggressive tumour phenotypes. The ability to identify, describe and communicate heterogeneity of tumours on imaging may provide a basis for adaptive treatments to improve patient outcomes.

Current approaches used to enhance the understanding of tumour heterogeneity include genomics/ molecular analysis of multi-tumoural region tissue sampling and/or longitudinal tumour tissue sampling. However, non-invasive imaging using CT, MRI and PET enables the characteristics of multiple disease sites to be evaluated in a single study, thereby providing information about intra- and inter-tumour heterogeneity.

Using functional and molecular imaging techniques, imaging can be used to quantitatively measure different aspects of tumour biology; thus identifying imaging phenotypes linked to different stem-cell origins, gene expression, tumour evolution, treatment efficacy (including differential treatment response) and the emergence of clonal resistance.

The emerging evidence for the use of imaging to explore and understand tumour heterogeneity will be presented and discussed.

### 11.00 - 12.30 Joint with DEGRO: Delineation of Tumour and Normal Tissue

#### O8 Image interpretation in the post treatment pelvis

##### Andrea Rockall (a.rockall@imperial.ac.uk)

###### Department of Radiology, The Royal Marsden NHS Foundation Trust, Fulham Rd, London SW3 6JJ, UK

Follow-up imaging is a significant part of the daily routine practice in cancer imaging. The work-load has increased due to improved outcomes with longer patient survival rates, as well as due to the development of the increasing range of salvage therapy options in the case of disease relapse. It is important that the radiologist is familiar with the post therapy appearances of the pelvis in order to recognise the typical changes following surgery or radiotherapy. In addition, the radiologist needs to recognise complications of treatment and disease relapse.

**Post surgical pelvis**

The typical appearance of the pelvis following gynaecologic cancer surgery depends on the procedure. The most common procedure includes hysterectomy and bilateral salpingo-oopherectomy and in many cases, the pelvic side-wall lymph nodes may be sampled or removed. The vaginal vault and pelvic side-wall nodes should be carefully inspected as these are frequent sites of pelvic recurrence (1). The use of imaging for follow-up surveillance is variable (2). However, follow-up imaging in the case of fertility-preserving surgery requires special attention (3). Regular surveillance imaging is typically performed in order to detect any early signs of relapse following procedures including trachelectomy for early stage cervix cancer, unilateral salpingo-oopherectomy for borderline ovarian neoplasm or conservative management of endometrial cancer.

Post-operative lymphocysts along the pelvic side-walls may be seen following nodal dissection and these usually decrease over time and are rarely of clinical significance; occasionally a lymphocyst may become infected. Significant post-operative complications, such as abscess or haemorrhage, are usually clinically recognised and imaging is used to clarify the extent and direct further management, such as percutaneous drainage.

**Post radiotherapy pelvis**

Imaging is regularly used to evaluate response to treatment in cases of non-surgical management, such as radiotherapy and/or chemotherapy. The evaluation of response to radiotherapy is highly important and will be covered in as a separate topic. Following completion of radiotherapy, there are several recognizable appearances that are frequently seen and are not usually of clinical concern (4). These include thickening and oedema of the bladder and rectum and thickening of the small bowel loops. Chronic signs of radiotherapy toxicity should also be recognised and reported, including cervical stenosis, bowel stricture, insufficiency fractures in the pelvis and the development of fistula, all of which may be associated with significant symptoms.

**Relapse and salvage therapy**

It is essential to know the typical sites of pelvic relapse and the appearances on imaging in order to ensure early detection. The success of salvage treatment is best when there is limited central pelvic recurrent disease amenable to exenterative surgery or focussed salvage radiotherapy. FDG-PET/CT is used to determine whether relapse is restricted to a single pelvic site or whether this is more widespread (5) and may also be helpful, in combination with MRI, in providing a surgical road-map (6). In the case of planning exenterative surgery or stereotactic radiotherapy, accurate description of the anatomical relationships between disease and pelvic structures is very important. This information can not only assist the surgeon but also help to inform the patient as to the likely treatment outcomes.

**References**

1. Sohaib SA, Houghton SL, Meroni R, Rockall AG, Blake P, Reznek RH. Recurrent endometrial cancer: patterns of recurrent disease and assessment of prognosis. *Clinical Radiology* 2007;62(1):28-34; discussion 35-36.

2. Nobbenhuis MA, Balasubramani L, Kolomainen DF, Barton DP. Surgical management and follow-up of patients with cervical cancer: survey of gynaecological oncologists in the UK. *J Obstet Gynaecol*. 2012 Aug;32(6):576-9.

3. Rockall AG, Qureshi M, Papadopoulou I, Saso S, Butterfield N, Thomassin-Naggara I, Farthing A, Smith JR, Bharwani N. Role of Imaging in Fertility-sparing Treatment of Gynecologic Malignancies. *Radiographics*. 2016 Nov-Dec;36(7):2214-2233.

4. Papadopoulou I, Stewart V, Barwick TD, Park WE, Soneji N, Rockall AG, Bharwani N. Post-Radiation Therapy Imaging Appearances in Cervical Carcinoma. *Radiographics*. 2016 Mar-Apr;36(2):538-53.

5. Brar H, May T, Tau N, Langer D, MacCrostie P, Han K, Metser U. Detection of extra-regional tumour recurrence with (18)F-FDG-PET/CT in patients with recurrent gynaecological malignancies being considered for radical salvage surgery. *Clinical Radiology*. 2017 Apr;72(4):302-306.

6. Vargas HA, Burger IA, Donati OF, Andikyan V, Lakhman Y, Goldman DA, Schöder H, Chi DS, Sala E, Hricak H. Magnetic resonance imaging/positron emission tomography provides a roadmap for surgical planning and serves as a predictive biomarker in patients with recurrent gynecological cancers undergoing pelvic exenteration. *Int J Gynecol Cancer*. 2013 Oct;23(8):1512-9.

## Monday 2^nd^ October – Afternoon Session

### 14:00 - 14:30 Keynote Lecture 1

#### O9 Cancer imaging: leveraging the strengths of precision imaging for precision medicine

##### Annick D. Van den Abbeele (abbeele@dfci.harvard.edu)

###### Chair, Department of Imaging, Dana-Farber Cancer Institute, Boston, MA 02215, USA; Founding Director, Center for Biomedical Imaging in Oncology, Dana-Farber Cancer Institute, Boston, MA 02215, USA

Co-Director, Tumour Imaging Metrics Core, Dana-Farber/Harvard Cancer Center, Boston, MA 02215, USA; Harvard Medical School, Boston, MA, 02115, USA; Co-Editor-In-Chief, Cancer Imaging, International Cancer Imaging Society

Precision medicine and precision oncology aim to deliver the right treatment to the right patient at the right dose through the right route of administration. The precision medicine initiative has led to major paradigm shifts in precision oncology, first with the implementation of molecularly-targeted therapies [1, 2], then with immune therapy [3, 4]. We are now at a point where we can deliver cancer treatment with great precision, and the prevention, diagnosis and treatment of cancer are becoming proactive disciplines using evidence-based individualised medicine to meet the goals of precision medicine/oncology.

These advances have not come without challenges for the cancer imaging community. We have had to readjust our evaluation of therapeutic response from traditional anatomic-based criteria based on tumour shrinkage to a new context where knowledge of novel therapies and their biologic effects are paramount as to not misinterpret response. Molecularly targeted therapies may, indeed, be effective despite the absence of tumour shrinkage, while the appearance of new lesions or an increase in tumour size following immune therapy may represent pseudoprogression rather than progressive disease [5, 6].

Cancer Imaging is, however, uniquely placed to validate and deliver *precision imaging* to the precision medicine/oncology initiative. Advances in the functional capabilities of various imaging modalities including CT, MRI, ultrasound, nuclear medicine and PET and hybrid technologies (SPECT/CT, PET/CT and PET/MRI) are providing anatomic and functional whole body imaging noninvasively in one setting that can define the entire tumour burden, characterise tumour heterogeneity, guide biopsy to the relevant site, evaluate the tumour and its microenvironment simultaneously, help enrich patient populations, add prognostic value, and be clinically actionable. Molecular imaging, in particular, has the potential of exploring all the hallmarks of cancer biology, providing specificity that is relevant to the biology of the tumour and its microenvironment, and guiding pre-clinical and clinical trial design with timely and effective translation of cancer discovery. Molecular imaging can also detect primary and secondary resistance, provide tumour response assessment early after initiation of therapy (which may add predictive value), offer pharmacokinetic and pharmacodynamic information, help define biologic effectiveness prior to MTD, and assess and monitor toxicity. These capabilities may result in shorter trials and improve the timeline for drug or imaging probe development.

Achieving these goals requires standardization of imaging protocols and consistent, accurate and reproducible longitudinal tumour assessments in oncologic clinical trials and clinical practice [7]. It also requires a collaborative approach between basic and translational scientists, imagers, academia and industry. A “wall-less” disease-centric infrastructure structured as a multitalented and multidisciplinary team focused on facilitating bi-directional exchanges and communication can help achieve this goal [8].

Achieving precision cancer imaging will also require changes in the training of cancer imaging specialists at the residency and fellowship levels to produce cancer imaging specialists who are literate in more than one specialty and imaging modality. The training of the next generation of “Precision Imagers” needs a new disease-based curriculum that teaches the language of oncology, navigates through the various “-omics” and highlights the biology behind cancer and drug development [9, 10].

**References**

1. Demetri GD, von Mehren M, Blanke CD, Van den Abbeele AD, Eisenberg B, Roberts P, Heinrich MC, Tuveson DA, Singer S, Janicek M, Fletcher JA, Silverman S, Harmon D, Silberman S, Capdeville R, Kiese B, Peng B, Dimitrijevic S, Druker BJ, Corless C, Fletcher CDM, Joensuu H. **Efficacy and Safety of Imatinib Mesylate in Advanced Gastrointestinal Stromal Tumours.** *N Engl J Med* 2002, **347**:472-480.

2. Van den Abbeele AD, Badawi RD. **Use of positron emission tomography in oncology and its potential role to assess response to imatinib mesylate therapy in gastrointestinal stromal tumours (GISTs)**. *Eur J Cancer* 2002,**38**(Suppl 5):S60–5.

3. Hodi, F. S. *et al*. **Improved survival with ipilimumab in patients with metastatic melanoma.** *N. Engl. J. Med.* 2010, **363**, 711–723.

4. Topalian, S. L. *et al*. **Safety, activity, and immune correlates of anti-PD-1 antibody in cancer.** *N. Engl. J. Med.* 2012, **366**, 2443–2454.

5. Van den Abbeele AD. **The Lessons of GIST--PET and PET/CT: A new paradigm for imaging.** *Oncologist* 2008, **13** Suppl 2:8-13.

6. Wolchok JD, et al. **Guidelines for the evaluation of immune therapy activity in solid tumours: immune-related response criteria**. *Clin Cancer Res*. 2009, **15**(23):7412–20.

7. Urban T, Ziegler E, Lewis R, Hafey C, Sadow C, Van den Abbeele AD*, Harris GJ* [* Co-senior authors]. **LesionTracker: Extensible Open-Source Zero-Footprint Web Viewer for Cancer Imaging Research**. *Cancer Research*. In press.

8. Van den Abbeele AD, Krajewski KM, Tirumani SH, Fennessy, DiPiro PJ, Nguyen QD, Harris GJ, Jacene HA, Lefever G, Ramaiya NH. **Cancer Imaging at the Crossroads of Precision Medicine: Perspective from an Academic Imaging Department in a Comprehensive Cancer Center**. *J Am Coll Radiol.* 2016 Apr;**13**(4):365-71.

9. Howard SA, Krajewski KM, Weissman BN, Ramaiya NH, Van den Abbeele AD. **Cancer imaging training in the 21st century, an overview of where we are, and where we need to be.** *J Am Coll Radiol*. 2015 Jul;**12**(7):714-20.

10. Krajewski KM, Howard SA, Ramaiya NH, Shinagare AB, Van den Abbeele AD, Fennessy FM. **Cancer Imaging Fellowship Training: Utility and Added Value in the Modern Era**. *J Am Coll Radiol.* In press.

### 14:30 - 15:30 Prostate: When to Treat

#### O10 Early diagnosis of significant cancer – how good is mpMRI?

This abstract is not included here as it has already been published [1].

**Reference**

[1] Jelle Barentsz a,*, Jurgen J. Futterer a, Anwar R. Padhani. Will Magnetic Resonance Imaging-guided Biopsy Replace Systematic Biopsy? European Urology Focus 2015 (1): 152-155.

#### O11 MRI in active surveillance of prostate cancer

##### Jurgen J. Fütterer (Jurgen.Futterer@radboudumc.nl)

###### Department of Radiology and Nuclear Medicine, Radboudumc, Nijmegen, The Netherlands

At present, treatment choice for prostate cancer patients at low or intermediate risk of disease progression lies between active surveillance and radical therapies, such as radical prostatectomy or radiotherapy. For these patients, radical treatments have a comparable effectiveness, with a risk of specific death of less than 1% in 15 years. However, none is divided of consequences on the quality of life and can induce significant morbidities such as incontinence and impotence (1-3). On the other hand, active surveillance has established limitations. It is a source of anxiety and imposes, according to the current terms of use, clinical (rectal examination), biological (PSA) and yearly repeat biopsies reassessments that represent a burden for the patient and increase the risk of infection (4). Lastly, the presumed low risk of progression defined by systematic biopsies is underestimated in about 30% of cases, particularly when the tumour originates in the anterior part of the prostate. Thus, more than one third of men exit the protocol at mid-term and ask for a radical treatment.

The role of imaging has been increased in patients who choose for active surveillance. Multi-parametric MRI is applied to identify patients with clinically-significant cancer and larger disease burden who would most likely benefit from intervention. Thus, imaging is used as a selection tool. Multi-parametric MRI in the surveillance of low-grade and low-volume disease is rising. In case of disease progression on imaging, MRI targeted biopsy is a viable option to proof this stage migration. At present, multi-parametric MRI is not in the current international guidelines for active surveillance. However, evidence is growing in favor for the implementation of multi-parametric MRI in the active surveillance setting.

**References**

1. Korfage IJ, Essink-Bot ML, Borsboom GJ, Madalinska JB, Kirkels WJ, Habbema JD et al. Five-year follow-up of health-related quality of life after primary treatment of localised prostate cancer. Int J Cancer 2005 August 20;116(2):291-6.

2. Potosky AL, Davis WW, Hoffman RM, Stanford JL, Stephenson RA, Penson DF et al. Five-year outcomes after prostatectomy or radiotherapy for prostate cancer: the prostate cancer outcomes study. J Natl Cancer Inst 2004 September 15;96(18):1358-67.

3. Stanford JL, Feng Z, Hamilton AS, Gilliland FD, Stephenson RA, Eley JW et al. Urinary and sexual function after radical prostatectomy for clinically localised prostate cancer: the Prostate Cancer Outcomes Study. JAMA 2000 January 19;283(3):354-60.

4. Nam RK, Saskin R, Lee Y, Liu Y, Law C, Klotz LH et al. Increasing hospital admission rates for urological complications after transrectal ultrasound guided prostate biopsy. J Urol. 2010 Mar;183(3):963-8.

### 14:00 - 15:30 Interventions: Percutaneous and Intraoperative

#### O12 Image-guided thermoablation of liver tumours

##### Bernhard Gebauer (bernhard.gebauer@charite.de)

###### Charité – Universitätsmedizin Berlin, Campus Virchow-Klinikum, Augustenburger Platz 1, 13353 Berlin, Germany

Thermal ablation of tumours plays an important role in the local tumour therapy. In thermal ablation tumour cells are locally in situ heated up and destroyed. For in situ thermal destruction radiofrequency (RFA), microwave (MWA), laser (LITT) or high-focused ultrasound (HiFU) are established (1). In addition to heating thermal ablation cooling cancer cells to ice, so called cyotherapy, is another form of thermal ablation technique. On the other side also non-thermal local tumour ablations, e.g. local irradiation (SBRT, Cyberknife, brachytherapy) or irreversible electroporation (IRE) are possible (2, 3).

Thermal tumour ablations are established in primary or secondary liver and lung tumours, but are also used in kidney tumours, bone tumours, adrenal tumours, prostate cancer, breast cancer and others. The most accepted indications are radiofrequency ablation (RFA) or microwave ablation (MWA) of hepatocellular cancer (HCC) in the liver and localised renal cell cancer (RCC).

In HCCs the accepted size of a single nodule is 3 cm in diameter for thermal ablation (4, 5). In larger tumours local tumour recurrence rates after ablation are increasing because of heat sink effect. HCCs nodules are hypervascularised and this hyperperfusion is transporting the necessary heat for cancer cell ablation away. Pre-ablative embolisation like TACE is an accepted method to prevent this heat sink effect in tumours up to 4-4.5 cm in diameter (6). In comparison of MWA and RFA it is unclear which method generates a higher local control rate after thermal ablation, but it is clear that MWA is much faster and creates larger ablation zones (7-9).

In renal cell cancers RFA and cyoablation are the preferred methods of local thermal ablation. Usually localised RCCs (T1a) are an indication for local ablation and current studies did show that thermal ablation is equally oncological effective as partial nephrectomy, but with a better preservation of renal function (10-12).

Another very well accepted indication of thermal ablation is RFA of symptomatic osteoid osteomas. Osteoid osteoma is not a malignant tumour, but is very painful. Localized thermal ablation of this prostaglandin producing quickly resolves pain in these young patients (13).

**References**

1. Yu H, Burke CT. Comparison of percutaneous ablation technologies in the treatment of malignant liver tumours. Semin Intervent Radiol. 2014;31(2):129-37.

2. Narayanan G. Irreversible Electroporation. Semin Intervent Radiol. 2015;32(4):349-55.

3. Collettini F, Schreiber N, Schnapauff D, Denecke T, Wust P, Schott E, et al. CT-guided high-dose-rate brachytherapy of unresectable hepatocellular carcinoma. Strahlenther Onkol. 2015;191(5):405-12.

4. Forner A, Llovet JM, Bruix J. Hepatocellular carcinoma. Lancet. 2012;379(9822):1245-55.

5. Cho JY, Choi MS, Lee GS, Sohn W, Ahn J, Sinn DH, et al. Clinical significance and predictive factors of early massive recurrence after radiofrequency ablation in patients with a single small hepatocellular carcinoma. Clin Mol Hepatol. 2016;22(4):477-86.

6. Liao M, Huang J, Zhang T, Wu H. Transarterial chemoembolization in combination with local therapies for hepatocellular carcinoma: a meta-analysis. PloS one. 2013;8(7):e68453.

7. van Tilborg AA, Scheffer HJ, de Jong MC, Vroomen LG, Nielsen K, van Kuijk C, et al. MWA Versus RFA for Perivascular and Peribiliary CRLM: A Retrospective Patient- and Lesion-Based Analysis of Two Historical Cohorts. Cardiovasc Intervent Radiol. 2016;39(10):1438-46.

8. Thamtorawat S, Hicks RM, Yu J, Siripongsakun S, Lin WC, Raman SS, et al. Preliminary Outcome of Microwave Ablation of Hepatocellular Carcinoma: Breaking the 3-cm Barrier? J Vasc Interv Radiol. 2016;27(5):623-30.

9. Potretzke TA, Ziemlewicz TJ, Hinshaw JL, Lubner MG, Wells SA, Brace CL, et al. Microwave versus Radiofrequency Ablation Treatment for Hepatocellular Carcinoma: A Comparison of Efficacy at a Single Center. J Vasc Interv Radiol. 2016;27(5):631-8.

10. Kim HJ, Park BK, Park JJ, Kim CK. CT-Guided Radiofrequency Ablation of T1a Renal Cell Carcinoma in Korea: Mid-Term Outcomes. Korean J Radiol. 2016;17(5):763-70.

11. Cooper CJ, Teleb M, Dwivedi A, Rangel G, Sanchez LA, Laks S, et al. Comparative Outcome of Computed Tomography-guided Percutaneous Radiofrequency Ablation, Partial Nephrectomy or Radical Nephrectomy in the Treatment of Stage T1 Renal Cell Carcinoma. Rare tumours. 2015;7(1):5583.

12. Chang X, Liu T, Zhang F, Ji C, Zhao X, Wang W, et al. Radiofrequency ablation versus partial nephrectomy for clinical T1a renal-cell carcinoma: long-term clinical and oncologic outcomes based on a propensity score analysis. J Endourol. 2015;29(5):518-25.

13. Gebauer B, Collettini F, Bruger C, Schaser KD, Melcher I, Tunn PU, et al. Radiofrequency ablation of osteoid osteomas: analgesia and patient satisfaction in long-term follow-up. Rofo. 2013;184(10):959-66.

#### O13 Irreversible Electroporation of locally advanced pancreatic cancer

##### Daniel Busch, Sarah Hilswicht, Mathias Birth

###### Department of General, Visceral, Thoracic and Vascular Surgery, Helios Hanseklinikum Stralsund, Stralsund, Germany

####### **Correspondence:** Daniel Busch (Busch.Daniel2@helios-kliniken.de)

Irreversible electroporation (IRE) is a relatively new, non-thermal ablation technique mainly used in prostate cancer and liver masses. IRE utilises a series of short, high-voltage pulses conducted via needle-electrodes. The induced microporation of the cell membranes lead to irreversible defects causing cell death. Since IRE is a non-thermal ablation technique it does not have the side-effects of those methods such as the heat sink effect or thermal damage of vulnerable nearby structures. IRE can thus also be used close to blood vessels. Therefore IRE is a suitable method for treatment of locally advanced pancreatic cancer.

Only up to 20% of all patients diagnosed with pancreatic cancer are eligible for a surgical oncological resection (Whipple’s procedure, pancreatectomy or resection of the pancreatic tail) which – to date - is the only available cure [1]. Even with a complete surgical resection (R0) patients have a poor prognosis with a 5-year survival rate of 20-25% [2]. The main reason for unresectability is a tumour encasement of vital arteries such as the coeliac trunc or the superior mesenteric artery. In this case the only therapeutic option according to guidelines is a palliative chemotherapy or combined radiochemotherapy. Martin et al. could show that IRE is a safe method with a potentially improved overall survival in patients with locally advanced pancreatic cancer smaller 4cm in diameter when compared with palliative chemotherapy alone [3].

In our clinic we started IRE treatment in February 2015 and have up to date treated more than 150 patients with locally advanced pancreatic cancer who were not suitable for a surgical resection. We use an open surgical approach via transverse upper abdominal laparotomy under general anaesthesia. After opening of the omental bursa up to six needle-electrodes are directly inserted into the tumour guided by high-resolution ultrasound.

During the procedure the patient requires deep neuromuscular blockade to prevent muscular excitation. After adjusting the system for optimal energy delivery we apply 90 cycles of ultra-short (90ms) high-voltage (up to 3000V) pulses under cardiac synchronization aiming at approximately 30 amperes. After removing the needle-electrodes from the tissue, the puncture holes are sutured to prevent pancreatic fistulas. A drain is placed and checked for pancreatic enzymatic activity regularly on pod 1 and 3. On pod 6 a contrast enhanced CT-scan is performed to check for potential complications. This scan defines the baseline for further follow-up. Patients are usually dismissed 6-8 days post IRE.

Main potential complications are bleeding, chronic pain, delayed gastric emptying and pancreatic fistula [4]. Exclusion criteria for IRE treatment are metastatic disease, duodenal infiltration or infiltration of other surrounding organs and the presence of a bare metal stent in the common bile duct. Since long-term results are lacking IRE should only be used as palliative treatment option in unresectable pancreatic cancer and randomised, prospective studies are needed.

We will give an introduction of the IRE procedure in patients with locally advanced pancreatic cancer and give an insight in our 2,5 years of experience with the method.

**References**

1. Hidalgo M. **Pancreatic cancer.** N Engl J Med. 2010 Apr 29;362(17):1605-17. doi: 10.1056/NEJMra0901557.

2. Welsch T1, Büchler MW, Schmidt J. **Surgery for pancreatic cancer.** Z Gastroenterol. 2008 Dec;46(12):1393-403. doi: 10.1055/s-2008-1027790. Epub 2008 Dec 3.

3. Martin RC 2nd1, Kwon D, Chalikonda S, Sellers M, Kotz E, Scoggins C, McMasters KM, Watkins K. **Treatment of 200 locally advanced (stage III) pancreatic adenocarcinoma patients with irreversible electroporation: safety and efficacy.** Ann Surg. 2015 Sep;262(3):486-94; discussion 492-4. doi: 10.1097/SLA.0000000000001441.

4. Scheffer HJ1, Nielsen K2, de Jong MC3, van Tilborg AA3, Vieveen JM4, Bouwman AR4, Meijer S2, van Kuijk C3, van den Tol PM2, Meijerink MR3.

5. **Irreversible electroporation for nonthermal tumour ablation in the clinical setting: a systematic review of safety and efficacy.** J Vasc Interv Radiol. 2014 Jul;25(7):997-1011; quiz 1011. doi: 10.1016/j.jvir.2014.01.028. Epub 2014 Mar 18.

### 16:00 - 17:30 Lung Cancer Update

#### O14 Eighth revision of the TNM staging system for lung cancer

##### Theresa C. McLoud (Mcloud.theresa@mgh.harvard.edu)

###### Professor of Radiology, Harvard Medical School, Vice Chair for Education, Department of Radiology , Massachusetts General Hospital, Boston, Massachusetts, USA

The 8^th^ Edition of the TNM classification for lung cancer became effective in January 2017. It is based on retrospective and prospective data which included over 94,708 cases from 35 sources in 16 countries.

In regard to the T factor of tumour staging there has been a change in the categorization of tumour size. T1 now includes 3 categories of a, b, and c. T1a is ≤ 1 cm, T1b > 1 cm but ≤ 2 cm, and T1 c > 2 but ≤ 3 cm. The tumour must be surrounded by lung or visceral pleura with no invasion more proximal than the lobar bronchus. T2 tumours are > 3 cm but ≤ 5 cm. This category is further divided into categories 2a and 2b. Other features include involvement of a main bronchus at any distance from the carina or invasion of the visceral pleura and association with partial or complete lung atelectasis or pneumonitis. T3 tumours are considered > 5 cm but ≤ 7 cm. Other criteria include direct invasion of any of the following: parietal pleura, chest wall / superior sulcus, phrenic nerve, or parietal pericardium. The final criterion is separate tumour nodules in the same lobe. The criteria for T4 tumours are a size > 7 cm or invasion of any of the following: mediastinum, diaphragm, heart, great vessels, trachea, recurrent laryngeal nerve, esophagus, vertebral body, carina or separate tumour nodules in a different lobe but in the same lung.

In regard to the nodal (N designation of regional lymph node metastasis) the existing nomenclature is maintained. N0 indicates no regional lymph node involvement, and N1 ipsilateral peribronchial, hilar, intrapulmonary, nodal involvement N2 ipsilateral mediastinal and/or subcarinal nodal involvement, and N3 contralateral mediastinal, contralateral hilar, ipsilateral or contralateral scalene or supraclavicular nodal involvement.

The M indicator for metastatic disease contains a minor change. In the TNM 7 classification M1b represented all distant metastases. Now there are two categories for distant metastases, M1b is a single distant metastasis in a single organ and M1c indicates multiple distant metastases in a single or multiple organs. The M designation also includes separate tumour nodules in the contralateral lung as well as malignant pleural or pericardial involvement.

The current classification represents the continued improvement of previous modifications and reflects an evolving understanding of tumour behavior. It enables optimal treatment decisions.

**References**

Carter BW, Godoy MCB, Wu CC, Erasmus JJ, Truong MT. Current controversies in lung cancer staging. J of Thoracic Imaging. 31(4):201-214, July 2016.

Goldstraw et al. The IASLC Lung Cancer Staging Project: Proposals for the Revision of the TNM Stage Groupings in the 8^th^ Edition of the TNM Classification for Lung Cancer. J of Thoracic Oncology. 11:39-51, 2016.

#### O15 Unusual CT-morphology of early lung cancer

##### Massimo Bellomi^1,2^ , Marta Minotti^1^ , Cristiano Rampinelli^2^

###### ^1^Department of Oncology, University of Milano, Milan, Italy; ^2^Department of Radiological Sciences and Radiation Therapy, European Institute of Oncology, Milan, Italy

####### **Correspondence:** Massimo Bellomi (massimo.bellomi@ieo.it)

The typical presentation of early stage lung cancers on low-dose CT screening is non-calcified pulmonary nodules. However, there is a wide spectrum of unusual focal abnormalities that can be early presentations of lung cancer. These abnormalities include, for example, cancers associated with ‘cystic airspaces’ [1,2], or ‘non-nodular’ cancers presenting, for example, as fibrotic changes or scar-like lesions [3]. Such focal abnormalities are initial signs of lung cancer which usually develop over time in a lung nodule or suspicious pulmonary opacities and, therefore, should be checked at follow-up or annual repeat LDCT.

The detection of lung cancer with low-dose CT can be affected by the absence of intravenous contrast medium. As a consequence, endobronchial and central lesions can be difficult to recognise, raising the potential for missed cancers. Focal lesions arising within pre-existing lung disease, such as lung fibrosis or apical scars, can also be early lung cancer manifestations and deserve particular consideration as recognition of these lesions may be hindered by the underlying disease. Furthermore, the unpredictable growth rate of lung cancer, which ranges from indolent to aggressive cancers, necessitates attention to the wide spectrum of progression in lung cancer appearance on serial low dose CT scans.

There is a wide spectrum of low-dose CT findings in lung cancer screenings. Awareness of the various radiological features of early lung cancers and the familiarity with the low dose CT technique can improve the CT screening effectiveness and avoid missed diagnosis.

**References**

[1] Maki D, Takahashi M, Murata K et al Computed tomography appearances of bronchogenic carcinoma associated with bullous lung disease. J Comput Assist Tomogr 2006, 30:447–452

[2] MascalchiM, Attinà D, Bertelli E et al. Lung cancer associated with cystic airspaces. J Comput Assist Tomogr 2015, 39(1):102–108

[3] Xu DM, Yip R, Smith JP, Yankelevitz DF, Henschke CI IELCAP Investigators. Retrospective review of lung cancers diagnosed in annual rounds of CT screening. AJR Am J Roentgenol 2014, 203(5):965–972

#### O16 New solid nodules at lung cancer screening

##### Matthijs Oudkerk (m.oudkerk@umcg.nl)

###### CMI-Center for Medical Imaging EB45, University Medical Center Groningen, Groningen, The Netherlands

Currently, lung cancer screening by low-dose computed tomography (LDCT) is widely recommended for high-risk individuals by US guidelines, but there still is an ongoing debate concerning respective recommendations for European countries. Nevertheless, the available data regarding pulmonary nodules released by lung cancer screening studies could improve future screening guidelines, as well as the clinical practice of incidentally detected pulmonary nodules on routine CT scans. Most lung cancer screening trials present results for baseline and incidence screening rounds separately, clustering pulmonary nodules initially found at baseline screening and newly detected pulmonary nodules after baseline screening together. This approach does not appreciate possible differences among pulmonary nodules detected at baseline and firstly detected at incidence screening rounds and is heavily influenced by methodological differences of the respective screening trials. This presentation intends to create a basis for assessing non-calcified pulmonary nodules detected during LDCT lung cancer screening in a more clinical relevant manner. The aim is to present data of non-calcified pulmonary baseline nodules and new non-calcified pulmonary incident nodules without clustering them together, thereby also simplifying translation to the clinical practice of incidentally detected pulmonary nodules. Small pulmonary nodules newly detected at incidence screening rounds of LDCT lung cancer screening may possess a greater lung cancer probability than pulmonary baseline nodules at a smaller size, which is essential for the development of new guidelines.

### 16:00 - 17:30 Joint with DEGRO: Side Effects of Radiation Therapy

#### O17 Thoracic complications of radiation therapy

##### Stefan Diederich (stefan.diederich@vkkd-kliniken.de)

###### Department of Diagnostic and Interventional Radiology, Marien Hospital, Düsseldorf, Germany

Radiation therapy of malignancy in the chest may expose normal pulmonary parenchyma to radiation.

This may lead to radiation pneumonitis which may be clinically occult or present with symptoms such as dyspnoea, fever and dry cough depending on the proportion of lung parenchyma involved and individual susceptibility of the patient [1, 2, 3].

Radiation pneumonitis usually does not occur at lung doses below 30 Gray (Gy) whereas it develops almost always with doses above 40 Gy. In doses between 30 and 40 Gy individual features such as pre-existing lung disease, simultaneous chemotherapy or other medical therapy increase the likelihood of radiation pneumonitis.

Radiological abnormalities in radiation pneumonitis follow a typical time course: After the threshold dose has been reached there is a delay of 4 to 6 weeks with no radiological abnormalities. Subsequently ground glass opacities develop which increase in density to form consolidation within another 4 to 6 weeks. Consolidation persists for some months and resolves incompletely with residual fibrotic changes within the next weeks and months.

Following radiation therapy that did not lead to radiation pneumonitis initially, additional medical therapy with pneumotoxic drugs at a later stage may then cause manifest radiation pneumonitis (rebound/recall pneumonitis).

A pathognomonic feature of radiation pneumonitis is the fact that the location of ground glass opacities and consolidation precisely mirrors the radiation port, i.e. the area of pulmonary parenchyma exposed to radiation above the threshold dose while completely ignoring any anatomical borders such as segmental or lobar borders.

This is easily recognised with simple radiation ports such as anterior-posterior/posterior-anterior (AP-PA) radiation fields but is more difficult to recognise in modern complex radiation ports such as intensity-modulated radiation therapy (iMRT), stereotactic radiation therapy, cyber knife or gamma knife therapy etc. [4, 5].

Furthermore, radiation therapy of the chest is a recognised cause of organizing pneumonia (OP) [6]. In contrast to radiation pneumonitis consolidation or ground glass opacities in radiation-induced OP does not mirror the radiation port and may include pulmonary parenchyma outside the radiation field and has a very heterogeneous time course with OP developing only days but also moths after radiation therapy.

**References**

1. Movsas B, Raffin TA, Epstein AH, Link CJ Jr.: Pulmonary radiation injury. Chest 1997;111:1061-1076

2. Tsoutsou PG, Koukourakis MI: Radiation pneumonitis and fibrosis. Int J Radiat Oncol Biol Phys 2006: 1281-1293

3. Giridhar P, Mallick S, Rath GK, Julka PK: Radiation induced lung injury: prediction, assessment and management. Asian Pac J Cancer Prev 2015;16: 2613-2617

4. Choi YW, Munden RF, Erasmus JJ, Park KJ, Chung WK, Jeon SC, Park CK: Effects of radiation therapy on the lung: radiologic appearances and differential diagnosis. Radiographics. 2004;24:985-997

5. Bledsoe TJ, Nath SK, Decker RH: Radiation Pneumonitis. *Clinics in Chest Medicine 2017*, 38: 201-208

6. Akita K, Ikawa A, Shimizu S, Tsuboi K, Ishihara K, Sato S, Ueda R: Cryptogenic organizing pneumonia after radiotherapy for breast cancer. Breast Cancer 2005;12:243-247

#### O18 Abdominal complications of radiation therapy

##### Richard M. Gore, Kiran H. Thakrar, Silver RI, Daniel R. Wenzke, Gregory P. Jackson

###### Department of Radiology, North Shore University Health System-University of Chicago, Evanston, IL, USA

####### **Correspondence:** Richard M. Gore (rgore@uchicago.edu)

**INTRODUCTION:** Radiation therapy can damage any portion of the gastrointestinal tract and solid abdominal organs. The risk of injury is multifactorial: method of radiation delivery; size, number and frequency of radiation fractions; volume of irradiated tissue; and duration of therapy. In this presentation the protean manifestations of radiation therapy in the abdomen and pelvis are discussed

**LIVER:** Portions of the liver may be included in patients receiving external beam radiation therapy for gastric, biliary, pancreatic, and thoracolumbar spine malignancies. Those portions of the liver which receive more than 45 Gy may develop radiation hepatitis as a result of venooclusive disease with a clinical syndrome of anicteric ascites and hepatomegaly. On CT, the irradiated liver is hypodense with linear, well-defined margins that conform to the radiation port. On MR, the irradiated liver is oedematous causing T1-weighted hypointensity and T2-weighted hyperintensity.

**PANCREAS:** The pancreas is relatively radioresistant but stromal oedema and fibrosis have been reported. Chronic pancreatitis can be seen years after radiation therapy

**SPLEEN:** The spleen may be irradiated to treat lymphoma, leukemia, splenomegaly, and hypersplenism. Like lymph nodes, the spleen is very radiosensitive and doses of 4-8 Gy destroy lymphoid tissue within hours. Splenic atrophy and fibrosis can develop associated with functional hyposplenism and fulminant pneumococcal pneumonia.

**KIDNEYS AND URETERS**: The kidneys are radiosensitive and the risk of renal impairment increases with prior or concurrent chemotherapy. Renal morphology remains normal on imaging studies in the acute setting but ultimately poorly functioning atrophic renal parenchyma results. Compensatory hypertrophy of the nonirradiated kidney can develop. The ureters are radioresistant and strictures induced by radiation therapy are infrequent

**STOMACH AND DUODENUM:** Gastroduodenal radiation injury typically occurs with doses of 45-60 Gy administered over 5 weeks. In the acute phase, prepyloric or pyloric channel ulcers indistinguishable from peptic ulcer disease may develop. These ulcers, however, are typically refractory to therapy. In the chronic phase fixed narrowing, deformity, and an aperistaltic antropyloric region without ulceration can develop. Mural thickening and perigastric stranding may be seen on CT.

**SMALL BOWEL:** The small bowel is radiosenitive and three stages of radiation enteropathy corresponding to different gross and histopathologic changes have been reported. Acutely, during and immediately after therapy, hyperemia, ulcers and mucosal sloughing may occur producing mural thickening, submucosal edema and hemorrhage. In the subacute phase, 2-12 months after radiation therapy, crypt abscesses and ulcers may be seen. In the late phase, strictures, fistulas, and adhesive disease, which may lead to bowel obstruction can develop.

**COLON AND RECTUM:** The sigmoid colon and rectum are included in the radiation therapy portals for external beam therapy for gynecologic (particularly cervical carcinoma) as well as cancer of the prostate gland and bladder. Acutely, spasm, mucosal irregularity or nodular submucosal thickening may develop producing a sawtooth or cobblestone pattern on barium studies. Chronic radiation colitis causes structures and shortening of the colon, fistulae, sinus tracts, obstruction, or perforation. An increase in the presacral fat and submucosal edema or fat deposition can be seen on CT and MR.

**CONCLUSION:** An understanding of the spectrum of radiation induced injuries in the abdomen and pelvis is important in their differentiation from neoplastic, infectious and inflammatory disorders.

**References**

1. Kwek J-W, Iyer RB, Dunnington J, et al: **Spectrum of imaging findings in the abdomen after radiation therapy**. *AJR* 2006, **187:** 1204-1211.

2. Capps GW, Fulcher AS, Szucs RA, Turner MA: **Imaging features of radiation-induced changes in the abdomen.** *Radiograph*i*cs* 1997, **17:** 1455-1473.

3. Boudiaf M, Soyer P, Pelage J-P, et al: **CT of radiation-induced injury of the gastrointestinal tract: spectrum of findings with barium studies correlation.** *Eur Radiol* 2000, **10**: 920-925.

#### O19 Imaging of radiation-induced CNS toxicity

##### Giovanni Morana^1^, Elena Tornari^2^, Andrea Rossi^1^

###### ^1^Neuroradiology Unit, Istituto Giannina Gaslini, Genoa, GE, 16145, Italy; ^2^Department of Radiotherapy, Ospedale Policlinico San Martino, Genoa, GE, 16132, Italy

####### **Correspondence:** Giovanni Morana (giovannimorana@gaslini.org)

Radiation-induced CNS toxicity comprises a wide spectrum of clinical and radiological complications determining variable neurological manifestations.

Effects of brain radiation can be focal or diffuse and are influenced by different factors such as patient age, cumulative dose of irradiation, type of irradiation (i.e. accelerated vs hyperfractionated, photon vs proton therapy), duration of exposure and association with chemotherapy.

Classification of radiation-induced CNS toxicity is based on timing of clinical presentation and comprises three main categories: acute (within 1-6 weeks), subacute or "early" delayed (3 weeks to 6 months) and chronic or "late" delayed (after 6-12 months to years) [2].

Early injuries are usually reversible and tend to resolve spontaneously, whereas late delayed effects are generally irreversible.

Among radiation-induced complications pseudoprogression and radiation necrosis are respectively early and late delayed effects, enhanced by concomitant chemotherapy, that can be associated with clinical worsening mimicking disease progression [3,4].

Late delayed radiation-induced complications include also diffuse radiation leukoencephalopathy, vascular injuries, mineralizing microangiopathy and pituitary disfunction, which are extremely common in the paediatric population [5,6].

Conventional MRI is the imaging method of choice for evaluating radiation-induced brain alterations. Advanced imaging modalities such as, Diffusion Weighted Imaging, Magnetic Resonance Spectroscopy and Perfusion Weighted Imaging, as well as Positron Emission Tomography with amino-acid tracers, may be of help in differentiating pseudoprogression and radiation necrosis from true disease progression.

Evaluation and depiction of the main manifestations of brain injury induced by radiation therapy is the focus of the present work.

**References**

1. Tofilon PJ, Fike JR. The radioresponse of the central nervous system: a dynamic process. Radiat Res 2000, 153:357-70.

2. Pruzincová L, Steno J, Srbecký M, Kalina P, Rychlý B, Boljesíková E, et al. MR imaging of late radiation therapy- and chemotherapy-induced injury: a pictorial essay. Eur Radiol 2009, 19:2716-27.

3. Fatterpekar GM, Galheigo D, Narayana A, Johnson G, Knopp E. Treatment-related change versus tumour recurrence in high-grade gliomas: a diagnostic conundrum-use of dynamic susceptibility contrast-enhanced (DSC) perfusion MRI. AJR Am J Roentgenol 2012, 198:19-26.

4. Faraci M, Morana G, Bagnasco F, Barra S, Polo P, Hanau G, et al. Magnetic resonance imaging in childhood leukemia survivors treated with cranial radiotherapy: a cross sectional, single center study. Pediatr Blood Cancer 2011, 57:240-6.

5. Di Giannatale A, Morana G, Rossi A, Cama A, Bertoluzzo L, Barra S, et al. Natural history of cavernous malformations in children with brain tumours treated with radiotherapy and chemotherapy. J Neurooncol 2014, 117:311-20.

## Tuesday 3^rd^ October – Morning Session

### 09:00 - 10:30 Response Assessment

#### O20 RECIST – The Concepts and the Challenges

##### Aslam Sohaib (aslam.sohaib@rmh.nhs.uk)

###### Department of Imaging, Royal Marsden Hospital, Fulham Road, London, UK

Response Evaluation Criteria for Solid Tumours (RECIST) were introduced in 2000 to provide a standardised method for assessing response to treatments in the clinical trial setting [1]. The RECIST Working Group has updated RECIST to version 1.1 [2]. The revision addressed issues that had arisen related to the use of the criteria in practice. The revised version has maintained assessment of tumour burden using sum of the diameters and continues uses uni-dimensional measurements. The response categories are still those of complete response, partial response (30% decrease in sum from baseline), stable disease and progressive disease (20% increase in sum from nadir).

Though the RECIST criteria are intended for use in the clinical trial setting, onocologist increasingly rely on RECIST based measurements to make clinical management and therapeutic decisions in daily clinical practice. The reasons for this include clinician’s application of trial data into routine clinical practice but most importantly RECIST offers a simple way of measuring and communicating response assessment. The terms such as measureable disease, tumour burden, target lesions, and response categories are now used and understood universally. RECIST criteria also provide guidance on technique as well as measuring lesions and application to assessing sites of disease eg lymph node and bones. This allows for a more robust and reproducible way of gauging response.

RECIST guidelines are therefore widely employed, however, they have well recognised limitations and pitfalls. Tumours that are irregular, or show diffuse infiltration or poorly visualised eg with fatty liver, can all be difficult to measure in a reliable and reproducible way. Tumour with non-spherical growth pattern can be difficult to serially follow up and assess response. The categories for the response criteria are arbitrarily defined and may not correlate with clinical outcome. Morphological characteristics and tumour heterogeneity are not taken into consideration. Functional or physiological change with response is not assessed or measured in RECIST. Reliance of response is based exclusively on tumour size and clinical beneficial chemotherapeutic effects may occur without reduction in tumour size eg many new targeted therapies have anti-angiogenic effects and result in symptomatic improvement. Similarly immune mediated response may artificially result in short term increase in tumour size or small new lesions. Treatment effects such as necrosis, cystic change and haemorrhage can result in artificial change in tumour size. Mixed and differential response is simplified in the response assessment in RECIST. Bone disease without significant soft tissue and cystic tumour it can be difficult to assess change with the tumour.

The limitations in RECIST guidelines have led to the publications and developments in modified RECIST and other response criteria to overcome these shortfalls.

**References**

1. Therasse, P., et al., *New guidelines to evaluate the response to treatment in solid tumours. European Organization for Research and Treatment of Cancer, National Cancer Institute of the United States, National Cancer Institute of Canada.* J Natl Cancer Inst, 2000. **92**(3): p. 205-16.

2. Eisenhauer, E.A., et al., *New response evaluation criteria in solid tumours: revised RECIST guideline (version 1.1).* Eur J Cancer, 2009. **45**(2): p. 228-472.

3. Tirkes T, Hollar MA, Tann M, Kohli MD, Akisik F, Sandrasegaran K. *Response criteria in oncologic imaging: review of traditional and new criteria.* Radiographics. 2013. **33**(5):1323-41

#### O21 Resisting RECIST- new methods of assessing tumour response to therapy

##### Richard M. Gore, Daniel R. Wenzke, Kiran H. Thakrar, Robert I. Silvers RI, Gregory P. Jackson

###### Department of Radiology, North Shore University Health System-University of Chicago, Evanston, IL, USA

####### **Correspondence:** Richard M. Gore (rgore@uchicago.edu)

**INTRODUCTION:** Traditional chemotherapy is cytotoxic in nature and acts primarily by eliminating neoplastic cells. Change in tumour size, which is an indicator of change in the number of neoplastic cells, evolved into ***the*** radiologic biomarker of treatment response. Over the last decade, dramatic advances in understanding the genetics and molecular biology of tumours have revolutionised therapy for many neoplasms.

Molecular targeted therapy and immunotherapy have led to new, individualise tumour therapies. These interfere with signalling pathways and thereby inhibit cell grown but do not necessarily lead to cell death, unlike cytotoxic drugs. With targeted agents, lack of progression may be associated with a positive improvement in outcome, even in the absence of major shrinkage of tumours. Oncologists have become interested in the length of time that a cancer does not grow or metastasise. Progression free survival has become the preferred end point for many cancer therapy trials. In this presentation, new criteria for imaging tumour response in the era of personalised medicine are presented and guidelines for learning when it is appropriate to resist using RECIST are presented.

**CONVENTIONAL ANATOMIC CRITERIA**

WHO

RECIST

RECIST 1.1

**FUNCTIONAL CRITERIA**

Choi

Modified Choi

MAST

EASL

mRECIST

RECICL

irRC

SACT

**METABOLIC CRITERIA**

PERSIST

PET-CT

PET-MR

**EVOLVING IMAGING BIOMARKERS**

Perfusion

DWI

MRE

MRS

Volumetry

Growth kinetics

**CONCLUSION:** RECIST 1.1 is the mainstay of evaluating tumour response to therapy for most cancers

These criteria depend primarily in assessing changes in tumour dimensions but they do not reflect other morphologic, functional, or metabolic changes that may occur with novel chemotherapy, targeted or immunotherapy. It is important for radiologists to integrate new concepts in the evaluation of tumour response in this era of personalised medicine and to learn when it is necessary to resist using RECIST criteria.

**References**

1. Pinker K, Riedl C, Weber WA: **Evaluating tumour response with FDG PET: updates on PERCIST, comparison with EORTC criteria and clues to future developments.** *Eur J Nucl Med Mol Imaging.* 2017 Mar 30.

2. Seymour L, Bogaerts J, Perrone A, et al: **iRECIST: guidelines for response criteria for use in trials testing immunotherapeutics**. *Lancet Oncol.* 2017;**18** :e143-e152.

3. Krajewski KM, Braschi-Amirfarzan M, DiPiro PJ, et al: **Molecular targeted therapy in modern oncology: Imaging assessment of treatment response toxicities**. *Korean J Radiol* 2017;**18** :28-41.

### 09:00 - 10:30 Joint with ESTRO: MR-Guided Radiation Therapy

#### O22 Challenges for medical physics

##### David Mönnich^1,2^, Daniela Thorwarth^1,2^, Daniel Zips^2,3^

###### ^1^Section for Biomedical Physics, Department of Radiation Oncology, University Hospital and Medical Faculty, Eberhard Karls University Tübingen, Tübingen, Germany; ^2^German Cancer Consortium (DKTK), partner site Tübingen; and German Cancer Research Center (DKFZ), Heidelberg, Germany; ^3^Department of Radiation Oncology, University Hospital Tübingen, Tübingen, Germany

####### **Correspondence:** David Mönnich (david.moennich@med.uni-tuebingen.de)

A new generation of devices combining magnetic resonance imaging (MRI) and a medical linear accelerator (linac) for image guided radiotherapy (IGRT) is currently being introduced clinically. The state-of-the-art in IGRT is using integrated (on-board) cone-beam computed tomography (CBCT) devices to verify the correct positioning of the patient on the linac treatment table with respect to the treatment plan created before RT treatment. This method is limited by low soft tissue contrast in CT images, slow acquisition and additional radiation exposure of the patient. Although MR images from diagnostic scanners are routinely used during RT treatment planning to define tumour volumes, it is currently not standard of care to acquire MR images in the treatment room for patient positioning or on-line treatment plan adaptation.

The acquisition of MR images in treatment positioning of the patient in close temporal proximity or even during RT treatment has many potential advantages: 1) Soft tissue contrast is superior to CBCT images. This may allow a more precise localization of tumours and organs at risk before each treatment fraction and consequently lead to reduced safety margins, which are routinely added to the clinical tumour volume in the treatment planning process to account for positioning uncertainties. Therefore, less healthy tissue would have to be irradiated. 2) On-board MR imaging offers functional MR imaging techniques, such as diffusion weighted (DWI) and dynamic contrast enhanced (DCE). The potential benefits of these modalities in RT include early response assessment and focal dose escalation/de-escalation according to the heterogeneous biological properties of tumours. Ultimately, the promise of using on-board MR imaging in RT is that treatments become less toxic, more effective and can be delivered in less fractions.

From the physical and technological point of view the MR-Linac is challenging, because the devices interfere in many ways: 1) The RT treatment beam is generated by acceleration of electrons in the linac, which is affected by the MR B_0_ magnetic field as well as the radiofrequency excitation electromagnetic waves. 2) In some devices, the output photon beam of the linac has to cross the MR cryostat, which can cause scattering of the beam and create additional out-of-field radiation dose to the patient. Apart from this, photons are largely unaffected by the MR device. 3) When photons enter the patient, secondary electrons are created, which interact with the B_0_ field via the Lorentz force resulting in shifted beam profiles and electron return dose effects. These effects have to be taken into account in the treatment planning system and in dosimetric quality assurance. 4) For each RT treatment fraction a new treatment plan has to be created on-line, i.e. while the patient is in treatment position, due to the limited degrees of freedom to re-position the patient in the MR-Linac. 5) The geometric accuracy of MR images used for RT has to be very high, which is an RT specific requirement. Images must be undistorted, with a maximum error of 1-2 mm in the centre of the field of view.

#### O23 Opportunities for diagnostic radiology

##### Sergios Gatidis^1^, Mike Notohamiprodjo^1,2^

###### ^1^Department of Radiology, University Hospital Tuebingen, 72074, Tuebingen, Germany; ^2^The Radiology, 80331, Munich, Germany

####### **Correspondence:** Sergios Gatidis (sergios.gatidis@med.uni-tuebingen.de)

Radiation therapy has become an effective treatment option for a series of oncologic and non-oncologic disorders. A prerequisite for the optimal therapeutic outcome is the precise and complete definition of the target area while sparing radiation-sensitive adjacent anatomic structures. Medical imaging, mostly Computed tomography, is used for this purpose. The potential advantages of MRI, especially high soft-tissue contrast and thus better lesion delineation, have resulted in increased use of this imaging modality in the context of radiation treatment planning. Furthermore, MRI offers unique possibilities for further functional characterization of pathologies using numerous available techniques such as diffusion-weighted imaging or perfusion-weighted imaging. This allows for a biologic tumour characterization in vivo before, during and after radiation therapy offering information about therapeutic response and prognostic outcome.

This talk will give an overview of the use of medical imaging in radiation oncology with a focus on MRI and an outlook on PET/MR. Topics discussed will be planning of radiation therapy, pre-therapeutic tumour characterization and assessment of therapy response.

### 11:00 – 11:30 Keynote Lecture 2

#### O24 Immunotherapy: Imaging Redefined

##### Bernhard Gebauer (bernhard.gebauer@charite.de)

###### Charité – Universitätsmedizin Berlin, Campus Virchow-Klinikum, Augustenburger Platz 1, 13353 Berlin, Germany

Traditional chemotherapy is directly introducing tumour cell death and later shrinkage of tumour masses throughout the body. So response evaluation criteria, like WHO, RECIST or Lugano criteria, used for evaluation of response to chemotherapy rely on tumour shrinkage as a sign of efficacy (1-4). Additionally in these criteria the appearance of new lesions or progressing non-target lesions is indicating progress of disease independent of the changes in the target lesions.

Ipilimumab, a monoclonal antibody that blocks cytotoxic T-lymphocyte-associated antigen 4 (CTLA 4), was the first immunotherapeutic agent that showed in clinical phase II trials survival benefits and successful tumour therapy in patients with advanced malignant melanoma (5). In the study treating malignant melanoma it was shown that superficial and organ metastasis in some patients even increase in size or new lesions appear, whereas these patients later show very good response and survival benefit in the following course of the study. Following traditional response criteria these patients would have been assessed as progressive disease (PD) and excluded from the study (or dropped out from the progression free survival (PFS) group). This increase of size and appearance of the lesions is thought to be a result of lymphocyte and macrophage invasion into the tumour and is called “pseudoprogression”.

Using the results from the mentioned study new criteria, immune-related response criteria (irRC), were defined by Wolchok et. al. (5). These criteria base on bi-dimensional target lesion measurements analogue to WHO criteria. In contrast to other criteria new tumour lesions not directly trigger progressive disease (PD). The bi-dimentional measurements of new appearing lesions will be included into the sum of product tumour diameters (SPD) of the defined target lesions at baseline. Additionally every progressive disease (PD) must be confirmed by a repeated imaging with minimum 4 weeks interval. Both new rules should reduce the pseudoprogression in the tumour response assessment in immunotherapy.

In the last years these new rules have been integrated into the mono-dimensional RECIST criteria to evaluate response after immunotherapy. For these new mono-dimensional criteria no commonly accepted term exists and different abbreviations like immune-related RECIST (irRECIST), immune-modified RECIST or modified RECIST (mRECIST) are oublished.

In 2017 the RECIST author team published a modification for the RECIST system in immunotherapy called iRECIST with the main difference to differentiate between immune unconfirmed progressive disease (iUPD) and immune confirmed progressive disease (iCPD) (6).

Additionally immune response criteria for lymphomas have been integrated into the established response evaluation criteria, called LyRIC (7).

In the last years many new immunotherapeutic agents have been investigated in clinical studies, like pembrolizumab, nivolumab (anti-programmed cell death protein 1 (anti-PD1) antibodies), atezolizumab (anti-PD-L1 (programmed cell death-ligand 1)), ipilimumab (anti-CTLA 4), alemtuzumab (anti CD52), ofatumumab and rituximab (anti-CD20).

Current studies are investigating these checkpoint inhibitors of the immune system in colorectal cancer, malignant melanoma, breast cancer, non-small-cell lung carcinoma, bladder cancer, renal cell carcinoma, lymphoma and leukaemia.

**References**

1. Miller AB, Hoogstraten B, Staquet M, Winkler A. Reporting results of cancer treatment. Cancer. 1981;47(1):207-14.

2. Therasse P, Arbuck SG, Eisenhauer EA, Wanders J, Kaplan RS, Rubinstein L, et al. New guidelines to evaluate the response to treatment in solid tumours. European Organization for Research and Treatment of Cancer, National Cancer Institute of the United States, National Cancer Institute of Canada. J Natl Cancer Inst. 2000;92(3):205-16.

3. Eisenhauer EA, Therasse P, Bogaerts J, Schwartz LH, Sargent D, Ford R, et al. New response evaluation criteria in solid tumours: revised RECIST guideline (version 1.1). Eur J Cancer. 2009;45(2):228-47.

4. Cheson BD, Fisher RI, Barrington SF, Cavalli F, Schwartz LH, Zucca E, et al. Recommendations for initial evaluation, staging, and response assessment of Hodgkin and non-Hodgkin lymphoma: the Lugano classification. J Clin Oncol. 2014;32(27):3059-68.

5. Wolchok JD, Hoos A, O'Day S, Weber JS, Hamid O, Lebbe C, et al. Guidelines for the evaluation of immune therapy activity in solid tumours: immune-related response criteria. Clinical cancer research : an official journal of the American Association for Cancer Research. 2009;15(23):7412-20.

6. Seymour L, Bogaerts J, Perrone A, Ford R, Schwartz LH, Mandrekar S, et al. iRECIST: guidelines for response criteria for use in trials testing immunotherapeutics. Lancet Oncol. 2017;18(3):e143-e52.

7. Cheson BD, Ansell S, Schwartz L, Gordon LI, Advani R, Jacene HA, et al. Refinement of the Lugano Classification lymphoma response criteria in the era of immunomodulatory therapy. Blood. 2016;128(21):2489-96.

### 11:30 – 12:30 Radiogenomics in Female Cancer

#### O25 What radiogenomics can do for breast cancer

##### Sarah J Vinnicombe (s.vinnicombe@dundee.ac.uk)

###### Cancer Research, School of Medicine, University of Dundee, Dundee, DD1 9SY, UK

The term ‘radiogenomics’ was initially coined to describe the study of genetic variation and its relationship with response to radiotherapy, as it has been shown that certain single nucleotide polymorphisms are associated with radiation toxicity [1]. More recently, the term has come to denote correlation of the imaging characteristics of a given cancer with its gene expression profile (genomics). The concept of radiology-pathology correlation is not new to cancer imagers, who routinely use imaging features to make specific radiological diagnoses, for example of a certain cancer subtype. However, radiogenomics can be thought of as the correlation of advanced, quantitative imaging features (that are not necessarily appreciable to the naked eye) with gene expression profiles, enabling radiology-pathology correlation at the microscopic or subcellular level. Hence another commonly used term for this discipline, ‘imaging genomics’. It is not uncommon to see the term ‘radiomics’ used interchangeably with radiogenomics in the literature, but the former better reflects the methodology of feature extraction from radiological images.

Gene expression profiling is now an essential prerequisite for molecular stratification and precision medicine in oncology and the notion that imaging could act as a biomarker for genotype is a very attractive one. Genome-based cancer characterisation necessitates tissue sampling, and a small sample from a large heterogeneous lesion may not be representative of a given tumour, whereas systematic feature extraction from routinely acquired images enables lesions to be interrogated in their entirety, thus circumventing in part the problem of tumoural heterogeneity.

In their seminal paper from 2001, Sørlie et al. demonstrated that there are distinct molecular phenotypes of breast cancer that can be identified through gene expression profiling, and which dictate treatment strategy [2]. Through various exploratory and hypothesis-driven approaches, mostly using magnetic resonance imaging, it is becoming apparent that a radiogenomic approach, may enable imaging to capture the genomic diversity of breast cancer [3,4,5]. For example, textural analysis of contrast enhanced MR images can differentiate between ductal and lobular cancers [6]. It has also been shown that there is a relationship between dynamic contrast enhanced (DCE) breast MRI kinetics and the various molecular subtypes of breast cancer [4,7]. The ability to differentiate between differing subtypes of oestrogen-receptor positive breast cancers without recourse to gene expression profiling has clear implications for therapy.

As well as acting as an imaging surrogate for genomic analysis, radiogenomics may be able to predict the likelihood of response to neoadjuvant chemotherapy [8]. If validated, this would have a profound impact on patient management. Radiogenomic research may also yield important prognostic information; for example, certain DCE features at preoperative breast MRI are associated with an increased recurrence score, as measured with the 21-gene Oncotype-DX assay [9].

This lecture will discuss the potential applications as well as limitations of radiogenomic research in the management of breast cancer.

**References**

1. Barnett, G. C., Coles, C. E., Elliott, R. M., Baynes, C., Luccarini, C., Conroy, D., … West, C. M. L. (2012). Independent validation of genes and polymorphisms reported to be associated with radiation toxicity: A prospective analysis study. *The Lancet Oncology*, *13*(1), 65–77. http://doi.org/10.1016/S1470-2045(11)70302-3

2. Sørlie, T., Perou, C. M., Tibshirani, R., Aas, T., Geisler, S., Johnsen, H., … Børresen-Dale, A. L. (2001). Gene expression patterns of breast carcinomas distinguish tumour subclasses with clinical implications. *Proceedings of the National Academy of Sciences of the United States of America*, *98*(19), 10869–74. http://doi.org/10.1073/pnas.191367098

3. Yamamoto, S., Maki, D. D., Korn, R. L., & Kuo, M. D. (2012). Radiogenomic analysis of breast cancer using MRI: A preliminary study to define the landscape. *American Journal of Roentgenology*, *199*(3), 654–663. http://doi.org/10.2214/AJR.11.7824

4. Mazurowski, M. A., Zhang, J., Grimm, L. J., Yoon, S. C., & Silber, J. I. (2014). Radiogenomic Analysis of Breast Cancer: Luminal B Molecular Subtype Is Associated with Enhancement Dynamics at MR Imaging. *Radiology*, *273*(2), 365–372. http://doi.org/10.1148/radiol.14132641

5. Ha, R., Jin, B., & Mango, V. (2015). Breast Cancer Molecular Subtype as a Predictor of the Utility of Preoperative MRI. *AJR. American Journal of Roentgenology*, *204*, 1354–1360.

6. Waugh, S. A., Purdie, C. A., Jordan, L. B., Vinnicombe, S., Lerski, R. A., Martin, P., & Thompson, A. M. (2016). Magnetic resonance imaging texture analysis classification of primary breast cancer. *European Radiology*, *26*(2), 322–30. http://doi.org/10.1007/s00330-015-3845-6

7. Grimm, L. J., Zhang, J., & Mazurowski, M. A. (2015). Computational approach to radiogenomics of breast cancer: Luminal A and luminal B molecular subtypes are associated with imaging features on routine breast MRI extracted using computer vision algorithms. *Journal of Magnetic Resonance Imaging*, *42*(4), 902–907. http://doi.org/10.1002/jmri.24879

8. Teruel, J. R., Heldahl, M. G., Goa, P. E., Pickles, M., Lundgren, S., Bathen, T. F., & Gibbs, P. (2014). Dynamic contrast-enhanced MRI texture analysis for pretreatment prediction of clinical and pathological response to neoadjuvant chemotherapy in patients with locally advanced breast cancer. *NMR in Biomedicine*, *27*(8), 887–896. http://doi.org/10.1002/nbm.3132

9. Ashraf, A. B., Daye, D., Gavenonis, S., Mies, C., Feldman, M., Rosen, M., & Kontos, D. (2014). Identification of intrinsic imaging phenotypes for breast cancer tumours: preliminary associations with gene expression profiles. *Radiology*, *272*(2), 374–84. http://doi.org/10.1148/radiol.14131375

### 11.00 - 12.30 Joint with DEGRO: Assessing Therapy Response

#### O26 Biology of radiation effects

##### Lydia Koi^1,2^ (Lydia.Koi@uniklinikum-dresden.de)

###### ^1^OncoRay – National Center for Radiation Research in Oncology, Faculty of Medicine and University Hospital Carl Gustav Carus, Technische Universität Dresden, Helmholtz-Zentrum Dresden - Rossendorf, Dresden, Germany; ^2^Helmholtz-Zentrum Dresden - Rossendorf, Institute of Radiooncology – OncoRay, Dresden, Germany

Radiation leads to multiple biological effects on cells. Radiation affects living things on an atomic level, by ionizing molecules inside the microscopic cells. The main target to kill tumour cells with radiation is the DNA [1]. A biological damage results as a consequence of radiation interactions between atoms and the DNA as target of the cells. When ionizing radiation comes in contact with a cell there are two mechanisms how radiation ultimately damage cells. These two mechanisms are called direct and indirect (by producing free radicals) effects [2]. But not all living cells are equally sensitive to radiation. Those cells which are reproducing actively are more sensitive than those which are not. The biological effectiveness of radiation depends on the linear energy transfer, total dose, number of fractions and the radiosensitivity of the targeted cells or tissues. Approximately 50% of all cancer patients receive radiotherapy during their course of illness, either as a curative or palliative treatment to relieve the patients from symptoms such as pain caused by the cancer [3]. Understanding of tumour radiation biology, particularly the mechanisms of radiation sensitivity and resistance in tumour lesions and toxicity in normal tissues is a basis for effective clinical application of radiotherapy [4,5]

**References**

[1] Jorgensen TJ. **Enhancing radiosensitivity: targeting the DNA repair pathways.Cancer.** *Biol Ther* 2009 Apr;8(8):665-70.

[2] Wouters BG, Begg AC. **Irradiation-induced damage and the DNA damage response**. In *Basic Clinical Radiobiology*, 4th edition. Edited by Joiner M and van der Kogel A. UK. Hodder Arnold 2009 p11-26

[3] Datta NR, Samiei M, Bodis S. **Radiotherapy infrastructure and human resources in Europe – Present status and its implications for 2020**. *Eur J Cancer*. 2014 Oct;50(15):2735–43

[4] Baumann M, Krause M, Overgaard J, Debus J, Bentzen SM, Daartz J, Richter C, Zips D, Bortfeld T. **Radiation oncology in the era of precision medicine.** *Nat Rev Cancer*. 2016 Apr;16(4):234-49.

[5] Chen HHW, Kuo MT**. Improving radiotherapy in cancer treatment: Promises and Challenges**. *Oncotarget* 2017 Jun 8. doi: 10.18632/oncotarget.18409. [Epub ahead of print]

#### O27 Imaging features of response in bones

##### Dow-Mu Koh (Mu.Koh@icr.ac.uk)

###### Royal Marsden Hospital, Sutton, Surrey, SM2 5PT, UK

Metastatic bone disease is common, particularly in breast and prostate cancers. Bone marrow involvement is also typical in patients with multiple myeloma. Conventional CT and radionuclide bone scans are frequently used to detect and follow-up malignant bone disease. However, these techniques do not directly visualise the disease, but rely on identifying the reaction or destruction of the bone associated with the disease infiltration. Not surprisingly, these techniques have limited diagnostic sensitivity for detecting disease, and are also suboptimal for assessing treatment response.

The response of bone disease associated with significant soft tissue can be measured before and after treatment using standard size measurement criteria to determine treatment efficacy. Using radionuclide bone scan, subjective reduction of tracer uptake has been used to assess treatment response. The MD Anderson Criteria combines both size reduction on CT and reduction in tracer uptake on radionuclide bone scan as criteria for treatment response.

However, there are significant issues with using CT and radionuclide bone scan for response assessment. On CT, an increase in bone sclerosis may occur in both disease response and progression, making it difficult to assess disease status, especially in multiply treated patients. By comparison, radionuclide bone scans may not visualise disease confined to the bone marrow, thus resulting in erroneous disease assessment. Furthermore, post treatment “flare” response can occur in responding patients, thus confounding image interpretation. More recently, semi-quantitative bone scan index has been applied to measure disease response but the clinical utility of this remains limited.

More recently, diffusion-weighted MRI (DWI) has been shown to be an emerging technique to assess the treatment efficacy of bone disease. The technique detects cellular disease and quantifies the apparent diffusion coefficient (ADC) of the disease. Using whole body DWI, responders to treatment show reduction in diffusion tumour volume and an increase in the tumour ADC. The increase in tumour ADC values in responders is also associated with a decrease in histogram skewness and decrease in kurtosis. However, the magnitude of such changes varies with the types of treatment administered and their relative effects on cellular integrity. With successful treatment, an increase in marrow fat fraction may also be observed on chemical shift imaging.

## Tuesday 3^rd^ October – Afternoon Session

### 14:00 - 15:00 Update Contrast Media

#### O28 Gd deposition in the brain: so what?

##### Harriet C. Thoeny (Harriet.thoeny@insel.ch)

###### Deparment of Radiology, Neuroradiology and Nuclear Medicine, Institute of Diagnosti, Pedatric and Interventional Radiology Inselspital, University of Bern, Bern, Switzerland

Gd-based contrast agents are widely used in clinical practice since their initial approval by the FDA in 1988. These contrast agents were considered safe for all patients until 2006 when an association between gadolinium and nephrogenic systemic fibrosis (NSF) in patients with end stage renal disease has been detected (1,2). Nearly all patients who developed NSF underwent contrast-enhanced MRI with a linear (Omniscan, Magnevist, OptiMARK) and therefore less stable contrast agent. Thereafter, the contrast media safety committee of the European Society of Urogenital Radiology (ESUR) released new guidelines suggesting avoidance of these less stable agents in patients with impaired renal function (3). Thanks to the restricted use of lineal agents in this patient population no new cases of NSF were reported in the literature since 2009.

In 2014 T1-hyperintensities in the dentate nucleus and in the globus pallidus were observed after repetitive administration of linear gadolinium based contrast agents (Omniscan and Magnevist) in patients with normal renal function and it has been proven shortly thereafter that these T1-hyperintensities correspond to gadolinium deposition (4,5). In recent years many retrospective studies confirmed these findings and most of these T1-hyperintensitites were observed after administration of linear Gd-based agents (Omniscan, Magnevist,Multihance, Promovist). There are four different types of GD-based contrast agents differentiated by their chelate ligand, either macrocyclic or linear and ionic or non-ionic.

It is known that gadolinium is also deposited in the bone and even to a substantially higher extent (23 times) and that there is a direct correlation between the T1-hyperintensities in the brain (6,7). Studies have shown that all gadolinium based contrast agents deposit in the bone and brain however there is a big difference between agents with a significantly higher deposition of linear agents compared to macrocyclic contrast media.

Although up to date no symptoms in patients with T1-hyperintensities have been observed the Pharmacovigilance Risk Assessment Committee (PRAC) of the European Medicines Agency (EMA) recently suggested and confirmed restrictions in the use of linear gadolinium agents. Primovist and Multihance should only be used for liver scans and Magnevist only in a special formulation for intraarticular use as the concentration is very low. All other linear agents (Omniscan, OptiMARK and Magnevist) should be suspended. The macrocyclic gadolinium agents (Dotarem, ProHance and Gadovist) are more stable and have a lower propensity to release gadolinium than linear agents and can therefore continuously being used in their current indications but at the lowest doses to provide the necessary clinical information.

**References**

1. Grobner T (2006) Gadolinium–a specific trigger for the development of nephrogenic fibrosing dermopathy and nephrogenic systemic fibrosis? Nephrology Dialysis Transplantation 21 (4):1104-1108

2. Marckmann P, Skov L, Rossen K, Dupont A, Damholt MB, Heaf JG, Thomsen HS (2006) Nephrogenic systemic fibrosis: suspected causative role of gadodiamide used for contrast-enhanced magnetic resonance imaging. Journal of the American Society of Nephrology 17 (9):2359-2362

3. ESUR Contrast Media Safety Committee (2014) ESUR Guidelines on Contrast Media 9.0. ESUR Office Vienna.

4. Kanda T, Ishii K, Kawaguchi H, Kitajima K, Takenaka D (2013) High signal intensity in the dentate nucleus and globus pallidus on unenhanced T1-weighted MR images: relationship with increasing cumulative dose of a gadolinium-based contrast material. Radiology 270 (3):834-841

5. McDonald RJ, McDonald JS, Kallmes DF, Jentoft ME, Murray DL, Thielen KR, Williamson EE, Eckel LJ (2015) Intracranial gadolinium deposition after contrast-enhanced MR imaging. Radiology 275 (3):772-782

6. White GW, Gibby WA, Tweedle MF (2006) Comparison of Gd (DTPA-BMA)(Omniscan) versus Gd (HP-DO3A)(ProHance) relative to gadolinium retention in human bone tissue by inductively coupled plasma mass spectroscopy. Investigative radiology 41 (3):272-278

7. Murata N, Gonzalez-Cuyar LF, Murata K, Fligner C, Dills R, Hippe D, Maravilla KR (2016) Macrocyclic and other non–group 1 gadolinium contrast agents deposit low levels of gadolinium in brain and bone tissue: preliminary results from 9 patients with normal renal function. Investigative radiology 51 (7):447-453

#### O29 Liver specific contrast media: when and why

##### Giovanni Morana, Michele Fusaro

###### Radiological Department, General Hospital Ca’ Foncello, Treviso, Italy

####### **Correspondence:** Giovanni Morana (giovannimorana@gaslini.org)

Thanks to the complex histological anatomy of the liver it has been possible to develop several LSCM, targeted to different cellular lines (hepatocites, RES) and mechanism of intracellular uptake. However, mostly due to commercial reasons, only two LSCM targeted to the hepatocites are nowadays available (although in few countries also a RES-specific contrast agent is available), which share the same uptake mechanism.

Gadobenate dimeglumine (Gd-BOPTA, MultiHance, Bracco Imaging S.p.A., Milan, Italy) and Gadolinium ethoxybenzyldiethylenetriaminepentaacetic acid (Gd-EOB-DTPA, Shering AG, Berlin, Germany) demonstrate combined perfusion and hepatocyte-selective properties. Such compounds distribute initially to the vascular-interstitial compartment in an analogous manner to that of the conventional, extracellular CM. Thereafter, a fraction of the injected dose (4% for Gd-BOPTA, 50% for Gd-EOB-DTPA) is taken up into the hepatocytes causing an increase of the signal intensity of the hepatic tissue and eliminated via the biliary ducts (1).

Both contrasts give advantages in the diagnostic management of focal liver lesions (FLL): in the dynamic phase they give the same information of conventional CM, with higher signal for Gd-BOPTA (2), but a significant difference can be observed between the two LSCM, as with Gd-EOB-DTPA extraction by hepatocites starts few minutes after the injection, and a real late distribution phase cannot be observed, impairing the characterization of FLL with a significant desmoplastic or fibrotic component, such as cholangiocarcinoma or fibrous hemangiomas, which after this CM appear rapidly hypointense (3). On the contrary in the hepatobiliary phase (HBP), obtained at 20’ (Gd-EOB-DTPA) or 2-3 hours after the injection (Gd-BOPTA), the larger extracted fraction of Gd-EOB-DTPA (50%) give a higher enhancement of the liver in comparison to Gd-BOPTA (2), especially in cirrhotic patients, where the uptake of LSCM is inversely related to the amount of fibrotic changes (4).

In the HBP liver-specific properties add a great capacity to better identify small non-hepatocellular lesions (which appear markedly hypointense in the HBP) or to better characterise benign hepatocellular lesions (which usually appear iso- to hyperintense in the HBP) from malignant ones (usually not able to uptake the LSCM).

The behaviour in the HBP after LSCM differs among different FLL:

- Benign hepatocellular FLL, such as FNH, NRH, usually show uptake of LSCM (5, 6);

- Border-line hepatocellular FLL, such as Hepatic adenoma, usually do not show uptake of LSCM (5, 6);

- Non hepatocellular FLL, either benign (eg. Hemangioma) or malignant (cholangiocarcinoma, metastases), do not show uptake of LSCM (3, 7);

- In the cirrhotic liver, regenerative or low grade dysplastic nodules (LGDN) usually show uptake of LSCM, while HGDN or HCC do not show uptake of LSCM (8).

In these patients, LSCM are especially helpful in case of nodules with atypical appearance in the dynamic phase (hypovascular; lack of washout), as the hypointensity in the HBP is suspicious for malignancy (9).

- Finally, the elimination of the LSCM via the biliary pathway gives the possibility to further explore biliary diseases by using T1w images instead of MRCP, using functional information.

In conclusion, LSCM are an important tool to better identify and characterise FLL. In case of atypical imaging appearance of FLL at US or CT, their use is necessary. Moreover, in oncological patients as well as in cirrhotic patients their use improves the accuracy of MRI in the detection and characterization of FLL. Finally, some biliary diseases are better evaluated using the biliary elimination of LSCM.

**References**

1. Thian YL, Riddell AM, Koh D-M. Liver-specific agents for contrast-enhanced MRI: role in oncological imaging. Cancer Imaging. 2013;13(4):567-579.

2. Frydrychowicz A, Nagle SK, D’Souza SL, Vigen KK, Reeder SB. Optimized High-Resolution Contrast-Enhanced Hepatobiliary Imaging at 3T: A Cross-over Comparison of Gadobenate Dimeglumine and Gadoxetic Acid. Journal of Magnetic Resonance Imaging. 2011;34(3):585-594.

3. Kim B, Byun JH, Kim HJ, Won HJ, Kim SY, Shin YM, Kim PN. Enhancement patterns and pseudo-washout of hepatic haemangiomas on gadoxetate disodium-enhanced liver MRI. Eur Radiol. 2016 Jan;26(1):191-8.

4. Verloh N, Utpatel K, Haimerl M, Zeman F, Fellner C, Fichtner-Feigl S, Teufel A, Stroszczynski C, Evert M, Wiggermann P. Liver fibrosis and Gd-EOB-DTPA-enhanced MRI: A histopathologic correlation. Sci Rep. 2015; 5: 15408.

5. Morana G, Grazioli L, Kirchin MA, Bondioni MP, Faccioli N, Guarise A, Schneider G. Solid hypervascular liver lesions: accurate identification of true benign lesions on enhanced dynamic and hepatobiliary phase magnetic resonance imaging after gadobenate dimeglumine administration. Invest Radiol. 2011 Apr;46(4):225-39.

6. Grazioli L, Bondioni MP, Haradome H, Motosugi U, Tinti R, Frittoli B, Gambarini S, Donato F, Colagrande S. Hepatocellular adenoma and focal nodular hyperplasia: value of gadoxetic acid-enhanced MR imaging in differential diagnosis. Radiology. 2012 Feb;262(2):520-9.

7. Piscaglia F, Iavarone M, Galassi M, Vavassori S, Renzulli M, Forzenigo LV, Granito A, Salvatore V, Sangiovanni A, Golfieri R, Colombo M, Bolondi L. Cholangiocarcinoma in Cirrhosis: Value of Hepatocyte Specific Magnetic Resonance Imaging. Dig Dis. 2015 Oct;33(6):735-44.

8. Merkle EM, Zech CJ, Bartolozzi C, Bashir MR, Ba-Ssalamah A, Huppertz A, Lee JM, Ricke J, Sakamoto M, Sirlin CB, Ye SL, Zeng M. Consensus report from the 7th International Forum for Liver Magnetic Resonance Imaging. Eur Radiol. 2016 Mar;26(3):674-82.

9. Sano K, Ichikawa T, Motosugi U, Ichikawa S, Morisaka H, Enomoto N, Matsuda M, Fujii H. Outcome of hypovascular hepatic nodules with positive uptake of gadoxetic acid in patients with cirrhosis. Eur Radiol. 2017 Feb;27(2):518-525.

10. Nolz R, Asenbaum U, Schoder M, Wibmer A, Einspieler H, Prusa AM, Peck-Radosavljevic M, Ba-Ssalamah A. Diagnostic workup of primary sclerosing cholangitis: the benefit of adding gadoxetic acid-enhanced T1-weighted magnetic resonance cholangiography to conventional T2-weighted magnetic resonance cholangiography. Clin Radiol. 2014 May;69(5):499-508.

#### O30 Contrast-associated acute kidney injury – evolving concepts

##### Jay P. Heiken (heikenj@wustl.edu)

###### Mallinckrodt Institute of Radiology, Washington University School of Medicine, St. Louis, MO 63110, USA

Historically, contrast-associated acute kidney injury (CA-AKI) (also known as contrast-induced nephropathy [CIN]) has been defined as an acute deterioration of renal function after intravenous administration of contrast medium. Chronic kidney disease is the most important risk factor for CA-AKI [1]. The poorer the renal function, the higher the risk of CA-AKI [2, 3]. The patients at highest risk of CA-AKI are those with both chronic kidney disease and diabetes mellitus [4]. Patients with cancer have an increased risk of CA-AKI due to multiple factors including advanced age, nephrotoxic effects of anticancer therapies, metabolic disturbances, sepsis, hematopoietic stem-cell transplantation and dehydration due to vomiting, diarrhea and poor oral intake [5].

The rate of CA-AKI is 2-4 times higher in patients undergoing intra-arterial contrast medium injection (e.g., cardiac catheterization) compared with those who receive intravenous injection (e.g., computed tomography [CT]). The reasons for this difference are multiple. Patients undergoing cardiac catheterization are more likely to have diabetes and hypertension, and the volumes of contrast medium used for these procedures tend to be higher than for CT examinations [6]. In addition, the angiography procedure itself likely is an important factor, as studies have demonstrated high rates of cholesterol emboli in patients undergoing aortography [7, 8].

Multi-institutional studies of patients with chronic kidney disease undergoing intravenous contrast medium administration for CT have demonstrated CA-AKI rates that are quite low, ranging from 1% to 4% depending on the definition of CA-AKI used [9, 10]. Another multi-institutional trial demonstrated that even in patients with chronic kidney disease and diabetes, the rate of CA-AKI was only 5% [11].

A confounding variable that limits our understanding of CA-AKI is that very few studies have included control subjects who did not receive intravascular contrast medium [12, 13]. Studies that include control subjects demonstrate a similar incidence of AKI between the contrast medium and control groups. These studies indicate that other causes of renal dysfunction that happen to be temporally related to the administration of contrast material such as the concurrent administration of nephrotoxic drugs or fluctuations in blood pressure also can cause acute increases in serum creatinine. Furthermore, studies have documented substantial acute variations in serum creatinine levels among hospitalise patients [14, 15].

The results of studies that include control subjects who did not receive intravenous contrast material suggest that CA-AKI is either much less common than previously thought or might not occur at all; however, these retrospective studies are limited by potential selection bias. For example, patients who are selected to undergo unenhanced CT examinations might have more severe illnesses, additional comorbidities or exposure to nephrotoxic drugs compared with patients who are selected to receive intravenous contrast material. Matched cohort propensity score analysis is a statistical method by which selection bias can be reduced in a retrospective study by matching patients based on risk factors. A study using this methodology demonstrated that post-CT AKI was prevalent in both the unenhanced and contrast-enhanced groups, and it increased with increases in pre-CT serum creatinine [16].

In summary, iodinated intravenous contrast material is a risk factor for AKI, but many risk factors contribute to the development of AKI after CT examinations, regardless of whether iodinated contrast material is administered. Hydration remains the most effective measure to minimize the risk of CA-AKI [17].

**References**

1. Morcos SK, Thomsen HS, Webb JA. **Contrast-media-induced nephrotoxicity: a consensus report. Contrast Media Safety Committee. European Society of Urogenital Radiology (ESUR).** *Eur Radiol* 1999; **9:**1602-13.

2. Finn WF. **The clinical and renal consequences of contrast-induced nephropathy.** *Nephrol Dial Transplant* 2006; **21** (suppl 1):i2-10.

3. Davenport MS, Khalatbari S, Cohan RH, et al. **Contrast material-induced nephrotoxicity and intravenous low-osmolality iodinated contrast material: risk stratification by using estimated glomerular filtration rate.** *Radiology* 2013; **268:**719-28.

4. Parfrey PS, Griffiths SM, Barrett BJ, et al. **Contrast material-induced renal failure in patients with diabetes mellitus, renal insufficiency, or both.** *N Engl J Med* 1989; **320**:143-9.

5. Rosner MH, Perazella MA. **Acute kidney injury in patients with cancer**. *N Engl J Med* 2017; **376:**1770-81.

6. Katzberg RW, Barrett BJ. **Risk of iodinated contrast material-induced nephropathy with intravenous administration.** *Radiology* 2007; **243:**622-6.

7. Keeley EC, Grines CL. **Scraping of aortic debris by coronary guiding catheters: a prospective evaluation in 1,000 cases.** *J Am Coll Cardiol* **1998:**1861-5.

8. Ramirez G, O’Neill WM, Lambert R, et al. **Cholesterol embolization: a complication of angiography.** *Arch Intern Med* 1978; **138:**1430-2.

9. Thomsen HS, Morcos SK, Erley CM, et al. **The ACTIVE trial: comparison of the effects on renal function of Iomeprol-400 and Iodixanol-320 in patients with chronic kidney disease undergoing abdominal computed tomography.** *Invest Radiol* 2008; **43:**170-8.

10. Barrett BJ, Katzberg RW, Thomsen HS, et al. **Contrast-induced nephropathy in patients with chronic kidney disease undergoing computed tomography. A double-blinded comparison of Iodixnol and Iopamidol.** *Invest Radiol* 2006; **41:**815-21.

11. Kuhn MJ, Chen N, Sahani DV, et al. **The PREDICT study: a randomized double-blind comparison of contrast-induced nephropathy after low- or isoosmolar contrast agent exposure.** *AJR Am J Roentgenol* 2008; **191:**151-7.

12. Rao QA, Newhouse JH. **Risk of nephropathy after intravenous administration of contrast material: a critical literature analysis.** *Radiology* 2006; **239:**392-7.

13. McDonald JS, McDonald RJ, Comin J, et al. **Frequency of acute kidney injury following intravenous contrast medium administration: a systematic review and meta-analysis.** *Radiology* 2013; **267:**119-28.

14. Newhouse JH, Kho D, Rao QA, Starren J. **Frequency of serum creatinine changes in the absence of iodinated contrast material: implications for studies of contrast nephrotoxicity.** *AJR Am J Roentgenol* 2008; **191:**376-82.

15. Bruce RJ, Djamali A, Shinki K, et al. **Background fluctuation of kidney function versus contrast-induced nephrotoxicity.** *AJR Am J Roentgenol* 2009; **192:**711-18.

16. Davenport MS, Khalatbari S, Dillman JR, et al. **Contrast material-induced nephrotoxicity and intravenous low-osmolality iodinated contrast material.** *Radiology* 2013; **267:**94-105.

17. Barrett BJ, Parfrey PS. **Preventing nephropathy induced by contrast medium**. *N Engl J Med* 2006; **354:**379-86.

### 14:00 - 15:30 Interventions: Percutaneous

#### O31 Image Guided High Dose Rate Brachytherapy to treat primary or secondary liver cancer

##### D. Schnapauff (dirk.schnapauff@charite.de)

###### Charité, Universitätsmedizin Berlin, Department of Radiology, Augustenburger Platz 1, 13353 Berlin, Germany

In oncologic patients local minimally invasive tumour ablation has gained increasing attention and relevance. In the majority of cases thermal ablation techniques as radiofrequency ablatio (RFA) and microwave ablation (MWA) are used which have limitations in terms of large tumour size or risk of incomplete ablation due to cooling effects of adjacent vessels. To overcome these limitations image guided high-dose rate (HDR) brachytherapy has been developed with excellent results in terms of local tumour control even in large tumours exceeding the size of 5 cm diameter.

There is a large dependency of local recurrence rates depending on tumour entity with low recurrence rates of patients with hepatocellular carcinoma (around 5 percent) whereas patients with metastasis of colorectal cancer to the liver have a risk around 20 percent to develop a local tumour recurrence.

Local ablation is possible in patients with local circumscribed tumour or till a quantity of 5 lesions within the liver.

The presentation will discuss technical requirements of image guided brachytherapy, indications and its current clinical role in multimodal treatment of cancer.

#### O32 Image-guided interventions in prostate cancer

##### Jurgen J. Fütterer (Jurgen.Futterer@radboudumc.nl)

###### Department of Radiology and Nuclear Medicine, Radboudumc, Nijmegen, The Netherlands

Focal therapy is an emerging alternative treatment option, which offers great hopes in terms of cancer control and decreased morbidity for localised prostate cancer. The challenge of focal therapy is to treat only localised tumours, sparing the rest of the prostate, especially near the neurovascular bundles and the urethral sphincter, to minimise potential morbidity.

The focal therapy concept remains controversial because prostate cancer is a multifocal disease. However, 13–33% of patients have 1 prostate cancer or index lesion and would be eligible for focal therapy. Consistent with the “index lesion theory”, indicating that most tumours other than the index cancer, may not be of clinical significance, even more patients would be suitable.

Various minimal invasive techniques such as cryoablation, focal laser ablation, irreversible electroporation, high intensity focused ultrasound, radiofrequency ablation, brachytherapy and photodynamic therapy have been used to perform focal therapy in prostate cancer patients. At present, there is no consensus which treatment method is the best for focal therapy.

To be able to perform focal therapy, accurate and adequate image guidance is a prerequisite. MR imaging provides two key advantages for an effective focal treatment over other imaging modalities as ultrasound or computed tomography. First, its excellent soft tissue contrast and multi-planar imaging capabilities allow for clear visualization of the tumour and for accurate thermal applicator placement into the lesion. Second, MR thermometry allows to measure in real-time the spatial distribution of the temperature in tissue, which is important to estimate boundaries of irreversible tissue necrosis. For these reasons, MR imaging is perfectly suited to use for image guidance during focal therapy, because it can be used to localise the tumour, target it with probes, monitor and control the ablation procedure in real-time and to map tissue temperature.

#### O33 Interventional radiology in paediatric oncology

##### Derek J Roebuck (derek.roebuck@gosh.nhs.uk)

###### Department of Radiology, Great Ormond Street Hospital, London, WC1N 3JH, UK

Interventional oncology (IO) is considerably less developed in adults than in children. Although there are many plausible applications for IO in paediatrics, very few have been widely adopted. Various barriers have been identified, including the relatively small numbers of patients afflicted by each tumour type (making randomised controlled trials in paediatric IO essentially impossible), relatively good results of conventional treatment (making IO less necessary in children than in adults) and a strongly protocol- or trial-driven approach to cancer care in children [1].

The most widely used applications of interventional radiology in paediatric oncology are biopsy, central venous access and certain forms of supportive care [2]. Image-guided biopsy has largely replaced surgical biopsy for the diagnosis of extracranial solid tumours in children.

The therapeutic IO techniques that have become common in adult practice are still rarely used in children. A systematic review published in 2014 evaluated the reports of only 28 children treated with ablation techniques for malignant or aggressive benign lesions [3], although many more relevant publications have appeared since then.

Although treatment of malignant liver tumours in children with chemoembolization (TACE) was investigated as long ago as the early 1990s, with promising results [4], this technique is rarely applied in practice. There are three potential indications for TACE in children: conversion of unresectable tumours to resectability, as a bridge to transplantation, and as a part of palliation. None of these has so far been incorporated into any major paediatric liver tumour trial. The new intercontinental paediatric liver tumour trial [5], called PHiTT, will at least provide a mechanism for collecting data on TACE in children.

Speculative applications for IO in children include the use of radiofrequency ablation or cryoablation as an alternative to partial nephrectomy for treatment of bilateral nephroblastoma (Wilms’ tumour).

Finally, interventional radiology has a small role in the palliative care of children with cancer. Examples include techniques for the management of malignant pleural effusions or ascites, and methods for delivering analgesic drugs or nerve blocks.

**References**

1. Hoffer FA: **Interventional radiology; the future.** *Pediatr Radiol* 2011; **41** (Suppl 1): S201-S206.

2. Hoffer FA: **Interventional radiology in pediatric oncology.** *Eur J Radiol* 2005; **53**: 3-13.

3. Gómez Muñoz F, Patel PA, Stuart S, Roebuck DJ: **Systematic review of ablation techniques for the treatment of malignant or aggressive benign lesions in children.** *Pediatr Radiol* 2014; **44**: 1281-1289.

4. Malogolowkin MH, Stanley P, Steele DA, Ortega JA: **Feasibility and toxicity of chemoembolization for children with liver tumours.** *J Clin Oncol* 2000; **18**: 1279-1284.http://www.birmingham.ac.uk/research/activity/mds/trials/crctu/trials/phitt/index.aspx

### 16:00 - 17:30 Side Effects of Medical Therapy

#### O34 Pulmonary complications of molecular targeted therapy

##### Richard M. Gore, Silvers RI, Daniel R. Wenzke, Kiran H. Thakrar , Gregory P. Jackson

###### Department of Radiology, North Shore University Health System-University of Chicago, Evanston, IL, USA

####### **Correspondence:** Richard M. Gore (rgore@uchicago.edu)

**INTRODUCTION:** Over the last 10 years, the management of cancer patients has been revolutionised by the advances in molecular targeted therapy and immunotherapy with significant benefits for patient outcomes and comfort. These therapies however are associated with new toxicities and complications that: can be mild, moderate or life-threatening; may require alteration or cessation of therapy; or simulate disease progression. In this presentation, the various classes of molecular targeted and immunotherapy associated with pulmonary complications are reviewed and the drug-associated injuries and their differential diagnosis are presented.

**PNEUMONITIS:** Drug induced pneumonitis develops in up to 10% of patients on immunotherapy and remains a diagnosis of exclusion that must be differentiated from infection and malignant lung infiltration. Five different patterns have been described on CT: ground glass opacities with preserved bronchovascular markings; increased interstitial markings, interlobular septal thickening, peribronchovascular infiltration, subpleural reticulation, and honeycomb pattern in severe cases ; cryptogenic organizing pneumonia-like, with discrete patchy or confluent consolidation with or without air bronchograms, predominantly peripheral or subpleural in location; non-specific, with a mixture of nodular and other subtypes, not clearly fitting into other subtype classifications.

**BRONCHIOLITIS OBLITERANS:** There is myxoid fibrous tissue filling the distal bronchioles and extending into alveolar ducts and associated with inflammatory cells. On CT imaging findings include: bilateral regions of patchy consolidation or small irregular nodular opacities, bronchial wall thickening and dilation, and small pleural effusions.

**RADIATION RECALL PNEUMONITIS:** This is an inflammatory reaction in previously irradiated areas of lung producing well defined areas of alveolar consolidation, ground glass opacities or infiltrates corresponding to the radiation portals. This pneumonitis usually presents 3-4 months following radiotherapy and the patient presents with cough and dyspnea.

**PULMONARY VENO-OCCLUSIVE DISEASE**: Progressive occlusion of postcapillary pulmonary venules leads to increased pulmonary resistance, pulmonary hypertension, and right ventricular failure. CT findings include diffuse ground-glass opacification, septal thickening, peribronchial thickening, soft tissue oedema around the hila and mediastinum, small pleural effusions, and dilatation of the central pulmonary arteries.

**SARCOID-LIKE GRANULOMATOUS REACTIONS:** Intrathoracic lymphadenopathy simulating sarcoidosis develops in up to 10% of patients following ipilimuab and nivolumab therapy. The adenopathy may manifest and newly enlarged lymph nodes or enlargement of pre-existing lymph nodes that occur in isolation or associated with bilateral upper lobe and middle lobe predominant ground glass opacities, parenchymal consolidations and/or irregular nodules. Most patients are asymptomatic and biopsy show non-caseating granulomas with elevated CD4:CD8 levels. Extrathoracic diffuse adenopathy and cutaneous non-caseating granulomas have also been described.

**PSEUDOPROGRESSION:** Immunotherapy often may initially provoke infiltration of cytotoxic T lymphocytes and other immune cells into the tumour bed. This may cause an increase in tumour size or the development of new lesions as an early response. Pseudoprogression is defined as ≥ 25% increase in tumour burden that is not seen on repeat imaging performed 4 weeks or more after the initial study. Mixed immune-related responses or pseudoprogression are quite problematic in assessing treatment response using RECIST criteria.

**References**

1. Kroschinsky F, Stolzel F, von Bonin S, et al: **New drugs, new toxicities: severe side effects of modern targeted and immunotherapy of cancer and their management.** *Critical Care* 2017; **21**: 89—100.

2. Shannon VR: **Pneumotoxicity associated with immune checkpoint inhibitors**. *Curr Opin Oncol* 2017; **23**: 1-18.

3. Tabchi S, Messier C, Blais N: **Immune-mediated respiratory adverse events of checkpoint inhibitors**. *Curr Opin Oncol* 2017; **28:** 269-277.

#### O35 Acute abdominal complications of cancer therapy in children

##### Beth McCarville (Beth.mccarville@stjude.org)

###### Department of Diagnostic Imaging, St. Jude Children’s Research Hospital, Memphis, TN, USA

Acute abdominal complications in children treated for cancer can be life threatening and the early recognition of these conditions is imperative to patient safety and to guide cancer therapy. Radiologists interpreting the imaging of these patients should be aware of the types of complications that may occur, their clinical signs and symptoms and the imaging appearances of these conditions. The radiologist must direct the appropriate imaging work-up of these patients both for diagnosis and to monitor the effect of intervention. I will review the clinical and imaging features of the more common abdominal complications that occur at our large children’s cancer hospital. The discussion will include the rationale for which imaging modality should be used to assess these conditions, their key imaging features and the role of imaging in monitoring the management of these complications.

#### O36 How to differentiate between residual disease and treatment complications

##### Dow-Mu Koh (Mu.Koh@icr.ac.uk)

###### Royal Marsden Hospital, Sutton, Surrey, SM2 5PT, UK

Cancers are treated with surgery, chemotherapy, targeted therapies, radiotherapy and minimally invasive therapies, which can have effects on both normal and diseased tissues. Awareness of the acute, sub-acute and long term complications of such treatments can help in the appropriate interpretation of imaging findings at patient follow-up.

Residual disease or disease recurrence occurs as result of inadequate tumour clearance, clonal resistance, tumour re-growth or metastatic spread. Knowledge of prior treatment and the appearances on imaging can help to raise the alert on possible active disease requiring further treatment.

In the interpretation of imaging, knowledge of the “PATTERN” can help to distinguish between treatment complications and disease recurrence. [PATTERN: P = Knowledge of pattern of disease spread; A = associated clinical findings; T = type of treatment administered; T = timing of abnormality in relation to treatment; E = extent of abnormality/ disease before treatment; R = radiological features; N = use of novel functional imaging].

### 16:00 - 17:30 Interventions: Vascular

#### O37 TACE in metastases and hepatocellular carcinoma

##### Federico Collettini (federico.collettini@charite.de)

###### Department of Radiology, Charité Universitätsmedizin Berlin, Berlin, Germany

Hepatocellular carcinoma (HCC) and colorectal liver metastases (CRLM) represent the two most common types of cancer affecting the liver **[1]**. Curative treatments, including liver resection, have shown promising results in patients with primary and secondary liver cancer, but can only be offered to a minority of patients at time of diagnosis **[2, 3]**. In the last years, catheter-based intra-arterial therapies, including transarterial chemoembolization (TACE), have gained great interest for the treatment of unresectable liver tumours **[4]**. The rationale for the use of these therapies in patient with liver tumours relies on preferentially arterial blood supply of liver tumours, which allows delivery of the chemotherapeutic drug selectively through tumour-feeding artery while sparing the healthy liver parenchyma mainly supplied by the portal vein. Nowadays, TACE represents the treatment of choice for patients with intermediate-stage HCC and is increasingly used for patients with liver-dominant CRLM after failure of surgery or systemic chemotherapy **[4, 5]**. Since the time of its introduction as a treatment for patients with liver cancer more than three decades ago, TACE has been gradually improved, both with respect to angiographic technique, embolizing and drug delivery agents and image-guidance technology **[6]**. Herein we want to review the rationale, as well as the preclinical and clinical data supporting the use of different TACE techniques in patients with primary and secondary liver cancer. Furthermore, we will discuss the latest developments and their potential benefits in the field of transarterial chemoembolization.

**References**

1. Siegel R, Ma J, Zou Z, Jemal A. Cancer statistics, 2014. CA Cancer J Clin 2014; 64:9–29.

2. Mazzanti R, Gramantieri L, Bolondi L. Hepatocellular carcinoma: epidemiology and clinical aspects. Mol Aspects Med 2008; 29: 130-143.

3. Kemeny N. The management of resectable and unresectable liver metastases from colorectal cancer. Curr Opin Oncol 2010; 22: 364-373.

4. Forner A, Llovet JM, Bruix J. Hepatocellular carcinoma. Lancet. 2012; 379: 1245-55.

5. Fiorentini G, Sarti D, Aliberti C, Carandina R, Mambrini A, Guadagni S. Multidisciplinary approach of colorectal cancer liver metastases. World J Clin Oncol. 2017; 10:190-202.

6. Pesapane F, Nezami N, Patella F, Geschwind JF. New concepts in embolotherapy of HCC. Med Oncol. 2017 34:58.

#### O38 Transarterial Radioembolization to treat primary or secondary liver Cancer

##### D. Schnapauff (dirk.schnapauff@charite.de)

###### Charité, Universitätsmedizin Berlin, Departement of Radiology, Augustenburger Platz 1, 13353 Berlin, Germany

Hepatic radioembolization is a relatively new and developing modality for treating non-resectable primary or secondary liver tumours. Yttrium-90 coated microspheres are transarterially injected via a microcatheter into the hepatic artery. Preferential uptake into liver tumours is achieved by the predominant hepatic arterial supply whereas healthy liver tissue is predominantly perfused by the portal vein.

Radioembolization is currently indicated in patients with advanced tumour burden in a palliative situation with hepatocellular carcinoma, colorectal or neuroendocrine liver metastasis. Patients should have liver-dominant tumour burden and the liver must be estimated the life expectancy limiting organ.

First randomised trials like the SIRFLOX-trial investigating Y-90 microspheres in patients with previously untreated metastatic colorectal cancer did not an improvement of overall survival but a significantly delayed disease progression in the liver. Data of the SORAMIC-trial evaluating sorafenib ± Y-90 microspheres are not yet published, safety data published showed that the combination appears to be as well tolerated as sorafenib alone.

The presentation will discuss technical requirements for radioembolization and its current clinical role in multimodal treatment of cancer.

#### O39 Portal Vein Embolization

##### Bernhard Gebauer (bernhard.gebauer@charite.de)

###### Charité – Universitätsmedizin Berlin, Campus Virchow-Klinikum, Augustenburger Platz 1, 13353 Berlin, Germany

Portal Vein Embolisation (PVE) is an interventional procedure to increase the volume of future liver remnant (FLR) before extended liver resections to prevent early postoperative liver failure.

Liver surgeons normally prefer in healthy livers at least 25% (and 40% in diseased liver) as FLR after hepatic resection (1-4). So, in cases where a major hepatic resection (e.g. hepatic trisegmentectomy) is planned a preoperative volumetric measurement of hole liver volume and FLR is essential. The volumetric assessment of the FLR should be performed together with the surgeon to simulate his planned extent of resection. Additionally a test for the functional liver reserve should be performed (5).

PVE should reduce portal vein flow into the liver planned to be resected and increase the blood flow into the FLR. Together with the increased blood flow growths factors introduce a hypertrophy (approx. 30-50% hypertrophy is achievable with PVE) of the FLR, so acute liver failures in large resections could be reduced and prevented by PVE (6). Disadvantage of this cytokine and growth factor release is that it introduces growth of distant (and previously invisible) metastasis.

An alternative to PVE is the surgical in situ liver splitting and portal vein ligation or ALPPS (associating liver partition and portal vein ligation for staged hepatectomy) procedure (7, 8). In general, the amount of FLR hypertrophy in ALPSS is similar to PVE, but hypertrophy is faster and the growth of distant metastasis is reduced, but some centres report of an increased morbidity and mortality after ALPSS procedure (7-10).

PVE could be performed by percutaneous puncture of the ipsilateral (future resected) liver or by a contralateral (puncture of the FLR) approach. Some centres also use a transjugular-transhepatic approach similar to a TIPS procedure. The ipsilateral approach is the preferred access to the portal vein in PVE, because it safes the FLR and its portal vein from injury during the procedure. Different imaging techniques for puncture guidance could be used, we prefer ultrasound guidance to access ipsilateral portal vein. After selective catheterisation of the segmental portal veins branches the portal vein is embolised with different embolic materials (particles, gelatine, coils, plugs or glue) (11). This embolisation process is guided under fluoroscopy. After complete embolisation catheter and sheath tract should be embolised with glue to prevent bleeding and bile leakage into the peritoneum. Knowledge of typical and atypical portal vein anatomy and the extent of surgical resection is crucial for a successful PVE to introduce FLR hypertrophy in preparation for extended hepatic resections.

**References**

1. Iqbal S, Iqbal R, Iqbal F. Surgical Implications of Portal Vein Variations and Liver Segmentations: A Recent Update. J Clin Diagn Res. 2017;11(2):AE01-AE5.

2. Orcutt ST, Kobayashi K, Sultenfuss M, Hailey BS, Sparks A, Satpathy B, et al. Portal Vein Embolization as an Oncosurgical Strategy Prior to Major Hepatic Resection: Anatomic, Surgical, and Technical Considerations. Front Surg. 2016;3:14.

3. Madoff DC, Hicks ME, Abdalla EK, Morris JS, Vauthey JN. Portal vein embolization with polyvinyl alcohol particles and coils in preparation for major liver resection for hepatobiliary malignancy: safety and effectiveness--study in 26 patients. Radiology. 2003;227(1):251-60.

4. Madoff DC, Hicks ME, Vauthey JN, Charnsangavej C, Morello FA, Jr., Ahrar K, et al. Transhepatic portal vein embolization: anatomy, indications, and technical considerations. Radiographics. 2002;22(5):1063-76.

5. Lock JF, Westphal T, Rubin T, Malinowski M, Schulz A, Jara M, et al. LiMAx Test Improves Diagnosis of Chemotherapy-Associated Liver Injury Before Resection of Colorectal Liver Metastases. Ann Surg Oncol. 2017.

6. van Lienden KP, van den Esschert JW, de Graaf W, Bipat S, Lameris JS, van Gulik TM, et al. Portal Vein Embolization Before Liver Resection: A Systematic Review. Cardiovasc Intervent Radiol. 2012.

7. Donati M, Basile F, Oldhafer KJ. Present status and future perspectives of ALPPS (associating liver partition and portal vein ligation for staged hepatectomy). Future Oncol. 2015;11(16):2255-8.

8. Schnitzbauer AA, Lang SA, Goessmann H, Nadalin S, Baumgart J, Farkas SA, et al. Right portal vein ligation combined with in situ splitting induces rapid left lateral liver lobe hypertrophy enabling 2-staged extended right hepatic resection in small-for-size settings. Ann Surg. 2012;255(3):405-14.

9. Sun Z, Tang W, Sakamoto Y, Hasegawa K, Kokudo N. A systematic review and meta-analysis of feasibility, safety and efficacy of associating liver partition and portal vein ligation for staged hepatectomy (ALPPS) versus two-stage hepatectomy (TSH). Biosci Trends. 2015;9(5):284-8.

10. Nadalin S, Capobianco I, Li J, Girotti P, Konigsrainer I, Konigsrainer A. Indications and limits for associating liver partition and portal vein ligation for staged hepatectomy (ALPPS). Lessons Learned from 15 cases at a single centre. Z Gastroenterol. 2014;52(1):35-42.

11. Geisel D, Malinowski M, Powerski MJ, Wustefeld J, Heller V, Denecke T, et al. Improved hypertrophy of future remnant liver after portal vein embolization with plugs, coils and particles. Cardiovasc Intervent Radiol. 2014;37(5):1251-8.

## Wednesday 4^th^ October – Morning Session

### 09:00 - 10:30 How to Diagnose Focal Liver Lesions

#### O40 Practical approach to diagnosis of focal hepatic enhancing lesions

##### Khaled M. Elsayes (KMElsayes@mdanderson.org)

###### Department of Diagnostic Radiology, The University of Texas MD Anderson Cancer Center, Houston, Texas, USA

A broad spectrum of focal lesions can involve the liver and represent a daily challenge in the clinical practice [1]. These are usually detected and characterised contrast-enhanced computed tomography and magnetic resonance imaging which were proven to be reliable in the evaluation of various focal hepatic lesions. Practical diagnostic approach has been proposed for the diagnosis of these lesions [2]. Specifically, various enhancing lesions have been described in cirrhotic and non-cirrhotic livers. Vascular pitfall can mimic these neoplastic lesions [3] and will be also illustrated in this lecture.

**References**

1. Elsayes KM, Leyendecker JR, Menias CO, et al. MRI characterization of 124 CT-indeterminate focal hepatic lesions: evaluation of clinical utility. HPB (Oxford). 2007;9:208-15.

2. Elsayes KM, Narra VR, Yin Y, et al. Focal hepatic lesions: diagnostic value of enhancement pattern approach with contrast-enhanced 3D gradient-echo MR imaging. Radiographics. 2005;25:1299-320.

3. Elsayes KM, Shaaban AM, Rothan SM, A Comprehensive Approach to Hepatic Vascular Disease. Radiographics. 2017;37 (3):813-836.

#### O41 Rare and unusual liver tumours

##### Giovanni Morana, Michele Fusaro

###### Radiological Department, General Hospital Ca’ Foncello, Treviso, Italy

####### **Correspondence:** Giovanni Morana (giovannimorana@gaslini.org)

According to WHO (1), liver tumours can be subdivided into:

- Epithelial (benign and malignant)

- Non-epithelial (benign and malignant)

- Miscellaneous tumour (solitary fibrous tumour, teratoma, carcinosarcoma, Kaposi, etc.)

- Haematopoietic and lymphoid tumour

- Secondary tumours

Moreover, epithelial abnormalities (from benign to carcinoma in situ) and miscellaneous lesions (hamartoma, nodular regenerative hyperplasia, inflammatory pseudotumour) have been described.

We will discuss the imaging features of the most clinically relevant rare liver tumours (such as malignant vascular tumours, mucinous cystic neoplasms, sarcomas, primitive lymphomas).

In particular, MR features of these rare lesions will be discussed:

1. Primary hepatic angiosarcoma is a rare malignancy, but it is the most common primary mesenchymal tumour of the liver (1),

On an unenhanced CT angiosarcoma it can appear as a single dominant mass or multiple confluent nodules predominantly hypoattenuating compared to the surrounding liver, but it may contain either hyperattenuating areas, corresponding with a recent haemorrhage. On contrast-enhanced images, angiosarcoma appears extremely heterogeneous, with central or peripheral ring-shaped enhancement on arterial and portal phase and a progressive enhancement at delayed phase (2). On T1-weighted MR images, neoplastic nodules or masses can contain foci of high signal intensity in various degrees, corresponding to areas of intralesional haemorrhage at different age. On T2-weighted images, neoplastic nodules or mass can show foci of high signal intensity, representing necrosis, and solid area of low signal intensity, reflecting hemosiderin or fibrous solid portions (2).

2. Hepatic Epithelioid Hemangioendothelioma (HEH) is a rare malignant liver tumour with a vascular origin like angiosarcoma. Detection of HEH is usually incidental during an imaging study.

HEH may present itself as a single liver nodule or, more frequently, as a multinodular disease (HEH nodular type). HEH nodules, in late stage disease, coalesce in large infiltrative mass (HEH diffuse type). Prevalent right liver lobe involvement, peripheral location, tendency to coalesce of multiple nodules and capsular retraction are morphological features important for characterization, although they are not pathognomonic.

After administration of IV contrast material, some tumour nodules display marginal enhancement during the arterial phase. During portal and late phase the nodules appear hypodense or may become isodense to liver parenchyma (3).

At MR imaging on T2-weighted images, lesions have an inhomogeneous hyperintensity, with some lesions showing a target appearance, due to the presence of a central sclerotic zone and a peripheral region of cellular proliferation. On T1-weighted images the lesions are hypointense, with some lesions containing central areas of lower signal intensity than the remainder of the tumour (4).

After GBCA administration, a target enhancement occurs, with a central unenhancing area, due to the avascular stroma, and an enhancing rim represented by peripheral area with high cellularity and active proliferating tumoural epithelioid cells (5).

3. Biliary cystadenomas mainly affect women, range widely in age; its malignant counterpart biliary cystadenocarcinomas show no gender preponderance. However, also biliary cystadenoma is considered a premalignant condition.

They range in diameter from1.5 to 35 cm and usually grow slowly. Symptoms are usually related to the mass effect and are nonspecific.

At CT, biliary cystadenoma appears as a solitary cystic mass with a well-defined thick fibrous capsule, mural nodules, internal septa. On portal-venous-phase the lesion shows enhancement of the capsule and septa. Rarely, wall calcifications are seen (6).

At MR, uncomplicated biliary cystadenoma appears as a fluid-containing multilocular mass, with homogeneous low signal intensity on T1- weighted images and homogeneous high signal intensity on T2-weighted images. Signal intensity of mucinous fluids may vary depending on protein concentration or cystic haemorrhage (7). On T1-weighted images, the signal intensity may change from hypointense to hyperintense as protein concentration increases. On T2- weighted images signal intensity of mucinous fluids decrease from highly hyperintense to highly hypointense with increasing protein concentration. Fluid/fluid level secondary to internal haemorrhage is visible on both T1- and T2-weighted images.

On portal-venous-phase gadolinium-enhanced T1-weighted MR image shows enhancement of the capsule and septa.

In conclusion, several rare and unusual tumours can be found in the liver; knowledge of imaging findings allow a correct diagnosis in most cases.

**References**

1. Buetow PC, Buck JL, Ros PR, Goodman ZD (1994) Malignant vascular tumours of the liver: radiologic-pathologic correlation. Radiographics 14:153-66; quiz 167-8

2. Koyama T, Fletcher JG, Johnson CD, Kuo MS, Notohara K, Burgart LJ (2002) Primary hepatic angiosarcoma: findings at CT and MR imaging. Radiology 222:667-673

3. Miller WJ, Dodd GD III, Federle MP, Baron RL. Epithelioid hemangioendothelioma of the liver: imaging findings with pathologic correlation. AJR 1992; 159:53 –57

4. Paolantonio P, Laghi A, Vanzulli A, Grazioli L, Morana G, Ragozzino A, Colagrande S. MRI of hepatic epithelioid hemangioendothelioma (HEH). J Magn Reson Imaging. 2014 Sep;40(3):552-8

5. Lyburn ID, Torreggiani WC, Harris AC, Zwirewich CV, Buckley AR, Davis JE, Chung SW, Scudamore CH, Ho SG. Hepatic epithelioid hemangioendothelioma: sonographic, CT, and MR imaging appearances. AJR Am J Roentgenol. 2003;180(5):1359-64

6. Murphy BJ, Casillas J, Ros PR, Morillo G, Albores-Saavedra J, Rolfes DB (1989) The CT appearance of cystic masses of the liver. Radiographics 9:307-322

7. Buetow PC, Midkiff RB (1997) MR imaging of the liver. Primary malignant neoplasms in the adult. Magn Reson Imaging Clin N Am 5:289-318

#### O42 How to deal with liver incidentalomas

##### Jay P. Heiken (heikenj@wustl.edu)

###### Mallinckrodt Institute of Radiology, Washington University School of Medicine, St. Louis, MO 63110, USA

A liver incidentaloma usually is defined as an asymptomatic liver abnormality found serendipitously on an imaging study performed for another reason; however, another category of incidentaloma that is pertinent to the cancer imaging population is a liver lesion identified in a patient with newly diagnosed cancer. Distinguishing a benign from a malignant liver lesion in a patient with cancer can be difficult, as benign hepatic masses are very prevalent, increase with age and are found in more than 50% of adult autopsies [1]. An approach to dealing with liver incidentalomas begins with assessment of two pieces of information: 1) the imaging features of the liver lesion and 2) the risk category of the patient, based on which an appropriate management strategy can be determined.

General imaging features include whether the lesion is cystic, solid, hypoenhancing or hyperenhancing. More specific features include size, shape location, homogeneity, echogenicity, attenuation, signal intensity, margins, and enhancement pattern. Additional features include the presence of a central scar, hemorrhage, lipid or capsular retraction.

The American College of Radiology [2] stratifies individuals into three risk categories: low-risk, average-risk and high-risk. Low-risk and average-risk individuals have no malignancy, hepatic dysfunction, risk factors for hepatic malignancy or symptoms attributable to the liver. High-risk individuals have a known malignancy, cirrhosis or other hepatic risk factor. The only distinction between low-risk and average-risk individuals is age, those ≤40 years being considered low-risk, and those >40 years being considered average-risk. A simplified approach is to combine the low-risk and average-risk categories and consider individuals as either high-risk (known malignancy or other risk factor) or low-risk (no malignancy or other risk factor) [3].

If the imaging features of an incidentally discovered liver lesion indicate that it is benign, then no further evaluation is needed. If the imaging features are indeterminate, management options include follow-up, additional imaging to further characterise or biopsy. If the imaging features are strongly suggestive of malignancy, management options include additional imaging to further characterise, biopsy or treatment without tissue characterization.

When additional imaging is performed to further characterise an incidentally discovered liver lesion, magnetic resonance imaging (MRI) using a hepatobiliary contrast agent generally is considered the imaging technique of choice [3]. Alternative approaches include contrast-enhanced computed tomography (CT) or contrast-enhanced ultrasound (CEUS). Less commonly a nuclear medicine imaging study such as FDG-PET/CT, In-111-somatostatin receptor scintigraphy, technetium-99m sulfur colloid scan or technetium-99m red blood cell scan may be employed.

**References**

1. Karhunen PJ: **Benign hepatic tumours and tumour like conditions in men.** *J Clin Pathol* 1986; **39:**183-187.

2. Berland LL, Silverman SG, Gore RM, et al. **Managing incidental findings on abdominal CT: white paper of the ACR incidental findings committee.** *J Am Coll Radiol* 2010, **7:**754-773.

3. Nelson RC, Kamel IR, Baker ME, et al. **Liver lesion – initial characterization.** *American College of Radiology Appropriateness Criteria,* 2014.

#### O43 MRI of Cholangiocarcinoma

##### Kartik Jhaveri (Kartik.Jhaveri@uhn.ca)

###### Department of Medical Imaging, University of Toronto , University Health Network, Mt.Sinai & WCH, Toronto, ON M5G 2M9, Canada

Cholangiocarcinoma is a malignant neoplasm originating from the epithelial lining of the biliary tree and are the second most common primary hepatic neoplasm following hepatocellular carcinomas. Histopathologically, Cholangiocarcinomas represent an adenocarcinoma with a glandular appearance and variable differentiation. Cholangiocarcinomas most commonly occur in sixth decade of life and men are at slightly higher risk than women. Although most Cholangiocarcinomas arise sporadic and there is no recognized risk factor, the development of cholangiocarcinoma is higher in biliary duct disorders including primary sclerosing cholangitis (PSC), Caroli disease, choledochal cyst, parasitic infections (Clonorchis sinensis), toxins and viral hepatitis (especially type C). Cholangiocarcinoma demonstrate different presentations and outcomes based on their location and or pattern of growth and this has implications for treatment and prognosis Cholangiocarcinoma is classified according to its growth pattern: mass-forming, periductal-infiltrating, intraductal-growing type. From the stand point of location, they can be intrahepatic, perihilar, extrahepatic distal. Intrahepatic and extrahepatic tumours are separated by the second order bile ducts. The majority of Cholangiocarcinomas, 50-60%, occur at the common hepatic duct (CHD) and its bifurcation, also referred to as perihilar Cholangiocarcinoma (PHC), 20–30% are located distally and intrahepatic account for 20%. The risk of Cholangiocarcinoma contributes to poor prognosis and represents a major clinical challenge in PSC patients. In general, surveillance is used for disease monitoring in an asymptomatic risk population for the purpose of early tumour detection. Diagnosing biliary ductal cancer often requires a multi-modal approach combining imaging and laboratory modalities; although the use of high quality imaging techniques is promising for accurate pre-operative staging and assessment of tumour resectability. MR Imaging is the current standard for non-invasive assessment, pre-operative staging and surveillance. MRI protocol for Cholangiocarcinoma evaluation should include the liver and pancreas to exclude the non-hepatobiliary pathologies as the etiology of extrahepatic bile duct dilatation; also autoimmune pancreatitis (IgG4 disease) which can involve the biliary tract causing obstructing lesions mimicking Cholangiocarcinoma. Since complete resection of tumour remains the only therapy that offers the possibility of long-term survival, the need for precise pre-operative staging and assessment of resectability has accentuated the role of imaging in management. By taking full account of local tumour extent, the proposed staging system for extrahepatic cholangiocarcinoma accurately predicts resectability, the likelihood of metastatic disease, and survival. According to Blumgart T-staging system vascular involvement and liver parenchymal atrophy, both significantly affect the potential for resectability of extrahepatic cholangiocarcinoma and they were considered alongside with the longitudinal biliary extension. The specific criteria of unresectability for perihilar Cholangiocarcinoma according to Blumgart system include: (a) stage T3 (bilateral involvement of second-order biliary radicles or portal vein, or unilateral involvement of second-order biliary radicles in patients with incompetent liver either due to contralateral portal vein involvement or lobar atrophy) and (b) atrophy of one hepatic lobe with contralateral portal vein branch encasement or occlusion. Lymph nodes in pericholedochal, hilar, and portal locations are considered regional and their involvement is not a contraindication for surgical resection.

**References**

*1. Jhaveri K, Hosseini-Nik. MRI of cholangiocarcinoma. JMRI. 2015 Nov; 42(5):1165-792.*

*2. Lim JH. Cholangiocarcinoma: morphologic classification according to growth pattern and imaging findings. AJR Am J Roentgenol 2003; 181:819–827.*

*3. Park HS, Lee JM, Choi JY, et al. Preoperative evaluation of bile duct cancer: MRI combined with MR cholangiopancreatography versus MDCT with direct cholangiography. AJR Am J Roentgenol 2008; 190:396–405.*

*4. Menias CO, Surabhi VR, Prasad SR, Wang HL, Narra VR, ChintapalliKN. Mimics of cholangiocarcinoma: spectrum of disease. Radiographics 2008; 28:1115–1129.*

*5. Chapman MH, Webster GJ, Bannoo S, Johnson GJ, Wittmann J,Pereira SP. Cholangiocarcinoma and dominant strictures in patients with primary sclerosing cholangitis: a 25-year single-centre experience. Eur J Gastroenterol Hepatol 2012;24:1051–1058.*

*6. Jarnagin WR, Fong Y, DeMatteo RP, et al. Staging, resectability, and outcome in 225 patients with hilar cholangiocarcinoma. Ann Surg 2001; 234:507–517; discussion 517–509.*

*7. Ito F, Cho CS, Rikkers LF, Weber SM. Hilar cholangiocarcinoma: current management. Ann Surg 2009; 250:210–218.*

*8. Petrowsky H, Hong JC. Current surgical management of hilar and intrahepatic cholangiocarcinoma: the role of resection and orthotopic liver transplantation. Transplant Proc 2009; 41:4023–4035.*

### 09:00 - 10:30 Female Pelvis

#### O44 Imaging of Uterine Tumours

##### Hebert Alberto Vargas (vargasah@mskcc.org)

###### Department of Radiology, Memorial Sloan Kettering Cancer Center, New York, NY, USA

Uterine tumours originate from the endometrium, myometrium or cervix. Primary tumours at each of these sites demonstrate unique epidemiologic profiles, histologic subtypes and prognosis. The indications for imaging, the modalities used, and the imaging features are also specific to each of these primary sites. In this session, we will discuss the relevant features of uterine cancers and discuss how imaging can be used in the baseline and post treatment assessment of uterine tumours to guide management, prognosticate and document treatment response and recurrence.

#### O45 Incidental adnexal masses

##### Gabriele Masselli, Gianfranco Gualdi

###### Radiology Department, Umberto I Hospital, Sapienza University Rome, Rome, Italy

####### **Correspondence:** Gabriele Masselli (gabriele.masselli@uniroma1.it)

Incidentally discovered adnexal lesions are common, most are benign, but a minority can represent ovarian cancer, difficult to detect before it has spread. The frequency raised with increased use of cross-sectional imaging.

The point prevalence for significant cysts has been reported to be almost 8% in premenopausal women [1]. Surprisingly, the prevalence in postmenopausal women is as high as 14% to 18%, with a yearly incidence of 8%. From 30% to 54% of postmenopausal ovarian cysts persist for years [2].

The correct differentiation between malignant and benign lesions prevents unnecessary interventions and avoids delays in treatment of ovarian carcinoma.

US is the study of choice for primary evaluation of adnexal masses but it is less accurate for complex or indeterminate lesions, even when combined with color Doppler imaging.

The differential diagnosis for any particular mass or finding will vary based on the specific imaging features present, and ultrasound can be used to try to detect features that would enable a confident diagnosis or management strategy [3,4].

The most worrisome findings at US are the presence of solid areas that are not hyperechoic, especially when there is blood flow to them, thick septations, more than 2 or 3 mm wide, especially if there is blood flow within them, excrescences on the inner or outer aspect of a cystic area, ascites and other pelvic or omental masses.

Further evaluation with MRI may be critical for diagnosis, especially when it is not clear on US. MRI has been shown to be more specific and accurate than US and Doppler assessment.

MRI can determine, using frequency-selective fat saturation, whether a mass contains fat, which can be useful in the diagnosis of a teratoma. MRI can distinguish benign ovarian cysts from ovarian neoplasms because of the presence of papillary projections and nodular septa in neoplasms.

CT, and to a larger extent MRI, can show imaging features of adnexal masses that are characteristic or highly suggestive for particular diagnostic entities, such as paraovarian cyst, hydrosalpinx, peritoneal inclusion cyst, cystic teratoma, endometrioma, exophytic leiomyoma, ovarian fibroma, and ovarian carcinoma

MRI provides additional information on the composition of soft-tissue tumours. Usually, MRI is ordered with contrast, unless there are contraindications to it. The radiologist will evaluate morphologic features, signal intensity, and enhancement of solid areas. Techniques such as dynamic contrast-enhanced MRI (following the distribution of contrast material over time), in- and out-of-phase T1 imaging (looking for fat, such as in dermoids), and the diffusion-weighted imaging improve characterization.

Contrast-enhanced MRI contributes to a greater change in the probability of ovarian cancer than did CT, Doppler ultrasonography, or MRI without contrast. This may result in a reduction in unnecessary surgeries and in an increase in proper referrals in cases of suspected malignancy [5].

**References**

1. Borgfeldt C, Andolf E: Transvaginal sonographic ovarian findings in a random sample of women 25–40 years old. Ultrasound Obstet Gynecol 1999; 13:345–350.

2. Modesitt SC, Pavlik EJ, Ueland FR, DePriest PD, Kryscio RJ, van Nagell JR: Risk of malignancy in unilocular ovarian cystic tumours less than 10 centimeters in diameter. Obstet Gynecol 2003; 102:594–599.

3. Greenlee RT, Kessel B, Williams CR, et al: Prevalence, incidence, and natural history of simple ovarian cysts among women > 55 years old in a large cancer screening trial. Am J Obstet Gynecol 2010; 202:373.e1–373.e9.

4. Sharma A, Gentry-Maharaj A, Burnell M, et al: UK Collaborative Trial of Ovarian Cancer Screening (UKCTOCS). Assessing the malignant potential of ovarian inclusion cysts in postmenopausal women within the UK Collaborative Trial of Ovarian Cancer Screening (UKCTOCS): a prospective cohort study. BJOG 2012; 119:207–219.

5. Levine D, Brown DL, Andreotti RF, et al: Management of asymptomatic ovarian and other adnexal cysts imaged at US: Society of Radiologists in Ultrasound Consensus Conference Statement. Radiology 2010; 256:943–954.

### 09:00 - 10:30 Malignant Lymphoma

#### O46 Head and neck lymphoma

##### Ann D King (King2015@cuhk.edu.hk)

###### Department of imaging and interventional radiology, Chinese University of Hong Kong, Hong Kong

**Abstract**

The lecture will illustrate the diverse range of MRI appearances of non-Hodgkin’s lymphoma (NHL) in the head and neck, the 2^nd^ most common site of extra nodal lymphoma after the GI tract. Extra nodal NHL arises in lymphatic and extra lymphatic locations of the neck, and is often multifocal. NHL has a wide range of subtypes with clinical behaviour varying from indolent to highly aggressive disease. The clinical presentation and imaging may therefore mimic other pathologies in the head and neck, including autoimmune disease, infection and squamous cell carcinoma.

On MRI, most primary site NHLs form solid homogeneous contrast-enhancing masses with restricted diffusion, but a notable exception is NK/T-cell lymphoma may be necrotic. Common sites for NHL in the head and neck are Waldeyer’s ring, the sinonasal region, orbit & glands and oral cavity& facial bones.

*Waldeyer’s ring*: Waldeyer’s ring (palatine tonsil, tongue base, nasopharyngeal adenoid and palate) is the most common site of NHL in the head and neck and most are diffuse large B cell lymphomas (DLBCL). These tumours may grow to a large size without deep invasion, but DLBCL is an aggressive tumour which can involve contiguous sites along the aerodigestive tract and has a propensity to spread to nodes.

*Sinonasal region*: most sinonasal NHL are also DLBCL, but they show a greater propensity for local invasion and less nodal spread when compared to Waldeyer’s ring DLBCL. Sinonasal NHL frequently destroys bone or crosses the bone leaving the bone relatively intact. NK/T-cell lymphoma also has a predilection for the nasal cavity, which can manifest on imaging as subtle bands of mucosal thickening (easily overlooked), or invasive necrotic masses that erode the nasal septum and palate.

*Orbit & glands* (lacrimal, salivary and thyroid): these are frequent sites for mucosa-associated lymphoid tissue (MALT) lymphoma, an indolent often asymptomatic tumour with frequent relapse. The imaging features may be difficult to distinguish from chronic inflammation/ infection & autoimmune disease which are also predisposing factors for NHL.

*Oral cavity & facial bones*: includes Burkitt’s lymphoma, a tumour that is endemic in the African paediatric population and is one of the fastest growing human malignancies.

*Nodal groups*: accompanying neck nodes are often numerous involving multiple nodal groups. Nodal spread tends to be in an orderly fashion down the neck along expected routes, but also can involve unexpected groups such as those along the external jugular chain. Nodes tend to be well defined, solid and homogeneous, but necrosis and extracapsular spread are also found on MRI.

**References**

1. King AD et al. MRI of primary non-Hodgkin's lymphoma of the palatine tonsil. Br J Radios 2001;74(879):226-229.

2. King AD, et al. MRI of neck nodes in non-Hodgkin's lymphoma of the head and neck. Br J radio 2004;77(914):111-115.

3. Sandler A et al. Primary extranodal Non-Hodgkin lymphoma of the orbital and paranasal region-a retrospective study. Eur J Radiol 2013;82(2):302-308.

4. Kato H et al. Evaluation of imaging findings differentiating extranodal non-Hodgkin's lymphoma from squamous cell carcinoma in naso- and oropharynx. Clin Imaging 2013;37(4):657-663.

5. Aiken AH, Glastonbury C. Imaging Hodgkin and non-Hodgkin lymphoma in the head and neck. Radiol Clin North Am 2008;46(2):363-378.

6. King AD et al. MR imaging of nasal T-cell / natural killer cell lymphoma. American Journal of Roentgenology 2000; 174:209-211.

7. King AD et al. MRI of diffuse large B-cell non-Hodgkin's lymphoma of the head and neck: comparison of Waldeyer's ring and sinonasal lymphoma. Eur Arch Otorhinolaryngol. 2017;274:1079-1087.

#### O47 Staging and response assessment of lymphoma

##### Marius E. Mayerhöfer (marius.mayerhoefer@meduniwien.ac.at)

###### Dept. of Biomedical Imaging and Image-guided Therapy, Medical University of Vienna, Vienna, 1090, Austria

Lymphomas can be divided into Hodgkin (HL) and Non-Hodgkin lymphomas (NHL) on the one hand, and aggressive and indolent lymphomas on the other hand. Imaging plays a central role for the assessment of lymphomas, not only for Ann Arbor staging, but also for treatment response assessment.

While contrast-enhanced computed tomography (CE-CT) remains the most frequently used imaging test for lymphomas, the International Conference on Malignant Lymphoma (ICML) recommends the use of [18F]-FDG-PET/CT for the vast majority of lymphoma subtypes, including the most common ones: HL, diffuse large-B-cell lymphoma (DLBCL, most common aggressive NHL), and follicular lymphoma (most common indolent NHL) [1]. [18F]-FDG-PET/CT, which relies on the high glucose metabolism observed in most lymphomas, is superior to CE-CT, in particular for response assessment after chemo- or immune-chemotherapy. The set of rules for lymphoma response evaluation is currently the Lugano classification. For FDG-avid lymphomas, the Lugano classification relies chiefly on the so-called 5-point Deauville score, which is based on a comparison of the lesions’ FDG uptake on PET with the FDG uptake of reference tissues, especially the liver. Apart from this semi-quantitative evaluation, glucose metabolism on [18F]-FDG-PET can also be measured quantitatively through calculation of standardised uptake values (SUV).

Whole-body diffusion-weighted MR imaging (DWI) is slowly emerging as an alternative technique, providing results that are almost, though not quite, as good as those achieved with [18F]-FDG-PET/CT, both in terms of pre-therapeutic staging and treatment response assessment [2, 3]. It is based on the restriction of water movement in hypercellular tumours such as lymphomas, and is thus an indirect way of assessing cell density and size, and extracellular space tortuosity. As a radiation-free technique, it is particularly attractive for pediatric lymphoma patients, and for patients with lymphoma subtypes that are not, or only in a lower percentage of cases, FDG avid. The latter include marginal zone lymphomas such as MALT lymphoma, SLL/CLL (small lymphocytic lymphoma / chronic lymphocytic leukemia), Morbus Waldenström, and mycosis fungoides, for which the ICML currently does not recommend [18F]-FDG-PET/CT [1]. Similar to [18F]-FDG-PET, DWI has quantitative capabilities through the calculation of so-called apparent diffusion coefficients (ADC). However, compared to the SUVs on PET, ADCs tend to be less reliable, and are prone to motion artifacts.

Finally, PET/MRI has recently entered the clinical stage, and several studies have been performed in lymphoma patients. Due to the combination of the strengths of [18F]-FDG-PET and DWI, and the reduced radiation exposure – 6-8 mSv for PET/MRI, as opposed to about 20 mSv for PET/CT – PET/MRI may be regarded the universal, though very costly, imaging test for lymphoma, even though the actual benefit in routine clinical practice is only small, and is histology-dependent [4]. A definitive advantage over PET/CT lies in the fact that delayed time-point PET, which improves detection of some indolent lymphomas [5], can be easily integrated into the PET/MRI work-flow.

Among novel PET radiotracers used in lymphomas, [68Ga]-Pentixafor, which targets CXCR4 chemokine receptors [6], has great potential, because CXCR4, which mediates cell chemotaxis and migration, is overexpressed in lympho-proliferative diseases.

**References**

1. Cheson B, Fisher RI, Barrington SF, Cavalli F, Schwartz LH, Zucca E, Lister TA: **Recommendations for initial evaluation, staging, and response assessment of Hodgkin and non-Hodgkin lymphoma: the Lugano classification.** *J Clin Oncol* 2014;**32**:3059-3068.

2. Mayerhoefer ME, Karanikas G, Kletter K, Prosch H, Kiesewetter B, Skrabs C, Porpaczy E, Weber M, Pinker-Domenig K, Berzaczy D, Hoffmann M, Sillaber C, Jaeger U, Müllauer L, Simonitsch-Klupp I, Dolak W, Gaiger A, Ubl P, Lukas J, Raderer M: **Evaluation of diffusion-weighted MRI for pretherapeutic assessment and staging of lymphoma: results of a prospective study in 140 patients.** *Clin Cancer Res* 2014;**20**:2984-2993.

3. Mayerhoefer ME, Karanikas G, Kletter K, Prosch H, Kiesewetter B, Skrabs C, Porpaczy E, Weber M, Knogler T, Sillaber C, Jaeger U, Simonitsch-Klupp I, Ubl P, Muellauer L Dolak W, Lukas J, Raderer M: **Evaluation of diffusion-weighted magnetic resonance imaging for follow-up and treatment response assessment of lymphoma: results of an 18F-FDG-PET/CT-controlled prospective study in 64 patients.** *Clin Cancer Res* 2015;**21**:2506-2513.

4. Giraudo G, Raderer M, Karanikas G, Weber M, Kiesewetter B, Dolak W, Simonitsch-Klupp I, Mayerhoefer ME: **[18F]-FDG-PET/MR in lymphoma: comparison with [18F]-FDG-PET/CT and with the addition of diffusion-weighted MRI**. *Invest Radiol* 2016;**51**:163-169.

5. Mayerhoefer ME, Giraudo C, Senn D, Hartenbach M, Weber M, Rausch I, Kiesewetter B, Herold CJ, Hacker M, Pones M, Simonitsch-Klupp I, Müllauer L, Dolak W, Lukas J, Raderer M: **Does delayed-time-point imaging improve F-18-FDG-PET in patients with MALT lymphoma? Observations in a series of 13 patients**. *Clin Nucl Med* 2016;**41**:101-10

6. Herrmann K, Lapa C, Wester HJ, Schottelius M, Schiepers C, Eberlein U, Bluemel C, Keller U, Knop S, Kropf S, Schirbel A, Buck AK, Lassmann M: **Biodistribution and radiation dosimetry for the chemokine receptor CXCR4-targeting probe 68Ga-pentixafor**. *J Nucl Med* 2015;**56**:410-416.

#### O48 Paediatric lymphoma

##### Pek-Lan Khong (plkhong@hku.hk)

###### Department of Diagnostic Radiology, Queen Mary Hospital, The University of Hong Kong, Hong Kong

Malignant lymphomas comprise about 15% of childhood cancers, and are the third most common malignancy in the pediatric age group following leukemia and malignant brain tumours [1].

Hodgkin lymphoma (HL) shows a characteristic bimodal distribution, with the first, larger peak seen in adolescents and young adults (15-24 years), and a second smaller peak occurring in older adults. A cure rate of 95% is achieved for pediatric HL (0-14 years), which is overall more favourable than for adults. Unlike in adults, the non-Hodgkin lymphoma (NHL) subtypes are almost exclusively high grade lymphomas mostly of B-cell origin, and with frequent extranodal involvement. Four subtypes, Burkitt lymphoma (>80%), diffuse large B-cell lymphoma (10-20%), lymphoblastic lymphoma (T-cell > B-cell) and anaplastic large cell lymphoma are the most common. Overall survival rate now exceeds 80%. Challenges facing the management of lymphomas are the long term side effects of treatment, and thus, tailoring of therapy to reduce adverse effects and maintain high cure rates is an important goal in management.

In a meta-analysis, it was found that sensitivities and specificities of FDG-PET/CT for initial staging of paediatric lymphomas are 96%-99% and 95%-100%, respectively [2]. Compared to conventional staging methods, FDG-PET/CT provides higher diagnostic accuracy in lesion detection (96.7% versus 85.2%) and has been found to change disease stage in 15%-20% of patients [3, 4]. Moreover for HL, recent studies have suggested that bone marrow biopsy may be omitted due to the superior sensitivity of FDG-PET [5], or be limited to those with normal marrow uptake in the presence of poor risk factors and to exclude marrow involvement in the background of reactive marrow [6]. For CNS involvement, MRI with contrast remains the method of choice.

In treatment response assessment, FDG-PET/CT has been incorporated into the 2007 International Working Group guidelines for response assessment of HL and aggressive NHL [7], but that did not address pediatric lymphoma. In view of the clear differences in pediatric NHL, an international pediatric response criteria for pediatric NHL has been proposed in 2015. In this criteria, another category, CRu (unconfirmed) is applied in CR cases in which a residual mass on CT or MRI is negative by FDG-PET/CT and CR indicates disappearance of all disease, as confirmed by physical examination, imaging and examination of CSF and BM [8]. FDG-PET has been found superior to conventional imaging modalities for response assessment in allowing better discrimination between viable tissue and fibrotic residual masses by showing the altered metabolism, with high sensitivity and negative predictive value [9]. However, specificity and positive predictive value has been found to be low, reported to be 60% and 25% respectively in a cohort of NHL [10]. Thus, histological confirmation is recommended before treatment modification.

In evaluating paediatric lymphoma using FDG-PET/CT, pitfalls include FDG accumulation in; thymic rebound after chemotherapy, reactive cervical lymphadenopathy and prominent adenoids.

**References**

1) Howlader N, Noone AM, Krapcho M, Miller D, Bishop K, Kosary CL, Yu M, Ruhl J, Tatalovich Z, Mariotto A, Lewis DR, Chen HS, Feuer EJ, Cronin KA (eds). SEER Cancer Statistics Review, 1975-2014, National Cancer Institute. Bethesda, MD, https://seer.cancer.gov/csr/1975_2014/, based on November 2016 SEER data submission, posted to the SEER web site, April 2017.

2) Kabickova E, Sumerauer D, Cumlivska E, et al. Comparison of 18F-FDG PET and standard procedures for the pretreatment staging of children and adolescents with Hodgkin’s disease. Eur J Nucl Med Mol Imaging 2006;33:1025-1031.

3) London K, Cross S, Onikul E, et al. 18F-FDG PET/CT in paediatric lymphoma: comparison with conventional imaging. Eur J Nucl Med Mol Imaging 2011;38:274-284.

4) Cheng G, Servaes S, Zhuang H. Value of (18)F-fluoro-2-deoxy-D-glucose positron emission tomography/computed tomography scan versus diagnostic contrast computed tomography in initial staging of pediatric patients with lymphoma. Leuk Lymphoma 2013;54:737-742.

5) Purz S, Mauz-Körholz C, Körholz D, et al. [18F]Fluorodeoxyglucose positron emission tomography for detection of bone marrow involvement in children and adolescents with Hodgkin’s lymphoma. J Clin Oncol 2011; 29:3523-8.

6) Hassan A, Siddique M, Bashir H et al. 18F-FDG PET/CT imaging versus bone marrow biopsy in pediatric Hodgkin’s lymphoma: a quantitative assessment of marrow uptake and novel insights into clinical implications of marrow involvement. Eur J Nucl Med Mol Imaging 2017;44:1198-1206

7) Cheson BD, Pfistner B, Juweid ME et al. Revised response criteria for malignant lymphoma. J Clin Oncol 2007; 25:579-586.

8) Sandlund JT, Guillerman RP, Perkins SL et al. International pediatric non-Hodgkin lymphoma response criteria. J Clin Oncol 2015;33:2106-2111.

9) Furth C, Steffen IG, Amthauer H, et al. Early and late therapy response assessment with [18F]fluorodeoxyglucose positron emission tomography in pediatric Hodgkin’s lymphoma: analysis of a prospective multicenter trial. J Clin Oncol 2009;27:4385-4391.

10) Bhojwani D, McCarville MB, Choi JK, et al. The role of FDG-PET/CT in the evaluation of residual disease in paediatric non-Hodgkin lymphoma. Br J Haematol 2015; 168:845-53

### 11:00 – 12:30 Pancreatic Tumours

#### O49 MRI of pancreatic tumours

##### Kartik Jhaveri (Kartik.Jhaveri@uhn.ca)

###### Department of Medical Imaging, University of Toronto , University Health Network, Mt.Sinai & WCH, Toronto, ON M5G 2M9, Canada

MRI plays a crucial role in detecting pancreatic lesions and can provide useful information not applicable by any other imaging modalities. Incredible improvement in MRI techniques and the soft tissue contrast resolution makes it a strong method for characterization of pancreatic lesions and delineate normal pancreas from abnormal pancreatic tissue. Moreover, in comparison to other imaging modalities, MRCP provides a non-invasive technique for assessing the pancreaticobiliary ductal system. Normal pancreas is hyperintense on fat-suppressed T1 sequences compared to liver. Focal fatty infiltration demonstrates signal dropout on out-of-phase T1 images is a common finding that can mimic a mass on ultrasound and contrast enhanced CT scan. Although CT is the established imaging modality for detection and staging of pancreatic adenocarcinoma, it has a low sensitivity in diagnosis of small non-contour deforming tumours (<2 cm) and tumours that are isoindense to pancreatic parenchyma. On MRI, majority of pancreatic adenocarcinomas appear as low signal on non-contrast enhanced T1W fat suppressed sequences hypoenhancing on immediate post-gadolinium enhanced images. Larger tumours generally remain hypointense on delayed images whereas smaller tumours may become isointense or even hyperintense. The two radiologic signs “duct cut-off sign” and “double duct sign” are not pathognomonic for pancreatic adenocarcinoma as they are seen in other benign conditions such as chronic pancreatitis. Although studies showed an overall similar accuracy of MRI and MDCT for pre-operative staging, MRI may have priority in detecting metastatic disease involved liver and peritoneum. Another crucial application for MRI is for detecting neuroendocrine tumours. Pre-contrast fat suppressed T1 shows the lesions are low signal compared to background parenchyma and can be hyperenhancing on early as well as delayed post contrast images. Another special application of MRI is the characterization of internal structure of cystic neoplasms and determining connection to the ductal system which may be suggestive of a particular diagnosis. IPMNs are lobulated multicystic lesions typically found more common in elderly men. MRI may be more helpful than CT for differentiating IPMNs from other cystic masses, by identifying ductal communication of the cystic lesion. Gadolinium enhanced sequences demonstrate enhancing walls, intracystic or intraductal papillary nodules, and septae which may be features of malignancy. Mucinous cystic neoplasms may have features that cannot be differentiated on CT including varied cyst content manifesting as varying T1/T2 signal, subtle enhancing thickened walls and subtly enhancing septations. Serous cystadenomas are most commonly seen in middle age women with classic imaging appearance of a lobulated lesion composed of innumerable small cysts (<2 cm) giving the lesion a honeycomb or “spongy” appearance. In some cases, where the cystic components are very small and not discernable on CT or US, they can be well visualised on T2 weighted sequences. Solitary pseudopapillary tumours (SPT) are predominantly found among women. Small lesions are solid while larger tumours generally demonstrate cystic degeneration, necrosis and intracystic hemorrhage which are well characterised with MRI. Cystic degeneration of solid primary tumours or metastases may present as a cystic pancreatic mass such as with some neuroendocrine tumours.

**References**

*1. Heyn C, Sue-Chue-Lam D, Jhaveri K, Haider MA. MRI of the pancreas: problem solving tool. J Magn Reson Imaging. 2012 Nov; 36(5):1037-51*

*2. Prokesch RW, Chow LC, Beaulieu CF, Bammer R, Jeffrey RB, Jr. Isoattenuating pancreatic adenocarcinoma at multi-detector row CT: secondary signs. Radiology 2002;224(3):764-768.*

*3. Miller FH, Rini NJ, Keppke AL. MRI of adenocarcinoma of the pancreas. AJR Am J Roentgenol 2006;187(4):W365-374.*

*4. Bipat S, Phoa SS, van Delden OM, et al. Ultrasonography, computed tomography and magnetic resonance imaging for diagnosis and determining resectability of pancreatic adenocarcinoma: a meta-analysis. J Comput Assist Tomogr 2005;29(4):438-445.*

*5. Park HS, Lee JM, Choi HK, Hong SH, Han JK, Choi BI. Preoperative evaluation of pancreatic cancer: comparison of gadolinium-enhanced dynamic MRI with MR cholangiopancreatography versus MDCT. J Magn Reson Imaging 2009;30(3):586-595.*

*6. Ichikawa T, Sou H, Araki T, et al. Duct-penetrating sign at MRCP: usefulness for differentiating inflammatory pancreatic mass from pancreatic carcinomas. Radiology 2001;221(1):107-116.*

*7. Semelka RC, Custodio CM, Cem Balci N, Woosley JT. Neuroendocrine tumours of the pancreas: spectrum of appearances on MRI. J Magn Reson Imaging 2000;11(2):141-148.*

*8. Yu MH, Lee JY, Kim MA, et al. MR imaging features of small solid pseudopapillary tumours: retrospective differentiation from other small solid pancreatic tumours. AJR Am J Roentgenol;195(6):1324-1332.*

*9. Balci NC, Perman WH, Saglam S, Akisik F, Fattahi R, Bilgin M. Diffusion-weighted magnetic resonance imaging of the pancreas. Top Magn Reson Imaging 2009;20(1):43-47.*

#### O50 Incidental pancreatic cysts – what we know and what we don’t know

##### Jay P. Heiken (heikenj@wustl.edu)

###### Mallinckrodt Institute of Radiology, Washington University School of Medicine, St. Louis, MO 63110, USA

Pancreatic cysts are very common in the adult population, and the prevalence increases with age. Pancreatic cysts are identified in 13%-20% of adults undergoing abdominal magnetic resonance imaging (MRI) [1, 2] and in 2.6% of individuals undergoing multi-detector CT [3]. How we deal with these cystic lesions has important implications for both patient care and the expenditure of healthcare dollars.

Most pancreatic cysts discovered incidentally on imaging studies are branch duct intraductal papillary mucinous neoplasms (BD-IPMN). The vast majority are <3cm and have a very low risk of malignancy [4]. Nevertheless, certain imaging features of pancreatic cysts are associated with an increased risk of malignancy. The international consensus guidelines for the management of pancreatic mucinous neoplasms [4] identify a group of features which are characterized as “worrisome features” (size ≥3 cm, thickened enhanced wall, main pancreatic duct (MPD) size 5-9 mm, non-enhanced mural nodule(s), abrupt change in MPD caliber with atrophy of the upstream pancreas, and lymphadenopathy) and “high-risk stigmata” (obstructive jaundice in a patient with a pancreatic head lesion, enhanced solid component, and MPD size ≥10 mm).

Recent surgical data indicate that individuals with pancreatic cysts may be at increased risk of developing solid pancreatic neoplasms. 4%-10% of patients with resected BD-IPMN are found to have concurrent pancreatic ductal adenocarcinoma [5, 6]. In addition, patients with one or more pancreatic cysts have an increased risk of subsequent development of pancreatic ductal adenocarcinoma [7, 8].

Whether individuals with multifocal BD-IPMN are at increased risk of malignancy is unclear. Although most studies have not shown an increased risk of malignancy compared with solitary BD-IPMN, on study found that in 52 patients with ≥4 pancreatic cysts, 34% had high grade dysplasia or invasive carcinoma and 17% had a coexistent ductal adenocarcinoma [9].

Almost all of the data available on the biological behavior of pancreatic cysts come from surgical series and longitudinal imaging studies. No randomised controlled trials have been performed. Therefore our approach to the management of incidentally discovered pancreatic cysts is based on consensus guidelines developed by panels of medicine, surgery, pathology and imaging experts [4, 10, 11], and there is still a great deal to be learned about the genetics, natural history and risks associated with these cysts.

All of the consensus guidelines recommend that a cyst ≤3 cm should be followed as long as it contains no worrisome or high-risk features; however, what the follow-up schedule should be and when surgical intervention is indicated varies among the guidelines. Most consensus panels indicate that endoscopic ultrasound (EUS) with cyst aspiration should be considered to try to characterise cysts ≥2 cm.

It is important to bear in mind that the appearance of a pancreatic cyst on an imaging study does not translate directly into patient management. Appropriate management of an individual with an incidentally discovered pancreatic cyst depends upon multiple factors including age, comorbidities, the individual’s willingness to undergo surveillance or surgery, and family history of pancreatic cancer. Therefore management decisions should be individualised and based on multidisciplinary team discussion.

**References**

1. Zhang XM, Mitchell DG, Dohke M, et al. Pancreatic cysts: depiction on single-shot fast spin-echo MR images. *Radiology* 2002; **223:**547-53.

2. Lee KS, Sekhar A, Rofsky NM, Pedrosa I. **Prevalence of incidental pancreatic cysts in the adult population on MR imaging.** *Am J Gastroenterol 2010;* **105:**2079-84.

3. Laffan TA, Horton KM, Klein AP, et al. **Prevalence of unsuspected pancreatic cysts on MDCT.** *AJR 2008;* **191:**802-7.

4. Tanaka M, Fernández-del Castillo C, Adsay V, et al. **International consensus guidelines 2012 for the management of IPMN and MCN of the pancreas.** *Pancreatology* 2012; **12**:183-97.

5. Tanno S1, Nakano Y, Sugiyama Y, Nakamura K, et al. **Incidence of synchronous and metachronous pancreatic carcinoma in 168 patients with branch duct intraductal papillary mucinous neoplasm.** *Pancreatology* 2010; **10:**173-8.

6. Sahora K, Crippa S, Zamboni G, et al. **Intraductal papillary mucinous neoplasms of the pancreas with concurrent pancreatic and periampullary neoplasms.** *Eur J Surg Oncol* 2016; **42:**197-204.

7. Matsubara S, Tada M, Akahane M, et al. **Incidental pancreatic cysts found by magnetic resonance imaging and their relationship with pancreatic cancer.** *Pancreas* 2012; **41:**1241-6.

8. Chernyak V, Flusberg M, Haramati LB, et al. **Incidental pancreatic cystic lesions: is there a relationship with the development of pancreatic adenocarcinoma and all-cause mortality?** *Radiology* 2015; **274:**161-9.

9. Raman SP1, Kawamoto S, Blackford A, et al. **Histopathologic findings of multifocal pancreatic intraductal papillary mucinous neoplasms on CT.** *AJR* 2013; **200:**563-9.

10. Berland LL, Silverman SG, Gore RM, et al. **Managing incidental findings on abdominal CT: white paper of the ACR incidental findings committee.** *J Am Coll Radiol* 2010, **7:**754-773.

11. Vege SS, Ziring B, Jain R, et al. **American gastroenterological association institute guideline on the diagnosis and management of asymptomatic neoplastic pancreatic cysts.** *Gastroenterology* 2015; **148:**819-22.

#### O51 IPMN: field defect, precursor lesion of innocent bystander?

##### Richard M. Gore, Gregory P. Jackson, Kiran H. Thakrar, Robert I. Silvers, Daniel R. Wenzke

###### Department of Radiology, North Shore University Health System, University of Chicago Pritzker School of Medicine, Evanston, IL, 60201, USA

####### **Correspondence:** Richard M. Gore (rgore@uchicago.edu)

**INTRODUCTION:** Intraductal papillary mucinous neoplasms (IPMNs) are fairly prevalent pancreatic neoplasms that are characterised by intraductal papillary growths, thick mucus secretion, and pancreatic ductal dilation that are at risk for undergoing malignant transformation. The diagnosis, management, and treatment guidelines of this neoplasm are evolving. In this presentation, the etiopathogenesis, clinical, and imaging implications of this lesion are discussed.

**CLASSIFICATION:** The morphologic pattern of ductal dilation in IPMNs has been categorised into 4 major subtypes: diffuse main duct dilation; segmental main duct dilation, side branch dilation, and multifocal cysts with pancreatic duct communication. Each pattern has its own diagnostic, therapeutic, and prognostic implications.

**FIELD DEFECT**: It is well documented that patients with IPMNs are at increased risk of developing typical ductal adenocarcinoma of the pancreas both in association with and at a distance from the IPMN. Accordingly, recent ACR guidelines have extended the surveillance period for patients with IPMNs to 10 years. Discovery of an IPMN should prompt a careful search for concomitant pancreatic cancer.

**PRECURSOR LESION:** The reported incidence for malignant transformation of branch duct IPMNs ranges from 6-46% and 57-92% for main duct IPMNs.

**IMAGING FEATURES:** The following imaging features are concerning for malignancy in IPMNs: dilatation of the main pancreatic duct, particularly if > 7.2 mm; growing cysts and increasing main pancreatic duct diameter; cyst size > 3cm; enhancing mural nodules and nodules > 5 mm; thickened, irregular wall and septations; presence of or development of a solid enhancing component; diffusion restriction on diffusion weighted imaging; an SUV > 2.0 on PET/CT imaging; obstructive jaundice with a cyst in the pancreatic head.

**CONCLUSIONS:** CT and MRI are accurate in the diagnosis of malignant IPMNs. Future work is needed to better differentiate non-invasive and premalignant subtypes non-invasively.

**References**

1. Megibow AJ, Baker ME, Morgan DE, et al: **Management of incidental pancreatic cysts: a white paper of the ACR** *incidental findings committee. JACR* 2017 In Press

2. Choi S-Y, Kim JH, Yu MH, et al**: Diagnostic performance and imaging features for predicting the malignant potential of intraductal papillary mucinous neoplasm of the pancreas: a comparison of EUS, contrast-enhanced CT and MRI**. *Abdom Radiol* 2017, **42**:1449-1458.

3. Del Chiaro M. Verbeke C: **Intraductal papillary mucinous neoplasms of the pancreas: reporting clinically relevant features.** *Histopathology* 2017, **70**: 850-860.

#### O52 Pancreatic neuroendocrine tumours

##### Isaac R Francis (ifrancis@med.umich.edu)

###### Department of Radiology, University of Michigan, Ann Arbor, Michigan, USA

**Overview**

Pancreatic neuroendocrine tumours (PETs) have an incidence of 1 in every 100,000 subjects and account for approximately between 1%-2% of all pancreatic neoplasms {Ref.1]. They can be broadly divided into functioning and nonfunctioning tumours depending on associated clinical symptoms, and classified based on additional features such as size, histological features (mitotic index, Ki-67) and biological behavior. PNET’s have been classified by WHO into Grade I, benign-tumour is confined to the pancreas, <2 cms, no local invasion, < 2 mitoses per 10 HPF and < 2% Ki-67 cells, Grade 2- low grade-when there is gross local invasion or metastasis and Grade 3, high grade malignant- > 10 mitoses per HPF [Ref.2].

Most PETs are sporadic in nature, but it is thought that approximately 1%-2% are associated with familial syndromes such as MEN Type 1, VHL, NF Type 1, and tuberous sclerosis, where the tumours are often multiple [Ref.2]. With the increased use of cross sectional imaging, nonfunctioning tumours are now commonly being detected.

Functioning tumours can be divided based on type of hormonal secretion and symptoms into: Insulinoma, Gastrinoma (Zollinger-Ellison syndrome), Glucagonoma (4D syndrome), Vipoma (WDHA syndrome), and Somatostatinoma.

**Imaging appearances of PNETs**

On both CT and MRI, smaller PNETs are typically hyperattenuating/hyperintense lesions on both arterial and portal venous phase images. Larger tumours show areas of heterogeneous enhancement as well cystic change [Refs.3, 4]. Approximately 10% show a predominantly cystic tumour with rim enhancement. While studies have shown higher sensitivity for tumour detection on arterial phase imaging, both arterial and venous phases are complementary, as some lesions are best seen in the venous phase. On MR, PNETs are usually hypointense and hyperintense to pancreatic parenchyma on T1 and T2 images respectively. More recently studies have shown some added value with the use DWI and ADC to differentiate the different grades of PNETs [Ref 5]. In addition, tumour size > 2 cms, presence of calcification, vascular invasion and main pancreatic duct dilatation, appear to predict aggressive biologic behavior [Ref.6]. The recent development of the tracer Octreotide-DOTA has increased the use of this Gallium labelled agent for tumour detection and staging as well as in the follow up of these patients.

**Diagnosis, Treatment Options and Follow up Strategies**

Depending on symptomatology, various biochemical testing can be performed for the diagnosis of gastrinomas and insulinomas. Chromogranin A is a useful tumour marker that can be estimated in subjects with nonfunctioning PNETs for diagnosis and follow up [Ref.7].

Multiphasic contrast-enhanced CT or MRI are the initial tests of choice for the detection and staging of PNETs. Gallium-DOTA derivatives also play an important role in diagnosis, staging as well as in the follow up of patients with PNETs. Endoscopic ultrasound may be useful in detecting multiple small functioning tumours in the pancreas and the adjacent gastroduodenal wall (gastrinoma triangle).

Small (<2 cms) confined to the pancreas are usually treated with tumour enucleation or in cases with local tumour extension and where feasible, partial pancreatic resection is performed. However, there is growing but controversial trend towards non operative management for a small subgroup of patients with asymptomatic, incidental, small (< 2 cms) well-defined Grade I tumours [Ref.8].

For patients with metastatic disease, somtatostatin analogues, hepatic arterial embolization for liver metastases and more recently peptide receptor-targeted therapy with Yttrium labeled DOTA are being used.

**References**

1. Hruban RH, Pitman MB, Klimstra DS. Tumours of the pancreas: In: Silverberg SG, Sobin LH, eds. AFIP Atlas of tumour pathology. 4^th^.ed. Washington, DC: American Registry of Pathology 2007.

2. Lewis RB, Lattin GET, Pall E. Pancreatic endocrine tumours: Radiologic-pathologic correlation. Radiographics 2010, 30: 1445-1464.

3. Buetow PC, Miller DL, Parrino TV, Buck JL. Islet cell tumours of the pancreas: clinical, radiologic, and pathologic correlation in diagnosis and localization. Radiographics. 1997, 17(2); 453-472.

4. Semelka RC, Custodio CM, Cem Balci N, Woosley JT. Neuroendocrine tumours of the pancreas: spectrum of appearances on MRI. J Magn Reson Imaging 2000, 11(2); 141-148.

5. Guo C, Chen X, Xiao W, Wang Q, Sun K, Wang Z. Pancreatic neuroendocrine neoplasms at magnetic resonance imaging: comparison between grade 2 and grade 1 /2 tumours. Onco Targets and Therapy 2017, 10; 1465-1474

6. Canellas R, Lo G, Bhowmil S, et al. Pancreatic Neuroendocrine tumour: correlations between MRI features, tumour biology, and clinical outcomes after surgery. J Magn Reson Imaging 2017,00;000-000.

7. Falconi M, Erikkson B, Kaltsas G et al. Consensus guidelines update for the management of functional p-NETs and non-functional p-NETs. Neuroendocrinology 2016, 103(2):153-171.

8. Gajoux S, Partelli S, Maire F et al. Observational study of natural history of small sporadic nonfunctioning pancreatic neuroendocrine tumours. J Clin Endocrinol Metab, 2013, 98(12); 4784-4789.

### 11.00 - 12.30 Metastatic Disease

#### O53 Liver metastases: what the radiologist needs to know

##### Wolfgang Schima (Wolfgang.Schima@khgh.at)

###### Department of Diagnostic and Interventional Radiology, Goettlicher Heiland Krankenhaus, Barmherzige Schwestern Krankenhaus, and Sankt Josef Krankenhaus, Vinzenzgruppe, Vienna, Austria

Liver metastases are much more common than primary malignant tumours of the liver. The diagnosis is based on imaging findings (and biopsy). Radiology plays a key role in the management of patients with suspected liver metastases by detecting or excluding liver metastases. Ultrasound is widely available, but it has limitations in the detection of metastases due to operator and patient dependence, and contrast-enhanced ultrasound (CEUS) is not performed for metastasis detection on a routine basis.

The standard imaging technique for staging in patients with extrahepatic neoplasms at high risk for developing liver metastases (i.e., colo-rectal, gastric, oesophageal, pancreatic cancer, etc.) is contrast-enhanced MDCT, with a reported sensitivity of 63–79% [1,2]. Examination protocol is key to the diagnosis. An unenhanced scan is not routinely necessary, because it very rarely contributes to lesion detection [3]. For detection and characterization a bi-phasic (later arterial and venous phases) contrast-enhanced protocol is recommended, after IV administration of 0.5–0.6 g of iodine per kg b.w. (= 1.7–2 ml contrast material @ 300 mg/ml) at a flow rate of 4–5 ml/s. With current modulation techniques, lowering of kVp (80 or 100 kVp instead of 120 kVp) is possible of patients at lower body weight. This allows further reduction of contrast material. Reconstruction of thin, overlapping slices of 2.5-3 mm for viewing is superior to a slice thickness of 5 mm or more. In case of equivocal MDCT (e. g., in patients with moderate to severe liver steatosis) [4,5] or in patients with colo-rectal cancer and potentially resectable liver metastases, MRI should be performed.

The standard MRI protocol includes single breath-hold T1w GRE in- and opposed-phase, diffusion-weighted imaging (DWI), T2w TSE and contrast-enhanced pulse sequences. In clinical practice, MRI with liver-specific contrast agent (gadoxetic acid, Bayer Healthcare, Germany) is not only superior to contrast-enhanced MDCT, but also to MRI with non-specific gadolinium chelates, although is there is very little evidence in the literature. A recent meta-analysis showed that MRI with DWI and liver-specific contrast agent combined is superior to DWI or contrast-enhanced MRI alone (sensitivity 96% vs. 91% and 87%, respectively) [6].

Contrast-enhanced MDCT is used to monitor therapy response or disease progression. According to RECIST 1.1, uni-dimensional measurement of a maximum of 2 target lesions per organ (and a total of up to 5 lesions) defines tumour response. To eliminate inaccurate tumour measurements due to volume averaging, target lesions must have a minimum size of 1 cm. Complete response (CR) is defined by complete tumour disappearance, partial response (PR) by ≥30% decrease of the sum of all the longest diameters of all target lesions in comparison to baseline. Progressive disease (PD) is defined by an increase of 20% of longest diameters of target lesions in comparison to nadir, with stable disease (SD) representing tumour response not falling into one of the 2 categories. If patients with previously considered non-resectable metastatic disease turn potentially resectable due to overwhelming chemotherapy response, then CE-MRI should be performed to localise residual metastases for further planning [7].

In summary, MDCT examination protocol should be optimized for detection and characterization of focal liver lesions, considered the radiation dose in patient populations undergoing repeated scanning. MDCT is the standard technique for assessment of liver metastases and for follow-up after chemotherapy. Contrast-enhanced MRI including DWI and liver-specific agents is the modality of choice in patients with equivocal MDCT or in patients with CRC and potentially resectable liver metastases.

**References**

1. Kim YK, Park G, Kim CS, Yu HC, Han YM: Diagnostic efficacy of gadoxetic acid-enhanced MRI for the detection and characterisation of liver metastases: comparison with multidetector-row CT. Br J Radiol 2012;85:539-547.

2. Muhi A, Ichikawa T, Motosugi U, et al.: Diagnosis of colorectal hepatic metastases: comparison of contrast-enhanced CT, contrast-enhanced US, superparamagnetic iron oxide-enhanced MRI, and gadoxetic acid-enhanced MRI. J Magn Reson Imaging 2011;34:326-335.

3. Sadigh G, Applegate KE, Baumgarten DA: Comparative accuracy of intravenous contrast-enhanced CT versus noncontrast CT plus intravenous contrast-enhanced CT in the detection and characterization of patients with hypervascular liver metastases: a critically appraised topic. Acad Radiol 2014;21:113-125.

4. Kulemann V, Schima W, Tamandl D, et al.: Preoperative detection of colorectal liver metastases in fatty liver: MDCT or MRI? Eur J Radiol 2011;79:e1-6.

5. Berger-Kulemann V, Schima W, Baroud S, et al.: Gadoxetic acid-enhanced 3.0 T MR imaging versus multidetector-row CT in the detection of colorectal metastases in fatty liver using intraoperative ultrasound and histopathology as a standard of reference. Eur J Surg Oncol 2012;38:670-676.

6. Vilgrain V, Esvan M, Ronot M, Caumont-Prim A, Aubé C, Chatellier G: A meta-analysis of diffusion-weighted and gadoxetic acid-enhanced MR imaging for the detection of liver metastases. Eur Radiol 2016;26:4595-4615

7. Kuhlmann K, van Hilst J, Fisher S, Poston G: Management of disappearing colorectal liver metastases. Eur J Surg Oncol 2016;42:1798-1805.

#### O54 Opportunities and Challenges of PET/MR in metastatic disease

##### Hersh Chandarana (Hersh.Chandarana@nyumc.org)

###### Department of Radiology, New York University School of Medicine, New York, NY 10016, USA

Positron Emission Tomography and Compute Tomography (PET/CT) is routinely used in evaluation for cancer staging and assessment of metastatic disease. In addition, MRI is used as a problem solving tool. Until recently PET and MR examinations have been performed by separate PET and MR devices with temporal delay between these two types of acquisitions. However, recent introduction of hybrid PET/MR systems has made it possible to acquire PET and MRI data either simultaneously or near simultaneously.

PET/MR is a powerful tool that provides opportunity for multiparametric PET and MR imaging. This has tremendous potential in cancer staging, detection of metastatic disease, and assessment of treatment response. However, to take advantage of this powerful tool and implement it in clinical practice requires understanding of various components of the system, what the system can and can’t do, and how to optimize protocols to answer clinically relevant questions.

Some of these areas of opportunities and challenges include:

(1) PET/MR: System and workflow considerations such as

- Attenuation correction with MRI

- Validation of PET/MR with respect to PET/CT

- Whole body and targeted organ protocol

(2) Current and potential clinical indications for oncologic imaging such as

- Lymphoma Imaging

- Simultaneous locodistant staging for cancers

- Assessment of treatment response

(3) Limitations of PET/MR such as

- Lung imaging

- Osseous lesions

**References**

1. Parikh N, Friedman KP, Shah SN, Chandarana H. Practical guide for implementing hybrid PET/MR clinical service: lessons learned from our experience. Abdom Imaging. 2015 Aug;40(6):1366-73.

2. Delso G, Fürst S, Jakoby B, et al. Performance measurements of the Siemens mMR integrated whole-body PET/MR scanner. J Nucl Med. 2011 Dec;52(12):1914-22.

3. Paulus DH, Quick HH, Geppert C,et al. Whole-Body PET/MR Imaging: Quantitative Evaluation of a Novel Model-Based MR Attenuation Correction Method Including Bone. J Nucl Med. 2015 Jul;56(7):1061-6.

4. Martinez-Möller A, Eiber M, Nekolla SG, et al. Workflow and scan protocol considerations for integrated whole-body PET/MRI in oncology. J Nucl Med. 2012 Sep;53(9):1415-26.

5. Chandarana H, Heacock L, Rakheja R, et al. Pulmonary nodules in patients with primary malignancy: comparison of hybrid PET/MR and PET/CT imaging. Radiology. 2013 Sep;268(3):874-81.

6. Rakheja R, Chandarana H, DeMello L, et al. Correlation between standardized uptake value and apparent diffusion coefficient of neoplastic lesions evaluated with whole-body simultaneous hybrid PET/MRI. AJR Am J Roentgenol. 2013 Nov;201(5):1115-9.

7. Brandmaier P, Purz S, Bremicker K, et al. Simultaneous [18F]FDG-PET/MRI: Correlation of Apparent Diffusion Coefficient (ADC) and Standardized Uptake Value (SUV) in Primary and Recurrent Cervical Cancer. PLoS One. 2015 Nov 9;10(11).

8. Karan B, Pourbagher A, Torun N. Diffusion-weighted imaging and (18) F-fluorodeoxyglucose positron emission tomography/computed tomography in breast cancer: Correlation of the apparent diffusion coefficient and maximum standardized uptake values with prognostic factors. J Magn Reson Imaging. 2016 Jun;43(6):1434-44.

### 11.00 - 12.30 Paediatric Tumours

#### O55 MRI in fetal tumours

##### Gabriele Masselli, Gianfranco Gualdi

###### Radiology Department, Umberto I Hospital, Sapienza University Rome, Rome, Italy

####### **Correspondence:** Gabriele Masselli (gabriele.masselli@uniroma1.it)

Our ability to diagnose fetal tumours has improved over recent years. Although rare, the majority of fetal tumours can be detected by antenatal US.

Magnetic resonance imaging (MRI), due to its capabilities, can greatly improve the diagnostic performance of US during pregnancy, and it can provide an accurate evaluation of brain and body fetal tumours.

Congenital brain tumours, those that are observed prenatally or before the second month of life, are extremely rare.

Teratomas are reported to account for half of all congenital tumours. Other tumours listed in decreasing frequency include astrocytomas, lipomas, choroid plexus papillomas, and primitive

neuroectodermal tumours [1]. The most common presenting finding regardless of tumour type is macrocephaly.

Hydrocephalus, polyhydramnios, and hydrops are also commonly seen. Unlike the pediatric population, intracranial fetal tumours are most often supratentorial.

It can be difficult to distinguish the intracranial tumours prenatally. Teratomas often have a complex cystic and solid appearance and sometimes contain fat or calcification. A T2* sequence can be helpful in identifying associated hemorrhage, a finding most commonly seen in astrocytomas [2]. Choroid plexus papillomas are lobular intraventricular masses and can overproduce CSF resulting in hydrocephalus.

Midline intracranial lipomas are benign tumours associated with agenesis of the corpus callosum and will depict signal characteristics of fat (hyperintense signal on T1 and T2).

Excepting choroid plexus papillomas and lipomas, the prognosis of an intracranial tumour is poor, with tumours identified earlier in gestation having even worse outcomes [3].

Hemangiomas arise from abnormal cellular proliferation of vascular endothelial cells and are the most common vascular tumour in infancy. They affect approximately 10% of infants with a high female predilection, and greater than 50% of hemangiomas involve the head and neck region [4].

Congenital hemangiomas (CHs) are rare tumours which grow in utero and typically stabilize in size in the third trimester, Congenital hemangiomas may be visualized as early as 12 weeks’ gestation and typically involve the posterolateral neck.

They appear as well-defined heterogeneous solid masses with intermediate T1 signal and hyperintense T2 signal intensity.

Mediastinal teratomas are rarely seen in utero [5]. They are usually seen as an anterior mediastinal complex mass of mixed heterogeneity with or without internal calcifications (better seen by ultrasound) [5]. The mass can cause polyhydramnios secondary to esophageal compression, hydrops due to impaired venous return and fetal demise. After delivery, respiratory distress can occur.

There are many abdominal and pelvic cystic lesions or masses that can be detected prenatally, especially during the third trimester. Some originate in solid organs (liver, spleen, adrenal glands, kidneys) and are easily identified, but others such as enteric duplication cysts, mesenteric cysts, choledochal cysts, urachal cysts, or ovarian cysts may be difficult to differentiate. T1-weighted sequences can be useful for differentiating these anomalies from dilated intestinal loops and meconium pseudocysts.

Fetal brain tumours are very rare and their diagnosis during the prenatal period is challenging.

**References**

1. Alford R, Bailey A, and Twickler DM. Fetal Central Nervous System. In MRI of Fetal and maternal Diseases in pregnancy. 1nd edition. Edited by Masselli G, Switzerland Springer 2016; 91-118.

2. Cavalheiro S, Moron AF, Hisaba W, Dastoli P, Silva NS: Fetal brain tumours. Childs Nerv Syst. 2003;19:529–536.

3. Cassart M, Bosson N, Garel C, Eurin D, Avni F: Fetal intracranial tumours: a review of 27 cases. Eur Radiol. 2008;18:2060–2066.

4. Severino M, Schwartz ES, Thurnher MM, Rydland J, Nikas I, Rossi A: Congenital tumours of the central nervous system. Neuroradiology. 2010;52:531–548.

5. Avni F, Massez A, Cassart M: Tumours of the fetal body: a review. Pediatr Radiol 2009 39:1147–1157 DOI 10.1007/s00247-009-1160-6

#### O56 Tricky bone tumours

##### Anne MJB Smets (a.m.smets@amc.uva.nl)

###### Paediatric Radiologist, Department of Radiology, Academic Medical Center, University of Amsterdam, Amsterdam, The Netherlands

Although imaging characteristics of a bone lesion- a malignant tumour, a benign tumour or a tumour-like process- can be very specific, they may at times also be misleading and make characterization and differentiation more challenging or even impossible [1, 2]. Paediatric malignant bone tumours are rare. In children and adolescents Ewing sarcoma and osteosarcoma are the most frequent primary malignant bone tumours with a variable incidence according to age. Other malignant bone tumours such as primary skeletal lymphoma, chondrosarcoma, fibrosarcoma, hemangioendothelioma and adamantinoma are even rarer [3]. In young children bone metastases are generally caused by neuroblastoma and leukemia. The most common benign conditions that may show aggressive features and mimic paediatric bone tumours on imaging studies are Langerhans cell histiocytosis (LCH) and osteomyelitis, the latter particularly when the child has no fever [2, 4]. LCH results from an abnormal proliferation of histiocytes which produce prostaglandins causing resorption of medullary bone, mainly in the flat bones, spine and proximal long bones.

The plain radiograph is the initial and most useful examination for differentiating benign from malignant bone processes. CT and MRI are of diagnostic help in selected cases.

The age of the patient should always be taken into account: the paediatric bone has specific features (bone marrow conversion, variation of vascularization of the bone with age), and certain bone lesions are age-related [5]. A thorough analysis of the images, including the number of lesions, the location, the appearance and size of the lesion(s), and the appearance of the adjacent bone and periosteal reaction, is essential to direct the differential diagnosis.

In case of multiple lesions, Langerhans cell histiocytosis, chronic recurrent multifocal osteomyelitis and polyostotic fibrous dysplasia are possible diagnoses.

The type of bone where the lesion is located, flat, short or long, should be considered as well as the part of the bone involved: epi-, meta- or diaphysis.

The size of the lesion is not a very specific feature although lesions larger than 5-6 cm are suspicious for malignancy. The morphology of the lesion is equally non-specific: most benign lesions are elliptical but some malignant tumours can have the same appearance (lymphoma, low-grade osteosarcoma).

Benign lesions usually grow at a slow pace and have sharp borders. Malignant lesions commonly have poorly defined margins and show cortical destruction.

A periosteal reaction occurs whenever an infection or a tumour, either malignant or benign, irritates the periosteum or as a reaction to trauma.

The pattern of periosteal reaction can be benign (as seen in benign lesions or trauma) or aggressive (as seen in malignancies, infections or Langerhans cell histiocytosis).

Benign bone tumours generally have well-defined and often sclerotic margins, show cortical expansion and may produce solid periosteal reaction. Malignant tumours usually have poorly defined margins, show cortical destruction, have associated periosteal reaction of the spiculated, onionskin or interrupted type and are most of the time accompanied by a soft tissue mass.

In this workshop a number of pitfalls of daily practice in paediatric imaging will be shown and discussed.

**References**

1. Kan JH (2008) Major pitfalls in musculoskeletal imaging-MRI. Pediatric radiology 38 Suppl 2:S251-255.

2. McCarville MB (2009) The child with bone pain: malignancies and mimickers. Cancer imaging : the official publication of the International Cancer Imaging Society 9 Spec No A:S115-121.

3. Wootton-Gorges SL (2009) MR imaging of primary bone tumours and tumour-like conditions in children. Magnetic resonance imaging clinics of North America 17:469-487, vi.

4. Khung S, Budzik JF, Amzallag-Bellenger E, et al (2013) Skeletal involvement in Langerhans cell histiocytosis. Insights into imaging 4:569-579.

5. Chan BY, Gill KG, Rebsamen SL, et al (2016) MR Imaging of Pediatric Bone Marrow. Radiographics : a review publication of the Radiological Society of North America, Inc 36:1911-1930.

#### O57 Paediatric Pancreatic Masses

##### Alexander J. Towbin (Alexander.Towbin@cchmc.org)

###### Department of Radiology, Cincinnati Children’s Hospital, Cincinnati, Ohio, USA

Pancreatic tumours are rare in children. While ductal adenocarcinoma is the most common pancreatic neoplasm in adults, it is rarely encountered in children [1]. Compared to adults, the pancreatic tumours encountered in children are more often benign and, in general, have a better overall prognosis [1]. Tumours can be classified either by their cell type (epithelial versus nonepithelial) or their functional status (endocrine, exocrine, or cystic) [1,2].

While these classification systems have been described, they are rarely helpful in the imaging work-up as the cell type or cell function cannot be distinguished via anatomic imaging modalities. Instead, the tumour is usually differentiated based on imaging features, laboratory values, and patient demographic information. The purpose of this lecture is to describe the more common pediatric pancreatic masses and identify demographic, imaging, or laboratory characteristics that help to differentiate the different tumour types. During the course of the lecture, the following tumours will be discussed: pancreatoblastoma, solid pseudopapillary tumour, islet cell neoplasms, and pancreatic metastases. These tumours will be contrasted to tumour mimics such as pancreatic cysts, pancreatic cystosis, and pancreatic pseudocysts.

**References**

1. Nijs E, Callahan MJ, Taylor GA (2005) Disorders of the pediatric pancreas: imaging features. Pediatric radiology 35 (4):358-373; quiz 457. doi:10.1007/s00247-004-1326-1

2. Shet NS, Cole BL, Iyer RS (2014) Imaging of pediatric pancreatic neoplasms with radiologic-histopathologic correlation. AJR American journal of roentgenology 202 (6):1337-1348. doi:10.2214/AJR.13.11513

#### O58 Pitfalls in paediatric oncologic imaging

##### Kieran McHugh (kieran.mchugh@gosh.nhs.uk)

###### Radiology Department, Great Ormond Street Hospital for Children, London, WC1N 3JH, UK

Radiologists are very good at opting to image new masses in children with ultrasound. Many frequently forget, however, to assess for regional lymphadenopathy, which is particularly important with limb tumours (the inguinal or axillary regions for lower and upper limb tumours respectively should also be routinely evaluated). Paediatric tumours tend not to invade other organs but they can often be adherent to adjacent viscera – real time dynamic ultrasound can be very useful to assess movement of one organ relative to another. When considering cross-sectional imaging, CT is easier than MRI to do in children as CT scanning is so fast. MRI, however, is often the better test and is superior for assessing spinal canal invasion, chest wall involvement by tumour and bone marrow disease. MRI is best for pelvic, liver, paraspinal and neck masses as a general rule, and ideally should be performed for all new abdominal masses at initial presentation. Due to their usual lack of mediastinal and intra-abdominal fat, non-contrast enhanced CT is generally a waste of time and best avoided in children. Dual or triple phased enhanced CT is seldom necessary (all masses should have been assessed with Doppler ultrasound before a CT) and should also be avoided to reduce the radiation burden from CT.

### 14:00 - 15:30 Kidney and Adrenal

#### O59 Multiparametric Imaging of Renal Tumours

##### Hersh Chandarana (Hersh.Chandarana@nyumc.org)

###### Department of Radiology, New York University School of Medicine, New York, NY 10016, USA

Incidental detection of renal mass results in management dilemma. Historically all enhancing renal tumours without imaging evidence of bulk fat were considered surgical. However, it is clear that many of these small renal masses are either benign such as angiomyolipoma (AML) or oncocytoma, or are neoplasms with indolent behavior [1]. Surgical resection of these benign or indolent tumours, especially in patients with decreased renal function or other co-morbidities, results in increased cost without improvement in survival or mortality [2]. Use of advance imaging, such as diffusion weighted imaging (DWI) and perfusion weighted imaging (PWI), to non-invasively investigate renal tumour histopathology and aggressiveness can impact treatment decision and lower treatment cost.

Number of key observations highlighting the role of MR including advance imaging techniques in evaluation of renal masses is as listed below:

**1. Differentiating benign renal masses from malignant tumours.**

- Certain MRI features such as homogenous T2 signal, uniform enhancement, restricted diffusion with low ADC, and without evidence for necrosis and calcification can differentiate lipid poor AML from clear cell and papillary subtype of kidney cancers [3, 4].

- It is nearly impossible to discriminate benign oncocytoma from chromophobe and clear cell subtypes of kidney cancers on conventional imaging [5]. However, DWI and PWI have shown some promise in small studies.

**2. Tumour aggressiveness of solid RCC**

- Kidney cancers with different histologic subtypes differ in aggressiveness. Conventional MR imaging has shown some promise in differentiating papillary subtype of RCC from other subtypes based on hypovascularity, homogenous low T2 signal, T1 hyperintensity, and low ADC values. Advance DWI and PWI may further improve accuracy of MRI in discriminating papillary subtype from other types of kidney cancers.

- Clear cell subtype of kidney cancers is hypervascular with heterogeneous T2 and diffusion signal [6].

**3. Tumour aggressiveness/outcome of cystic RCC**

**-** Cystic RCC with less than 25% solid enhancing component tend to be less aggressive than solid RCC [7].

**References**

1. Thompson RH, Kurta JM, Kaag M, et al. Tumour size is associated with malignant potential in renal cell carcinoma. J Urol 2009;181(5):2033–6.

2. Huang WC, Levey AS, Serio AM, et al. Chronic kidney disease after nephrectomy in patients with renal cortical tumours: A retrospective cohort study. *The Lancet. Oncology*. 2006;7:735-740

3. Hindman N, Ngo L, Genega EM, et al. Angiomyolipoma with minimal fat: can it be differentiated from clear cell renal cell carcinoma by using standard MR techniques? Radiology. 2012 Nov;265(2):468-77.

4. Sasiwimonphan K, Takahashi N, Leibovich BC, Carter RE, Atwell TD, Kawashima A. Small (<4 cm) renal mass: differentiation of angiomyolipoma without visible fat from renal cell carcinoma utilizing MR imaging. Radiology. 2012 Apr;263(1):160-8.

5. Rosenkrantz AB, Hindman N, Fitzgerald EF, Niver BE, Melamed J, Babb JS. MRI features of renal oncocytoma and chromophobe renal cell carcinoma. AJR Am J Roentgenol. 2010 Dec;195(6):W421-7.

6. Chandarana H, Rosenkrantz AB, Mussi TC, et al. Histogram analysis of whole-lesion enhancement in differentiating clear cell from papillary subtype of renal cell cancer. Radiology. 2012 Dec;265(3):790-8.

7. Doshi AM, Huang WC, Donin NM, Chandarana H. MRI features of renal cell carcinoma that predict favorable clinicopathologic outcomes. AJR Am J Roentgenol. 2015 Apr;204(4):798-803

#### O60 Linking the imaging features of renal disease and their impact on management strategies

##### Hebert Alberto Vargas (vargasah@mskcc.org)

###### Department of Radiology, Memorial Sloan Kettering Cancer Center. New York, NY, USA

Most renal tumours are currently detected incidentally on imaging exams performed for a non-urologic indication. At the same time, the contemporary approach to the management of renal masses has also evolved, with additional options to the classic nephrectomy approach including nephron sparing surgery, focal therapies such as cryoablation and the growing use of active surveillance for masses considered unlikely to ultimately result in significant morbidity or mortality. The role of imaging is also shifting from mere tumour detection to providing crucial information required to tailor the management strategy to individual patients. In this session, we will discuss the key imaging findings that need to be evaluated in patients with renal tumours, and how they can be used to triage patients and guide treatments.

#### O61 Imaging workup and management of adrenal incidentalomas

##### Khaled M. Elsayes (KMElsayes@mdanderson.org)

###### Department of Diagnostic Radiology, The University of Texas MD Anderson Cancer Center, Houston, Texas, USA

Adrenal incidentalomas (AIs) are those adrenal masses, 1cm and larger typically discovered during imaging studies performed for unrelated complaints, excluding malignancy work-up. AIs are a common imaging finding with a prevalence of 4% [1], and increasing frequency with patient age. Current recommendations and guidelines for the management and follow-up of incidentally discovered adrenal masses fall into the broad categories of imaging guidelines and clinical guidelines. The goal of the imaging recommendations is to separate benign and malignant masses, while the primary concern of the clinical guidelines is to determine the biochemical activity and diagnostic characterization of AIs. This lecture will provide an overview of the recommendations from the American College of Radiology (ACR) [2], as well as the National Institute of Health (NIH) [3], the American Association of Clinical Endocrinologists (AACE), the American Association of Endocrine Surgeons (AAES), European Society of Endocrinology (ESE) [4], and European Network for the Study of Adrenal Tumours (ENSAT). Typical imaging features and pitfalls will be also illustrated.

**References**

1. Bovio S, Cataldi A, Reimondo G, et al. Prevalence of adrenal incidentaloma in a contemporary computerized tomography series. J Endocrinol Invest 2006; 29:298-302

2. Berland LL, Silverman SG, Gore RM, et al. Managing incidental findings on abdominal CT: white paper of the ACR incidental findings committee. J Am Coll Radiol 2010; 7:754-773

3. NIH state-of-the-science statement on management of the clinically inapparent adrenal mass ("incidentaloma"). NIH Consens State Sci Statements 2002; 19:1-25

4. Fassnacht M, Arlt W, Bancos I, et al. Management of adrenal incidentalomas: European Society of Endocrinology Clinical Practice Guideline in collaboration with the European Network for the Study of Adrenal Tumours. Eur J Endocrinol 2016; 175:G1-G34

### 14:00 - 15:30 Contrast-Enhanced Ultrasound

#### O62 Contrast enhanced ultrasound of the liver

##### Olivier Lucidarme (olivier.lucidarme@psl.ap–hop–paris.fr)

###### Service d'imagerie polyvalente, Groupe hospitalier Pitié–Salpêtrière, Paris, France

Contrast enhanced ultrasound (CEUS) imaging provides additional interesting features compared to MRI or CT and has become a major technique to characterize focal liver lesions. Mostly in non-cirrhotic liver but also in case of chronic liver disease as underlined by the recent release of the CEUS LIRADS in 2016. Thus, where usually CT or MRI offers a limited number of frames to assess the enhancement pattern of a focal liver lesion, US imaging may provide real time imaging with up to 15 to 25 frames per second as long as needed. The kinetic of the contrast arrival into the lesion can be seen directly without extrapolations. Microbubbles are blood pool agents. In contrary to Iodine and Gadolinium they do not leak outside the vascular space. Consequently the enhancement during the equilibrium and late phases are expected to be different from CT and MR. It is particularly true with malignant lesions where a clear washout is almost constantly seen while very rarely in benign lesions. The timing and the intensity of the washout is particularly important in case of chronic liver disease because differential diagnosis between hepatocarcinomas and intrahepatic cholangiocarcinomas is based on this. Our presentation will present the European guidelines concerning CEUS of the liver and the 2016 CEUS LIRADS, illustrated with several examples where CEUS was used as problem solver in front of incompletely characterized focal liver lesions.

#### O63 Contrast enhanced ultrasound in pediatric oncology

##### Beth McCarville (Beth.mccarville@stjude.org)

###### Department of Diagnostic Imaging, St. Jude Children’s Research Hospital, Memphis, TN, USA

Contrast enhanced ultrasound (CEUS) is a safe, radiation and sedation free modality that is child friendly, portable and cost effective. With the recent USA FDA approval of an ultrasound contrast agent for pediatric liver imaging, interest in this modality in children is rapidly growing. At our large children’s cancer hospital we use CEUS as a problem solving tool in a variety of conditions. We have also investigated the potential role of quantitative CEUS to assess tumour response to therapy in children with solid malignancies. In this lecture I will present a variety of clinical scenarios where CEUS has been helpful and will briefly demonstrate the potential value of quantitative CEUS in pediatric clinical cancer trials.

## Scientific Presentations

### Monday 2^nd^ October 12.30 – 13.00

#### S1 Texture analysis (TA) in the determination of complete tumour response in rectal cancer MRI

##### Horvat N., Veeraraghavan H., Khan M., Blazic I., Gollub M.J., Petkovska I.

###### Memorial Sloan Kettering Cancer Center, New York, NY, USA

####### **Correspondence:** Horvat N (natallymhorvat@gmail.com)

**Aim**: To investigate whether MRI TA on T2WI can distinguish residual tumour (RT) from complete response (CR) in rectal cancer after neoadjuvant chemoradiotherapy.

**Methods**: The IRB approved this HIPAA-compliant retrospective study and waived the requirement for patients’ informed consent. One hundred fourteen patients with rectal adenocarcinoma who underwent neoadjuvant chemoradiotherapy followed by surgery and restaging MRI at our institution were included (03/2012 to 02/2016). Two radiologists reviewed the cases and reached consensus on the post-treatment tumour bed location. ITK-SNAP software was used to manually segment the entire tumour region(s) on the high-resolution axial oblique T2-weighted images (WI). Haralick texture features (energy, entropy, correlation, contrast, homogeneity), and Gabor (Gab) edge images at angles 0, 45, 90 and 135 were computed and tested for significance comparing patients with CR *versus* RT on surgical pathology.

**Results**: Ninety-three patients (82%) presented RT on pathological examination of the surgical specimens and 21 (18%) presented CR. Synthetic minority oversampling technique (SMOTE) was used to augment the data, and random forest (RF) classifier was trained using five-fold cross validation to balance the number of partial and complete responders. The following texture features were most relevant by the RF classifier: Gab0 energy, Gab45 energy, Entropy, Gab45 entropy, Gab90 homogeneity, Gab0 contrast, Gab135, Contrast and Homogeneity. The area under the curve differentiating RT from CR was 0.89.

**Conclusion**: Combined texture features may predict RT from CR in treated rectal cancer. This unique radiomic assessment may be of incremental value for patients contemplating non-operative management of rectal cancer.

#### S2 Malnutrition and sarcopenia: risk factors for shorter survival after pelvic exenteration for recurrent gynecological malignancy

##### Seebacher V^1,2^, Rockall A^1^, Nobbenhuis M^1^, Sohaib A^1^, Knogler T^3^, Alvarez-Lopez R^1^, Kolomainen D^1^, Shaw C^4^, Shepherd J^1^, Barton DP^1^

###### ^1^Gynaecological Oncology Unit, The Royal Marsden NHS Foundation Trust, London, United Kingdom; ^2^Department for Gynaecology and Gynaecologic Oncology, Medical University of Vienna, Austria; ^3^Department for Radiology and Nuclear Medicine, Medical University of Vienna, Austria; ^4^Department of Nutrition and Dietetics, The Royal Marsden NHS Foundation Trust, London, UK

####### **Correspondence:** Seebacher V (veronika.seebacher@meduniwien.ac.at)

**Aim:** Pelvic exenteration is a highly morbid procedure performed as the last option for cure in selected patients with recurrent or persistent gynaecological malignancies. Due to a lack of objective factors predicting outcome, patient selection is in part based on subjective criteria. The aim of the present study was to investigate the prognostic value of malnutrition and sarcopenia on the outcome of patients with recurrent gynaecological malignancies treated by pelvic exenteration.

**Material & Methods:** We retrospectively evaluated muscle body composite measurements based on pre-operative CT scans, pre-operatively filled out questionnaires stratifying the risk for malnutrition, and clinical-pathological parameters in 65 consecutive patients with recurrent gynaecological malignancies treated by pelvic exenteration. Selected parameters were investigated for their predictive value for postoperative morbidity by logistic regression analyses. Relevant parameters were included in uni- and multivariate survival analyses.

**Results:** In 32 and in 34 patients pre-operative CT scans and questionnaires were available for analyses, respectively. We found (1) low muscle attenuation (MA) – an established factor for muscle depletion – and (2) malnutrition, based on a pre-operative questionnaire, to be independently associated with shorter overall survival (p = 0.006 and p = 0.008, respectively). Interestingly, MA was significantly lower in overweight and obese patients (p = 0.04). We did not find any of the investigated factors to be predictive for post-operative morbidity.

**Conclusion:** The present study suggests that pre-operative low MA and malnutrition, based on CT scan and questionnaire, are associated with shorter survival in patients with recurrent gynaecological malignancies treated with pelvic exenteration. Further studies are needed to validate these findings in larger cohorts.

#### S3 Comparison of 18F-FDG PET/MRI and MRI for whole-body staging of patients with primary cervical cancer

##### T. Sarabhai, B. Schaarschmidt, A. Wetter, J. Kirchner, B. Aktas, V. Ruhlmann, M. Forsting, K. Herrmann, L. Umutlu, J. Grueneisen

###### University Hospital Essen, Radiology, Essen, Germany

####### **Correspondence:** T. Sarabhai (theresia-catharina.sarabhai@uk-essen.de)

**Aim**: To assess and compare the diagnostic potential of integrated 18F-FDG PET/MRI and MRI alone for whole-body tumour staging of cervical cancer patients.

**Material and Methods:** A total 53 patients with histopathologically confirmed cervical cancer were prospectively enrolled and underwent an integrated whole-body 18F-FDG PET/MR examination prior to the initiation of definitive treatment. A radiologist and nuclear medicine physician performed a dedicated TNM-staging for each patient in accordance with the 7th edition of the AJCC staging manual. Therefore, MRI alone data were analyzed, followed by the interpretation of PET/MRI datasets. Finally, in a simulated interdisciplinary tumour board, all cases of discrepant staging results in MRI and PET/MRI were discussed and the most appropriate treatment strategy was determined.

**Results:** PET/MRI and MRI allowed for a correct determination of the T-stage in 45/53 (85%) and 46/53 (87%) cases, respectively. For the detection of nodal positive patients, the sensitivity, specificity and accuracy of PET/MRI were 83%, 90% and 87%, respectively. The respective values for MRI alone were 71%, 83% and 77%. Furthermore, PET/MR showed higher values for the detection of distant metastases than MRI alone (sensitivity: 87% vs. 67%, specificity: 92% vs. 90%, diagnostic accuracy: 91% vs. 83%). Among the patients with discrepant staging results, PET/MRI enabled correct treatment recommendations for a higher number (n = 9) of patients than MRI alone (n = 3).

**Conclusion:** The present results demonstrate the successful application of integrated PET/MR imaging for whole-body tumour staging of cervical cancer patients, enabling improved treatment planning when compared to MRI alone.

#### S4 Whole-body diffusion-weighted imaging and iron deposits in Hodgkin, follicular and diffuse large B-Cell lymphomas

##### Rahmouni A^1^, Cottereau AS^2^, Mule S^1^ , Lin Chieh^3^ , Belhadj K^4^, Vignaud A^5^, Copie-Bergman C^6,7^, Zerbib P^1^, Haioun C^4^, Luciani A^1^, Itti E^2^

###### ^1^Medical Imaging Department, AP-HP, Groupe Henri Mondor Albert Chenevier, CHU Henri Mondor, 51 Avenue du Marechal de Lattre de Tassigny, 94010 Creteil, France ; Université Paris Est Créteil, Paris, France; ^2^Nuclear Medicine Department, AP-HP, Groupe Henri Mondor Albert Chenevier, CHU Henri Mondor, 51 Avenue du Marechal de Lattre de Tassigny, 94010 Creteil, France ; Université Paris Est Créteil, Paris, France; ^3^Nuclear Medicine Department and Molecular Imaging Center, Chang Gung Memorial Hospital, Gueishan, Taiwan; ^4^Lymphoid malignancies Unit, AP-HP, Groupe Henri Mondor Albert Chenevier, CHU Henri Mondor, 51 Avenue du Marechal de Lattre de Tassigny, 94010 Creteil, France; Université Paris Est Créteil, Paris, France; ^5^Neurospin, CEA, Saclay, France; ^6^Pathology Department, AP-HP, Groupe Henri Mondor Albert Chenevier, CHU Henri Mondor, 51 Avenue du Marechal de Lattre de Tassigny, 94010 Creteil, France; ^7^Faculty of Medicine, Université Paris Est Créteil, Paris, France

####### **Correspondence:** Rahmouni A (alain.rahmouni@hmn.aphp.fr)

**Purpose:** To report and analyze the distribution of iron deposits within lymphoma lesions and to compare this distribution with FDG uptake and biological markers of inflammation.

**Materials and Methods:** We enrolled 61 untreated patients with a bulky diffuse large B-cell lymphoma (DLBCL), Hodgkin lymphoma (HL) or follicular lymphoma (FL) in an ongoing whole-body diffusion-weighted (DW) magnetic resonance (MR) imaging-based prospective study. If focal low signal intensity was detected on DW images, a T2 gradient echo sequence was systematically performed. Focal iron deposits were compared with biological inflammatory parameters (Mann-Whitney test/Fisher test) and with baseline FDG-PET/CT parameters (FDG uptake, SUVmax, total metabolic tumour volume TMTV).

**Results:** Focal iron deposits were found in 18 patients, mostly with aggressive lymphoma (9/26 HL, 8/20 DLBCL vs 1/15 FL, *p* = 0.047) and in patients with advanced stage disease (*n* = 15/18). Iron deposits in the spleen (*n* = 14), liver (*n* = 3) and nodal (*n* = 8) lesions corresponded to foci FDG uptake with mean SUVmax values of 9.8, 6.7 and 16.2, respectively. Patients with iron deposits had significantly higher serum levels of CRP, alpha1-globulin, alpha2-globulin and more frequently had microcytic anemia than those without such deposits (*p* = 0.0072, *p* = 0.003, *p* = 0.0068, *p* < 0.0001, respectively). They also had a significantly higher TMTV (*p* = 0.0055) and higher levels of spleen involvement (*p* < 0.0001). HL and DLBCL patients with iron deposits were less likely to have a complete metabolic response at two cycles on PET/CT.

**Conclusion:** Focal iron deposits are frequently detected in lymphoma lesions on DW MRI; this imaging biomarker is significantly associated with a higher inflammatory syndrome.

#### S5 Assessing early treatment response in Hodgkin lymphoma based on texture analysis using whole-body diffusion-weighted imaging

##### KN De Paepe; I Vieira, F De Keyzer,, P Wolter, O Bechter, D Dierickx, A Janssens, R Oyen, G Verhoef, M Koole, V Vandecaveye

###### University Hospitals Leuven, Leuven, Belgium

####### **Correspondence:** KN De Paepe (katjadepaepe@gmail.com)

**Aim:** To evaluate the role of whole body diffusion-weighted magnetic resonance scan (WB-DWI/MRI) texture analysis in treatment response assessment and treatment outcome prediction in Hodgkin lymphoma (HL) after one cycle of chemotherapy.

**Materials and methods:** Twenty-six patients with a new diagnosis of HL received a WB-DWI/MRI scan before treatment and after one cycle of chemotherapy (4weeks). For every lesion ADC first and second order histogram parameters and their interval change (∆ADCpar) were calculated. Per-lesion analysis consisted of Man-Whitney U (MW) tests of every ∆ADCpar and Monte Carlo (MC) permutation test to enhance statistical robustness. For every significant ∆ADCpar, a receiver operator characteristic curve (ROC), sensitivity (sens), specificity (spec), positive (PPV) and negative predictive value (NPV) were calculated. Per-patient analysis consisted of Kaplan Meier survival analysis with log rank test. Fluoro-deoxyglucose positron emission tomography/computed tomography (FDG-PET/CT) and follow-up served as reference of standard.

**Results:** Of 28 patients, 3 progressed and 2 had disease recurrence. First order parameters (∆ADCmean, ∆ADCmedian, ∆ADCskewness, ∆ADCkurtosis, ∆volume) weren’t significant, in contrast to second order parameters Energy (∆ADC_E_; p = 0.01 MW; p = 0.007 MC), Local homogeneity (∆ADC_LH_; p = 0.03 MW; p = 0.02 MC), and Entropy (∆ADC_H_; p = 0.003 MW; p = 0.007 MC). Sens was 67%, 67%, 89%; spec was 96%, 91%, 85%; PPV was 75%, 60%, 53%; NPV was 91%, 93%, 98% for, ∆ADC_LH_ and ∆ADC_H_ , respectively. ∆ADC_E_ (p = 0.01)_,_ ∆ADC_LH_ (p = 0.03) and ∆ADC_H_ (p = 0.002) had predictive value.

**Conclusion:** WB-DWI/MRI ADC texture analysis after one cycle of chemotherapy has the potential to assess treatment response and predict treatment outcome.

#### S6 [68Ga]Pentixafor-PET/MRI for CXCR4 imaging of chronic lymphocytic leukemia

##### Leithner D, Haug A, Wadsak W, Jaeger U, Raderer M, Staber P, Weber M, Mayerhoefer ME

###### Medical University of Vienna, Vienna, Austria and University Hospital Frankfurt, Frankfurt, Germany

####### **Correspondence:** Leithner D (doris.leithner@gmail.com)

**AIM:** [68Ga]Pentixafor-PET has recently emerged as a non-invasive tool for imaging and quantification of CXCR4 expression in vivo. It was the goal of this prospective study to determine whether [68Ga]Pentixafor uptake in the bone marrow of patients with chronic lymphocytic leukemia (CLL) is higher than in other oncological patients without bone marrow infiltration.

**METHODS:** Thirteen patients with previously untreated, or therapy refractory, histologically proven CLL, ten controls with pancreatic adenocarcinoma, and ten controls with MALT lymphoma underwent [68Ga]Pentixafor-PET/MRI on an integrated, simultaneous PET/MRI system. Maximum and mean standardized [68Ga]Pentixafor uptake values (SUVmax, SUVmean) were measured in the pelvis, the lumbar vertebra L4, and for the visually “hottest” bone marrow lesion (i.e., with the highest tracer uptake). Additionally, mean apparent diffusion coefficients (ADCmean) were measured in the pelvis on diffusion-weighted MRI. ANOVAs with post-hoc tests and ROC analyses were performed.

**RESULTS:** SUVmax and SUVmean differed significantly between CLL and pancreatic adenocarcinoma patients in the pelvis (P = 0.032 and P = 0.008) and L4 (P < 0.001 and P < 0.001). SUVmean also differed significantly in the pelvis (P = 0.020) and L4 (P = 0.041), and SUVmax in L4 (P = 0.019), between CLL and MALT lymphoma patients. An ROC analysis revealed the greatest areas-under-the-curve for separation of CLL from the two control groups for the SUVmax of the hottest lesion (0.94), and the SUVmax of L4 (0.92). There was no significant correlation between the SUVs and ADCmean in the pelvis.

**CONCLUSION:** [68Ga]Pentixafor uptake on PET/MRI is higher in the bone marrow of CLL patients than in other oncological patients without bone marrow infiltration.

### Tuesday 3^rd^ October 12.30 – 13.00

#### S7 Intravoxel incoherent motion diffusion imaging in stage III multiple myeloma before therapy and correlation with histology

##### Ravikanth Balaji (ravikanthbalaji@gmail.com)

###### Apollo Cancer Institute, Delhi, India

**Purpose:** To assess use of Intravoxel incoherent motion diffusion imaging in stage III multiple myeloma before therapy and correlation with histology.

**Methods and Materials:** 36 patients with biopsy proven stage III myeloma were included in the study. All patients underwent whole body diffusion imaging using DWIBS (Diffusion Weighted whole body Imaging with Background Signal Suppression) sequence on 1.5 Tesla Philips Achieva scanner. The larger lesions (> 1cm) in the vertebrae and pelvic bones were selected for IVIM study. Images were acquired in an axial plane with 11 b values - 0, 20, 30, 50, 80, 100, 200, 400, 80, 1000 and 1200 s/mm^2^.

The IVIM parameters (perfusion fraction [*f*], molecular diffusion coefficient [*D*], and perfusion-related *D* [D*]) and apparent diffusion coefficient (ADC) were obtained for both focal lesions and bone marrow. The molecular diffusion coefficient and ADC values were correlated with degree of plasma cell infiltration.

**Results**

Diffuse marrow involvement and focal lesions show high molecular diffusion D values with low ADC values and high f values at baseline.

Higher D values and significantly lower ADC values were obtained in focal lesions where plasma cell infiltrate was more than 50%.

**Conclusion:** The study found a significant correlation between plasma cell percentage in bone marrow histology and imaging parameters “apparent diffusion coefficient” and the diffusion coefficient *D* and degree of angiogenesis based on f values. Non-invasive acquired imaging parameters correlated with the degree of plasma cell infiltration and can also prove as important bioimaging markers for assessing response to therapy.

#### S8 STIR versus SPAIR fat suppression in WB-DWI/MRI: image quality and artefacts

##### R. Dresen, S Gailly, A Verbeurgt, V Vandecaveye

###### Department of Radiology, Leuven Cancer Institute, University Hospitals Leuven, KU Leuven, Leuven, Belgium

####### **Correspondence:** R. Dresen (elleke.dresen@uzleuven.be)

**Aim:** To compare the degree of artefacts and homogeneity between STIR and SPAIR diffusion-weighted MRI in patients undergoing a whole body diffusion-weighted (WB-DWI) MRI for detection of tumour recurrence.

**Methods:** Twenty patients with a history of digestive or ovarian cancer and clinical suspicion of tumour recurrence underwent a WB-DWI/MRI on a 3T scanner (Ingenia, Philips Healthcare, Best, The Netherlands). Diffusion-weighted images (b0 and b1000) were performed with both STIR and SPAIR fat suppression techniques for every patient. The degree of fat shift artefacts, susceptibility artefacts, distortion and homogeneity on both STIR-DWI and SPAIR-DWI was evaluated by two reviewers with a 5-point scale (ranging from severe artefacts with non-diagnostic image quality to no artefacts with excellent image quality). Interobserver agreement was calculated with Kappa statistics. Another reviewer evaluated both DWI techniques for lesion detection. Comparison of artefacts and lesion detection between STIR and SPAIR-DWI was performed with the non-parametric Wilcoxon matched-pairs signed ranks test. Differences were significant with a p < 0.05.

**Results:** The interobserver agreement was higher for STIR-DWI (kappa values: 0.68-0.75) than for SPAIR-DWI (kappa values: 0.56-0.73). STIR-DWI had significantly less fat-shift artefacts (Z = -3.54), susceptibility artefacts (Z = -3.50), distortion (Z = -3.58) and more homogeneous images (Z = -3.64) than SPAIR-DWI (p < 0.001 for all). Lesion detection was significantly better on STIR-DWI than on SPAIR-DWI (p < 0.001).

**Conclusion:** STIR-DWI provides superior lesion detection because of less artefacts and improved image quality, with better interobserver agreement. STIR-DWI offers a more homogeneous fat suppression than SPAIR-DWI. It should be the fat suppression technique of choice in WB-DWI/MRI.

#### S9 Whole-body diffusion-weighted MRI after one cycle of immunochemotherapy predicts treatment outcome in aggressive non-Hodgkin lymphoma.

##### KN De Paepe, F De Keyzer, O Gheysens, C Van Keerberghen, O Bechter, P Wolter, D Dierickx, A Jansenss, G Verhoef, R Oyen, V Vandecaveye

###### University Hospitals Leuven, Leuven, Belgium

####### **Correspondence:** KN De Paepe (katjadepaepe@gmail.com)

**Aim**: To evaluate the predictive value of whole body diffusion-weighted magnetic resonance imaging (WB-DWI/MRI) after 1 cycle of immunochemotherapy (ICT) in aggressive NHL.

**Materials and Methods:** 45 patients with aggressive NHL were consecutively enrolled and had a WB-DWI/MRI with 2 b-values (b = 0-1000 s/mm^2^) before and after 1cycle ICT; 42/45 had an interim 18F- fluorodeoxyglucose-positron emission tomography/computed tomography (FDG-PET/CT )and 43/45 end-of-treatment FDG-PET/CT. Apparent diffusion coefficient (ADC) and b1000 histograms were constructed per lesion and the difference of derived histogram parameters (mean, median, standard deviation, skewness, kurtosis) between baseline and interim scan (∆ADCpar and ∆b1000par) were calculated. Per lesion Man-Whitney U tests identified significant discriminating ∆ADCpar and ∆b1000par per tissue type (lymphoid, bone, extranodal). Patients were stratified as good and poor responders based on the least responding lesion. Predictive value of FDG-PET/CT and WB-DWI/MRI were assessed using Kaplan Meier survival analysis and multivariate Cox regression with disease-free-survival (DFS) as outcome.

**Results**: At a cut-off follow-up time of 48 months, 32 patients showed complete remission, 6 recurrent and 7 progressive disease. Patients with a lymphoid lesion attaining a ∆ADCmean < 0 or bony lesion with a ∆b1000mean of less than 16% or extranodal lesion demonstrating a ∆b1000kurtosis < 0 were designated as poor responders. WB-DWI/MRI accurately predicted outcome in 44/45 (98%;p < 0.001), interim PET/CT in 30/42 (70%,p = 0.006) and end-of-treatment FDG-PET/CT in 35/45 (78%,p = 0.003). Multivariate analysis identified WB-DWI/MRI as single independent prognostic factor (p < 0.001).

**Conclusion:** WB-DWI/MRI is highly predictive of treatment outcome in aggressive NHL after only one cycle of immunochemotherapy.

#### S10 18F-FDG-PET detects complete response to anti-PD1-therapy in melanoma patients two weeks after start of therapy

##### C. Pfannenberg, F. Seith, A. Forschner, Holger Schmidt, B.Gückel, K.Nikolaou, C. la Fougère, C.Garbe and N. Schwenzer

###### Department of Radiology, Department of Nuclear Medicine and Clinical Molecular Imaging and Department of Dermatology, Eberhard Karls University, Tuebingen, Germany

####### **Correspondence:** C. Pfannenberg (christina.pfannenberg@med.uni-tuebingen.de)

**Purpose**: Aim of the study was to evaluate if 18F-FDG-PET has the potential to detect complete responders to anti-PD1-therapy in patients with stage IV melanoma already two weeks after start of therapy.

**Methods**: Between September 2014 and May 2016, 10 patients with unresectable metastasized melanoma (4 females; 65 ± 12 y) received a whole-body 18F-FDG-PET/MRI examination at three time points: before start of therapy (t0, baseline), two weeks (t1, study examination) and three months after start of therapy (t2, reference standard). Therapy response was assessed with PET response criteria in solid tumours (PERCIST). Time to progression and overall survival (OS) were obtained for all patients. This subgroup analysis is part of a prospectively conducted ongoing study.

**Results**: Three patients with partial metabolic response in PET at t1 turned out to have complete response at t2. No tumour relapse was observed in those patients so far (observation period: 265, 511 and 728 days, respectively). At t2, progressive metabolic disease (PMD) was seen in six patients from whom four showed PMD and two showed stable metabolic disease (SMD) at t1. OS in patients with PMD at t2 varied between 148 and 814 days. SMD at both t1 and t2 was seen in one patient, tumour progress was observed after 308 days.

**Conclusion**: Our study indicates that 18F-FDG-PET might be able to identify complete responders to PD1-therapy as early as two weeks after start of therapy initiation in stage IV melanoma patients. This might help to shorten therapy regimes and avoid unnecessary side effects in the future.

#### S11 Metastasis in locally-advanced and recurrent breast cancer: Is there any correlation with breast cancer subtype?

##### Pinto K., Bansal J.

###### Cardiff & Vale University Health Board, Cardiff, Wales

####### **Correspondence:** Pinto K (kevinpinto5@gmail.com)

**Aim**: To enhance clinical understanding of molecular subtypes of breast cancer and their specific patterns of metastasis, which would aid in guiding future imaging surveillance with the objective of detecting metastases and relapses at an early, more treatable, stage.

**Methods**: A retrospective analysis of patients with breast cancer (n = 201) between March 2006 and December 2016 was performed. All patients had a staging CT scan of Chest, abdomen and pelvis. Tumours were classified based on Oestrogen (ER) and Human Epidermal receptor (HER2) status. Review of recurrence data, both local and systemic, along with metastasis to site-specific regions, including bone, lung, liver, brain, mediastinum/pleura and extra-axillary lymph nodes, was undertaken.

**Results**: The most significant associations with distant metastases were between HER2-positivity and the liver (p = 0.02), along with ER/HER2 negativity (Triple negative, TN) associated with extra-axillary nodal and mediastinal/pleural metastases (p < 0.001). ER-positivity had a strong association with bone metastases, but this was not statistically significant (p = 0.44). TN cancers presented most aggressively and had a noticeable correlation with brain metastases (p = 0.041). Non-ER-positive cancers had the greatest incidence of systemic recurrence, of which TN cancers had the highest proportion of metastases (62.2%). Time till recurrence was smallest for TN tumours (3.1 years) versus 6.85 years for ER positive cancers.

**Conclusion**: The different molecular subtypes have marked differences in patterns of metastatic spread. HER2-positivity is significantly associated with liver metastasis and TN cancers with brain and extra-axillary/mediastinal and pleural metastases.

#### S12 MR Radiomics features of brain metastases support primary tumour identification

##### H. Kniep, F. Madesta, T. Schneider, T. Gauer, R. Werner, S. Siemonsen

###### Universitätsklinikum Hamburg-Eppendorf, Hamburg, Germany

####### **Correspondence:** H. Kniep (h.kniep@uke.de)

**Aim:** Radiomics is a quantitative analysis approach for medical images allowing non-invasive tissue differentiation. Radiomics is regarded as one of the most promising tools for cancer diagnosis and treatment in radiology and radiotherapy. Patients with brain metastases as first manifestation of cancer represent up to 26% of all brain metastases patients. As targeted treatment options depend on the primary lesion type, radiological in vivo techniques for metastasis type identification are desirable to reduce biopsy interventions and to allow for faster and efficient diagnosis. The present study investigates the potential of primary tumour identification employing Radiomics-based image features of different brain metastases.

**Methods:** The analysis includes Radiomics features of 155 brain metastases from T1 post-contrast images of 59 untreated patients with breast cancer (n = 48 metastases), SCLC (n = 57 metastases) and melanoma (n = 50 metastases). Tumour segmentation was conducted semi-automatically by two senior professionals. Quantitative image features and basic clinical data (age, sex) were transferred into a random forest machine learning algorithm. 5-fold cross-validation was used for model validation.

**Results:** Importance based predictor selection yielded a subset of 19 features from a total of 51 processed features. ROC AUC classification performance was 0.84 for SCLC, 0.76 for melanoma and 0.88 for breast cancer. Performance increase due to Radiomics features vs. clinical data alone was +0.14 for SCLC, +0.10 for melanoma and +0.03 for breast cancer.

**Conclusion:** Combining quantitative image feature analysis with clinical data in a machine learning approach may accelerate primary tumour identification and could serve as supportive decision tool for treatment regimes.

## Poster Presentations

### P1 Is there any role for Image Guided lung and liver ablation as a method of non-curative cytoreduction?

#### Simon Smith, Paul Jennings, Zoltan Szcucs

##### Ipswich Hospital, Heath Road, Ipswich, Suffolk. UK

###### **Correspondence:** Simon Smith (simon.smith@ipswichhospital.nhs.uk)

**Learning Objectives:** Thermal ablation is an accepted technique in the management of both primary and secondary tumours in the lung and liver. Treatment is usually performed with curative intent with the aim of treating all visible disease. Increasingly radiologists are being asked to treat larger tumours or to target specific metastases in patients with multi-organ disease, or specific metastases which are enlarging in the context of other sites of quiescent disease (‘escape lesions’). Here the aim is not cure, but tumour debulking.

Although logical arguments have been put forward to support this technique (symptom relief, cytoreduction with potentially improved results of chemotherapy, increasing symptom-free or overall survival), there is limited published evidence to support this approach.

This poster will review the available literature and present a number of cases (with extensive imaging) where ablative tumour de-bulking has been used with variable benefit.

We will examine the potential benefits and the limitations of this technique and suggest some guidelines for future use which may be particularly helpful to sites starting an ablation service.

**Conclusion**: There is very limited evidence to support the use of thermal ablation as a means of limited tumour debulking. The demand for this technique is often patient driven and potential patients should be counselled appropriately. Local experience suggests there may be a small subset of patients who may benefit.

### P2 Cryoablation of pulmonary tumours: procedural and post-procedural imaging features

#### Lyons GR, Pua BB, Bassik N, Winokur RA, Talenfeld AD, Madoff DC

##### New York-Presbyterian Hospital and Weill Cornell Medicine, New York, NY, USA

###### **Correspondence:** Lyons GR (grl9017@nyp.org)

**Learning Objectives**

1. Show imaging characteristics of successful pulmonary tumour cryoablation and normal follow-up appearance.

2. Demonstrate complications such as pneumothorax, pulmonary hemorrhage, and local tumour recurrence.

**Content Organisation**

Percutaneous cryoablation is a viable treatment option for lung tumours in non-operative patients. As this modality is increasingly employed, clinicians will need familiarity with normal post-operative imaging characteristics and procedural complications. We review our experience with CT-guided cryoablation of 93 pulmonary tumours and average imaging follow-up of 1 year, including:

-Normal intra-procedural imaging, including selection of target tumours, optimized probe positioning, and post-ablation appearance.

-Procedural complications such as pneumothorax and hemorrhage, as well as treatment options for these complications

-Expected post-procedural appearance of ablated nodules, including a predictable pattern of size increase followed by exponential tapering of lesion size

-Imaging features of locally recurrent disease and options for subsequent re-intervention.

**Conclusion**

Percutaneous cryoablation of pulmonary nodules is an effective modality for treatment of select lung tumours. Interpretation of intra-procedural and follow-up imaging is essential for successful treatment and identification of complications.

### P3 Leiomyosarcoma of the ovarian vein: Computed Tomography features of a rare disease entity

#### KY Kwok, CYH Chan, KWS Ko, BCK Chow

##### Department of Radiology and Imaging, Queen Elizabeth Hospital, Hong Kong

###### **Correspondence:** KY Kwok (kwok_kai_yan@hotmail.com)

**Learning objectives**: To review the radiological features of ovarian vein leiomyosarcoma, primarily on CT scan. The crucial roles of CT scan in the diagnosis and management planning of ovarian vein leiomyosarcoma are emphasized.

**Content organisation:** Leiomyosarcoma of ovarian vein origin is extremely rare. Due to its infrequent occurrence, the diagnosis of ovarian vein leiomyosarcoma is difficult. Nevertheless, modern CT scan provides the corner stone to achieve the principle radiological diagnosis and is essential for the planning of patient management. In this poster presentation, we demonstrate a logical approach to derive the diagnosis. This is based on analyzing the anatomical origin, orientation, internal architecture and enhancement characteristics of the tumour. The importance of CT scan in tumour staging, complication detection and management decision, in particular how to facilitate surgical planning, are also discussed.

**Conclusions:** CT scan plays a critical role in the diagnosis and management planning of ovarian vein leiomyosarcoma. A logical approach in analyzing the CT scan would facilitate the assessment of this rare malignancy.

### P4 Incidental pulmonary nodules: 2017 Fleischner society guidelines

#### SJ Neville, LE Quint

##### Michigan Medicine, University of Michigan, Ann Arbor, MI, USA

###### **Correspondence:** SJ Neville (sjnevil@umich.edu)

**Learning Objectives:** To review the 2017 Fleischner Society guidelines for the management of small, incidental pulmonary nodules, and to apply the guidelines to illustrative cases.

**Content Organisation**: Incidental lung nodules are commonly seen on chest CT scans. The Fleischner Society has consolidated and revised previous recommendations for the management of incidental solid and subsolid nodules based on new data. Recognizing the high prevalence of benign nodules, the guidelines now recommend fewer and less frequent follow-up exams, and the minimum threshold nodule diameter for follow-up has been increased. Recommended scanning follow-up intervals are now given as ranges, rather than fixed intervals, acknowledging the heterogeneity of patient scenarios. A longer initial follow-up interval is now recommended for subsolid nodules, and the length of complete follow-up for these lesions has been extended to 5 years.

The guidelines specify the acquisition of thin section, low x-ray dose images. Nodules are measured using consistent techniques on serial exams with (1) the average of long and short axis diameters, rounded to the nearest mm, based on manual measurements, in whichever plane yields the largest dimensions, or (2) volumetry. Comparison should be made with the oldest available exam and more recent exams.

Examples of solitary and multiple, solid and subsolid nodules in high and low risk patients will be presented, along with management guidelines.

**Conclusion:** Familiarity with the new Fleischner Society guidelines for the management of small, incidental nodules is important in clinical practice, in order to determine the appropriate recommendations for follow-up imaging or intervention.

### P5 Combination of MR imaging and PET CT in diagnosis and management of pelvic lesions

#### De Luca S, Grana M F, Alarcón L, Casalini Vañek E, Fernández M, Eyheremendy E P

##### Deustches Hospital, Buenos Aires, Argentina

###### **Correspondence:** De Luca S (sdeluca@hospitalaleman.com)

**Learning objectives:** To review the spectrum of imaging findings and utility of combined high resolution MR, whole body 18 FDG and choline PET CT in pelvic lesions from different etiologies.

**Content organisation:** Cross sectional imaging techniques ultrasound (US), computed tomography (CT) and high resolution magnetic resonance imaging (MRI) have been used to detect and follow up patients with gynecologic and not gynecologic pelvic diseases. All these methods show anatomic details and morphologic changes within the pelvis.

Positron emission tomography (PET) fusion with CT, shows functional information with anatomic localization both within and outside the confines of the pelvis. FDG PET cannot be reliably used to determine the extent of local tumour invasion but definitely plays a role in the evaluation of lymph node and other distant metastases.

Multidisciplinary approaches that combine surgery, chemo- and radio-therapy are often employed and the imagings play a central role in treatment planning, to triage patients define its scope, and to evaluate the success of therapy.

Complementary roles of MR and integrated positron emission tomography – computed tomography (PET-CT) with 18F-FDG and Choline both at primary presentation and post-treatment are also described.

We will review imaging characteristics and analyze bibliographic reviews in

Uterine (cervical-endometrial) carcinoma

Vulvar and vaginal carcinoma

Rectal-Anal carcinoma

Prostatic carcinoma

Other rare masses (defining origin)

**Conclusions:** The combination of multi-parametric MRI and PET/CT should be ideal tools for patients with pelvic lesions.

In the future, wider application of integrated PET/MRI can be expected.

### P6 MRI of rectal carcinoma - What the surgeon wants to know?

#### Krishnan V, Chinchure D

##### Khoo Teck Puat Hospital, Singapore, Singapore

###### **Correspondence:** Krishnan V (dr_vijayrad@yahoo.co.in)

**Learning objectives:** To discuss the role of MRI in the preoperative evaluation of rectal carcinoma using recent advanced imaging sequences with illustrations.

To highlight the key areas of involvement which the surgeon wants to know in deciding appropriate management

**Content organisation:** The main aspects of MR in assessing rectal tumour are stage, depth of invasion and relationship of tumour with adjacent structures.

We will review the role of MRI in pre-operative imaging of rectal tumour with following aspects:

- Current MRI acquisition protocol with advanced imaging sequences.

- MR anatomy of rectum and related structures.

- Localisation of tumour

- Assessment of depth of tumour

- Assessment of extension of tumour and relationship with adjacent structures including mesorectal fascia, pelvic side wall and anal sphincter.

- Assessment of nodal status

This poster also aims to explain the importance of MR imaging in deciding appropriate clinical management and its prognostic implications. By assessing the local extent of tumour MR is helpful in deciding the proper management, surgery versus combined chemotherapy - radiotherapy. Assessment of circumferential resection margin is an important prognostic factor predicting the future tumour recurrence.

**Conclusions:** MR rectum imaging is done to identify the local extent of involvement, which, not only guides the surgeon in deciding appropriate management but also has prognostic implications.

### P7 Implementing a volunteer sonographer led hospice based ultrasound service – the first three years’ experience

#### J. Eastman, C. Midgley

##### Saint Francis Hospice, Havering-Atte-Bower, UK

###### **Correspondence:** J. Eastman (joeastman@sfh.org.uk)

**Learning objectives**: This poster aims to present our experience in establishing a sonographer-led ultrasound service in a Hospice setting.

**Content organisation**: Ultrasound imaging is a valuable resource which has been widely used in hospitals for many years. Its usage has been to slow to develop in hospices despite improvements to the cost of machines and their accessibility. Noting the absence of ultrasound facilities during previous experiences with the local hospice, the first author approached the Medical Director (second author) to discuss developing a volunteer on-site ultrasound service, in consultation with BMUS Ultrasound Clinical Governance Guidelines (October 2008).

This poster will present a review of the experience of establishing the provision of ultrasound scanning in a hospice setting. Over the course of three years 212 scans were carried out on 184 patients. Indications for scans included suspected urinary retention, DVT, and assessment of abdominal ascites or pleural fluid. A wide range of pathologies were found, from disease progression to gallstones accounting for pain. Establishing guidelines to ensure safe practice, to equip staff with confidence to request a scan and to inform staff when a scan would be helpful has taken time. The experience of the authors demonstrates that such challenges can be overcome in this environment.

**Conclusion:** The authors have been able to demonstrate that a hospice based ultrasound service is achievable, effective and safe. Clinicians value the greater certainty available with the use of sonography when signs and symptoms are subtle or complex, while patients appreciate not having to travel for imaging.

### P8 Imaging features of Glomus Jugulare tumour with histopathological correlation

#### Shankar.KR, Varsha.T, Hazif.B, Sindhu.N, Parthasarathi.A, Pravin.GU

##### Rajarajeswari Medical College and Hospital, Bangalore, India

###### **Correspondence:** Shankar.KR (shankar.1991@gmail.com)

**Learning objectives:** To review the spectrum of imaging findings in a case of glomus jugulare tumour with histopathological correlation.

**Content organisation:** Glomus jugulare tumours are tumours of the temporal bone in the skull, jugular foramen to be precise. The exact etiology is unknown. Patients may present with a wide range of symptoms which include hearing loss, dizziness, dysphagia, pain, facial nerve palsy and so on. These tumours originate from the chief cells of the paraganglia or glomus bodies, located within the wall (adventitia) of the jugular bulb. Their reported incidence is 1 case per 1.3 million people with moderate female predilection.

We will review the imaging findings in a twenty five year old female patient who presented with symptoms of facial nerve palsy on left side.

We will discuss plain Computed Tomography (CT) and contrast Magnestic Resonance Imaging (MRI) findings including

• Its location

• Infiltration into adjacent structures.

• Density on CT

• Intensity on different MRI sequences.

• Post contrast changes.

Histopathological finding and post-operative CT images will also be reviewed.

**Conclusion:** Glomus jugulare tumours are rare, slow-growing, hyper vascular tumours that arise within the jugular foramen of the temporal bone. A combination of CT scanning and contrast MRI is the imaging regimen of choice for glomus jugulare tumours.

### P9 Disease and treatment related complications in Oncologic imaging with 18F-FDG PET/CT

#### Raghava Kashyap, Kanhaiyalal Agrawal

##### Mahatma Gandhi Cancer Hospital and Research Institute, Andhra Pradesh, India

###### **Correspondence:** Raghava Kashyap (kashyapkr@gmail.com)

**Learning objectives**: To review the imaging changes in disease related and treatment related complications in oncologic practice demonstrated on 18F FDG PET CT

**Content organisation:** 18F-fluoro deoxy glucose (FDG) positron emission tomography (PET) is routinely performed imaging modality in the oncology patients. It is not unusual to have incidental but clinically important findings on FDG PET/CT study. These findings may be due to complication of radiotherapy, chemotherapy, intervention or primary tumour itself. These incidental findings should be recognized on PET/CT.

The data of PET/CT scans from our institute from 2014 September till current was reviewed for complications. The images are categorized into disease related and treatment related changes and discussed below.

Disease related complications that are presented here include

1. Mass effect with mid line shift in brain

2. Neural compression

3. Aspiration

4. Broncho-pleural fistula

5. Pulmonary embolism

6. Malignant Trachea-esophageal fistula

7. SVC obstruction

8. Small bowel obstruction

9. Hydronephrosis

10. Varicocele secondary to tumour thrombosis

11. Malignant vesico-vaginal fistula

Treatment related complications predominantly of radiotherapy that are presented are

1. Radiation pneumonitis

2. Radiation hepatitis

3. Hemorrhagic cystitis

4. Urethral stenosis and myositis

5. Stress fractures induced by radiation

6. Wedge compression fractures of vertebra secondary to radiation

7. Osteoradionecrosis

**Conclusion**: Complications of disease or treatment are frequently encountered on imaging in oncologic practice. Some of these complications could be life threatening and may need urgent intervention. The interpreting physician should be aware of the findings and convey them to the treating physicians for prompt necessary action.

### P10 Infrequent primary and secondary pancreatic tumours

#### Nazar M, De Luca S, Carrera C, Barale C., Castro Cavallo L and Eyheremendy E. P.

##### Deustches Hospital, Buenos Aires, Argentina

###### **Correspondence:** Nazar M (mnazar@hospitalaleman)

**Learning objectives:** Describe MRI and CT findings of infrequent primary and secondary pancreatic tumours.

Discuss the differential diagnosis of these rare pancreatic tumours.

**Content organization:** Magnetic resonance imaging (MRI) and Computed tomography (CT) have been used to detect and follow patients with pancreatic tumours.

Among the primary malignancies pancreatic adenocarcinoma are the most common. In contrast, neuroencondrine tumours, solid pseudopapillary tumours, medullary tumours and lymphoma are quite rare.

Secondary malignancies of the pancreas are divided based on the mode of spread: hematogenous and direct invasion by adjacent organ.

The most commonly reported primary tumours metastatic to the pancreas are: lung, gastrointestinal tract, kidney, breast and melanoma.

**Conclusions:** It is important that the radiologist be familiar with these infrequent tumours in order to initiate the appropriate lesion-specific work-up and treatment and avoid unnecessary procedures, including surgery.

### P11 Thoracic manifestations of breast cancer, its treatment and complications – what the radiologist needs to know

#### S Bhuva, J Lee

##### Oxford University Hospitals NHS Foundation Trust, Oxford, UK

###### **Correspondence:** S Bhuva (shaheel.bhuva@gmail.com)

**Learning Objectives:** To recognise the thoracic manifestations of breast cancer and of its treatment, including:

• Thoracic emergencies

• Nodal disease

• Pulmonary, mediastinal and pleural metastatic disease

• Early and late complications, including post-operative and post-radiation therapy findings

Content organisation: Thoracic imaging is vital to the management of patients with breast cancer. Our local practice includes a dedicated metastatic breast multidisciplinary team meeting.

We offer a pictorial review of the essential thoracic features relating to breast cancer and its treatment.

1. Thoracic emergencies

• Pulmonary embolus with right heart strain

• Cardiac tamponade due to pericardial metastasis or following radiotherapy to sternal metastasis.

• Metastatic thoracic cord compression

2. Nodal disease:

• locoregional axillary relapse adjacent to, and partially masked by, postsurgical change in the axilla.

• PET-CT scan demonstrated FDG avid malignant internal mammary lymph node, confirmed by US-guided fine needle aspiration.

• axillary nodal relapse, contralateral to the original breast cancer, demonstrating 'cross over' low resistant lymphatic spread.

• sarcoid-like reaction in mediastinal nodes – a benign mimic of malignant disease

3. Pulmonary, mediastinal and pleural metastatic disease

• pulmonary metastasis - from solid to cavitation

• lymphangitis carcinomatosis

• mediastinal relapse with resultant phrenic nerve palsy and hemidiaphragmic paralysis

• pleural metastasis

4. Early and late complications:

• post radiotherapy pneumonitis to fibrosis

• sarcoma in the supraclavicular fossa of a patient treated with radiotherapy for breast cancer decades earlier

**Conclusion:** Knowledge of the thoracic manifestations of breast cancer, its treatment and its complications will allow accurate interpretation and contribute to the effective multidisciplinary care of patients with breast cancer.

### P12 Understanding ESTRO– radiological pearls and pitfalls for delineating target volumes for radiotherapy in breast cancer

#### S Bhuva, J Lee, F Duane, C Taylor, G Andrade

##### Oxford University Hospital NHS Foundation Trust, Oxford, UK

###### **Correspondence:** S Bhuva (Shaheel.Bhuva@gmail.com)

**Learning objectives:** To present the essential radiological components entailed in the ESTRO guidance, enabling radiologists to aid radiation oncologists with precise radiotherapy planning in breast cancer.

**Content organisation:** Adjuvant radiotherapy in early stage breast cancer is proven to reduce the risk of recurrence and improves survival outcomes, but exposes the heart, lungs and soft tissue to radiation related side-effects.

Technological advances include a transition in radiotherapy planning from using 2D planar bony landmarks to a 3D CT-based delineation of nodal target volumes using vascular anatomy to localise regional nodal levels.

We offer a practical, easy to follow, and illustrated guide to applying the ESTRO consensus guidelines (2016) on delineating the regional lymph nodes for adjuvant irradiation in early stage breast cancer.

We will:

1. Clarify the lexicon regarding 3D localisation.

2. Highlight the challenges posed by RT positioning on an unenhanced CT scan.

3. describe, illustrate and identify the essential anatomical landmarks that underpin the regional nodal levels, including: the subclavian vein, arch of the subclavian artery, internal carotid artery, differences in internal mammary/thoracic arterial origins, medial perforating mammarian and lateral thoracic arteries; musculature (scalenus anterior and pectoralis major and minor); and bones (clavicle, lateral margin of the first rib, fourth rib and humeral head).

4. offer an approach to applying borders to each lymph node level based on the essential anatomical landmarks.

5. Illustrate relevant anatomical variants.

**Conclusion:** This practical guide will enhance understanding, and implementation, of the ESTRO guidance and promote collaboration between radiologists and radiation oncologists to improve patient care.

### P13 Asymptomatic Nasopharyngeal carcinoma: Early detection and assessment of loco-regional extension with imaging

#### Krishnan V, Shenoy JS

##### Khoo Teck Puat Hospital, Singapore, Singapore

###### **Correspondence:** Krishnan V (dr_vijayrad@yahoo.co.in)

**Learning objectives**

- To discuss the role of imaging in early detection of nasopharyngeal carcinoma (NPC) in asymptomatic individuals with illustrations.

- To highlight the key areas that should be scrutinised to avoid missing the lesion and also helps in proper assessment of extension.

**Content organisation:** The aspects of imaging in NPC are early detection, local extent assessment and assessing the response to treatment on follow up studies, with MRI and CT of nasopharynx playing essential roles in assessment. Radiologists should also pay careful attention for post nasal space in all routine CT/MR brain scans to detect otherwise asymptomatic NPC especially in high prevalence areas.

We will review imaging of NPC with following aspects:

- Anatomy of nasopharyngeal region related to NPC.

- Importance of giving more attention to postnasal space in routine brain scans especially in patients of Asian descent

- Key areas helping the early detection of tumour.

- Key areas to scrutinise to assess the locoregional extension and perineural spread.

- Assessment of adjacent nodal status.

This poster also aims to explain the importance of early detection and its prognostic implications.

**Conclusions**

- Posterior nasal space / nasopharynx should be carefully scrutinised in asymptomatic individuals even in routine brain scans especially in patients of Asian descent.

- MRI and CT have complementary roles in assessing the locoregional extension.

- Awareness of routes of extensions of NPC, subtle radiological signs of disease helps in early detection, better prognosis and therapeutic outcome.

### P14 Brachial Plexopathy in breast cancer – what the radiologist needs to know

#### C Goss, E Green, S Bhuva

##### Oxford University Hospitals NHS Foundation Trust, Oxford, UK

###### **Correspondence:** C Goss (charlottevgoss@gmail.com)

Breast cancer commonly metastasises to the supraclavicular and cervical paravertebral regions. In addition, the standard treatment for breast cancer involves radiotherapy to the supraclavicular fossa. The brachial plexus (BP) is an intricate structure that extends through the paravertebral and supraclavicular regions. Its anatomical location renders it vulnerable to damage and dysfunction in breast cancer patients, either due to malignant infiltration or as a complication of radiotherapy. The BP is a challenging anatomical structure to image and interpret and, while brachial plexopathy is a relatively rare occurrence, the Radiologist needs to be able to differentiate between metastatic disease and other causes of pathology in the BP. We offer a pictorial review of the anatomy of the BP and the radiological presentation of brachial plexopathies.

The superior soft tissue contrast and multi-planar imaging offered by magnetic resonance imaging (MRI) enables the accurate assessment and characterisation of abnormalities of the BP, as well as delineation of the extent and integrity of adjacent structures. Our local MRI protocol includes multiplanar T1, T2, STIR, and post-contrast sequences.

We offer a pictorial review of the anatomy of the BP and discuss some of the varied pathology affecting the structure and surrounding tissues. We present cases from our metastatic breast cancer multi-disciplinary team meeting at a large tertiary Oncology centre, which illustrate the MRI features differentiating brachial plexopathies caused by tumour recurrence versus radiation-induced damage and highlight other causes of BP pathology that a Radiologist may encounter when imaging breast cancer patients.

### P15 Extra-nodal Lymphoma

#### N Parvizi, S Bhuva, C Johnson, N Taylor, M Subesinghe

##### Department of Clinical Radiology, Oxford University Hospitals NHS Trust, Oxford, UK

###### **Correspondence:** N Parvizi (nassim.parvizi@gmail.com)

**Learning objectives**: To review key imaging features that may prompt the radiologist to consider lymphoma in extra-nodal sites.

**Content Organisation:** Lymphoma is a multisystem malignant disease with heterogeneous clinico-radiological presentations whereby it can often mimic other disease processes. This poster will present a series of cases with appropriate differential diagnoses to highlight the key imaging features that may prompt the radiologist to consider lymphoma.

Our selection of cases will demonstrate cases arising in the:

1. Central nervous system

2. Head and Neck

3. Thorax

4. Gastrointestinal system

5. Genitourinary system

6. Endocrine systems

**Conclusion:** Extra-nodal lymphoma is a great mimic of multisystem malignant disease. It can present with a wide range of radiological presentations and is important to consider when the constellation of symptoms/signs and radiology do not fit a system-based cancer.

### P16 Various scintigraphic and anatomico-metabolic fusion imaging procedures in diagnosis, theranostics and follow-up of thyroid malignancies

#### CNB Harisankar (hari.cnb@gmail.com)

##### Meenakshi Mission Hospital and Research Centre, Madurai, India

**Learning objectives**: To review the well-established conventional scintigraphic techniques and newer applications of fusion imaging procedures in thyroid malignancies in the diagnosis, post-operative evaluation, theranostics and in follow up of thyroid malignancies.

**Content organisation:** Introduction of fusion imaging equipment like SPECT-CT and PET-CT, has allowed for better evaluation and characterisation of scintigraphic abnormalities. Several of these findings have important implications on prognostication and further management of these malignancies.

The different scintigraphic applications presented here are follows:

1. 99m- NaTco4 and hybrid SPECT-CT evaluation of thyroid nodule

2. Thyroid Incidentalomas in 18F-FDG PET-CT study

3. Pre-operative FDG PET-CT evaluation of thyroid malignancies - DTC, Thyroid lymphoma, anaplastic thyroid cancer

4. Typical iodine scintigraphic findings of remnant thyroid and lymph nodal metastases on whole body iodine imaging and hybrid SPECT-CT imaging

5. Typical findings of metastatic thyroid cancer - Lung / Bone metastases

6. De-differentiation of well-differentiated thyroid cancer during course of treatment - concept of thyroglobulin elevated negative iodine scinitgraphy - TENIS and its evaluation using 18F-FDG PET-CT

7. Bone scintigraphy and 18-Fluoride scintigraphy in metastatic thyroid cancer

**Conclusion:** Scintigraphic procedures play an important diagnostic and theranostic role in well-differentiated thyroid cancer. Fusion imaging like SPECT-CT and PET-CT add prognostic and diagnostic information available from scintigraphy and alter therapeutic decision making. 18-F FDG PET-CT has an important role in evaluation of de-differentiated thyroid cancer and helps plan subsequent therapy. Scintigraphic procedures and fusion imaging also have a role in rare thyroid pathologies like thyroid lymphoma and anaplastic thyroid cancer.

### P17 Atypical intramural corporal uterine lesion and peritoneal nodules: the added value of Proton MR Spectroscopy

#### Heming C.A.M., Leite J.A., Zuza D.C., Laterça F.J., Calabria M., Martins, C.L.C.S., Carvalho L.A., Sena B.F.D.P.Q., Scapulatempo H.H., Barbosa I.M.G.A. , Mora P.A.R., Scapulatempo-Neto C., Guitmann G., Jr Coutinho A.C.

##### Americas Medical City/ Americas Serviços Médicos, DASA, COI, Rio de Janeiro, Brazil

###### **Correspondence:** Heming C.A.M (hemingcarol@gmail.com)

**Learning objectives:** To review the spectrum of imaging findings of atypical leiomyomas and how Proton MR spectroscopy (PMRS) can aid in the diagnosis of a benign intramural nodule, even in the setting of associated peritoneal nodules. We will also review the imaging findings of disseminated peritoneal leiomyomatosis, a rare disease, and its mimics.

**Content organisation:** Atypical leiomyomas can be challenging in clinical practice and the main differential diagnosis is sarcoma, especially when there are associated peritoneal nodules, which concerns about malignancy. Proton MR spectroscopy provides metabolic information, which is useful when differentiating between benign and malignant tumours.

We present a case of a young nulliparous woman with no gynecological workup who presented with abdominal volume increase in the ER. Her pelvic MRI showed peritoneal nodules and atypical leiomyoma in the conventional sequences, but PMRS of the mural nodule showed findings of benignity. She went to surgery and intraoperatory freezing biopsy of the peritoneal nodules confirmed peritoneal leiomyomatosis, which, in conjunction with MR spectroscopy findings, left the surgical team comfortable to alter the plan from total abdominal hysterectomy to myomectomies, preserving the patient´s fertility.

We will review the imaging aspects of:

- typical and atypical leiomyomas and its differential diagnosis;

- the PMRS findings of benign and malignant uterine lesions;

- peritoneal leiomyomatosis and its mimics.

**Conclusions**: Proton MRS is a technique that aids in the differentiation between benign and malignant lesions also in the uterus and should be more frequently performed in the gynaecological radiology practice and radiologists should be familiar with its main findings.

### P18 Dual time point 18-FDG PET-CT in the assessment of pulmonary nodules

#### De Luca S, Carrera C, Alarcón L, Pascuzzi D, Wirtz M, Eyheremendy E P

##### Deustches Hospital, Buenos Aires, Argentina

###### **Correspondence:** De Luca S (sdeluca@hospitalaleman.com)

**Learning objectives:** To evaluate the usefulness of dual-time-point 18F-FDG PET CT for differentiating benign from malignant pulmonary nodules.

To review the usefulness of CT 18F-FDG PET in the detection and characterization of pulmonary nodules allowing an adequate differential diagnosis.

**Content organisation:** The differential diagnosis of a solitary pulmonary nodule is wide and includes benign causes as well as primary lung cancer and lung metastases.

18F-FDG PET is frequently used in characterizing solitary pulmonary nodule and is a useful technique.

FDG is not tumour-specific. There are numerous causes of 18 F-FDG uptake in benign processes and is a potential source of false-positive results. Otherwise, some malignant lesions might show only minimally increased activity.

In most 18 F-FDG PET studies, imaging is performed 50–60 min after 18 F-FDG injection; however, the uptake of 18 F-FDG in malignancies is expected to increase over 1.5–5 h. In theory, images obtained 2–3 h after 18 F-FDG injection should show improved contrast between tumour and normal tissues or benign processes, because uptake is increased in the tumour and decreased in the normal back-ground.

We retrospectively analysed 21 patients who had delayed images 18F-FDG PET in diagnosis of solitary pulmonary nodules, improving the accuracy in 85% of them.

**Conclusions:** Dual-time point imaging might improve the accuracy of FDG PET in the evaluation of lung nodules.

### P19 Imaging review of vaginal pathologies

#### Walker CM, Johnson CA

##### The John Radcliffe Hospital, Oxford, UK

###### **Correspondence:** Johnson CA (c.a.yoong@gmail.com)

**Learning objectives:** To be familiar with the appearances of vaginal tumours, both benign and malignant, to aid an accurate and timely diagnosis for those that require treatment and for those that do not.

**Content organisation**: Benign and malignant tumours of the vagina are rare. Neoplastic lesions that develop from other locations in the female genital tract may also spread to the vagina. Most vaginal tumours produce no symptoms until significant size is reached. Symptoms and signs may include a sensation of pressure, dyspareunia, obstruction of the vagina or urethra, and/or vaginal bleeding and discharge.

Many lesions will be detected during a routine examination in the asymptomatic patient or discovered as an incidental finding during imaging of another organ system. Imaging may include TV ultrasound, CT and MR scans.

Primary vaginal carcinomas are rare and typically found in the older female. This neoplasm arises solely from the vagina without involvement of the external os or vulva. Malignant involvement of the vagina from metastatic spread is much more common and usually a consequence of direct local invasion from the female urogenital tract. Other primary cancers include melanomas and sarcomas.

Benign tumours include vaginal leiomyomas, Gartner’s and Bartholin’s cysts and endometriotic lesions. Familiarity with the normal female anatomy is essential.

**Conclusion:** Benign and malignant tumours of the vagina are found in all age groups with malignant changes more typically seen in the older population. Typical imaging appearances are demonstrated. Secondary vaginal cancer is more common than primary.

### P20 Ovarian Lymphoma, an educational review

#### Johnson CA, Walker CM, Bhuva S, Subesinghe M, Taylor N

##### The John Radcliffe Hospital, Oxford University NHS Trust, Oxford, UK

###### **Correspondence:** Johnson CA (c.a.yoong@gmail.com)

**Learning objectives:** Malignant lymphoid tumours of the female genital tract are rare; however, the ovaries are the most common site involved, with young women being affected. It may occur as a primary extranodal site or in conjunction with other sites of nodal or extra nodal disease. This distinction is important as primary extranodal lymphomas are less aggressive with a 5 year survival of 80% as compared to 33%.

**Content organisation**: Ovarian lymphomas may be discovered incidentally during investigations for abdominal or pelvic complaints. Subtypes commonly encountered are diffuse large B cell, Burkitt’s and Follicular lymphomas. Ultrasound, CT and MR are important in assessing the ovaries and any spread of disease. Typical features of ovarian lymphoma include a hypoechoic pattern on USS, hypodense lesions with mild contrast enhancement on CT, and homogeneous masses with low T1 and raised T2 signal on MRI.

Primary extra nodal ovarian lymphoma has a better prognosis than secondary lymphoma. The diagnosis of primary ovarian lymphoma can only be made if there is presence of an ovarian mass confined to one or both ovaries, no evidence of lymphoma elsewhere, and disease free interval of 60 months after oophorectomy.

**Conclusion:** Ovarian involvement by lymphoma is less common than the typical nodal masses and splenomegaly but is found in 25% of women dying from lymphoma and accounts for 1.5% of primary ovarian tumours.

### P21 Multimodality approach for imaging upper tract urothelial carcinoma (UTUC)

#### A Mastan, S Suut

##### Salford Royal NHS Foundation Trust, Manchester, UK

###### **Correspondence:** A Mastan (aliya.mastan@doctors.org.uk)

**Learning objectives:** To investigate presence of UTUC using various forms of imaging, from ultrasound and IVU (intravenous urography) to more dedicated cross-sectional studies such as CT (computed tomography) and MR (magnetic resonance).

**Content organisation**: UTUC is a common malignancy affecting the genitourinary tract. This can be a difficult condition to diagnose because when patients present with haematuria, a flexible cystoscopy is usually performed and upper urinary tracts are not routinely visualised.

The presence of UTUC is therefore very reliant on imaging, aimed to guide urologists before a more invasive ureteroscopy is undertaken.

A multimodality approach is crucial due to the multicentric nature of UTUC . In fact, some carcinomas demonstrate very subtle radiological changes whereby they can even manifest as parenchymal infiltration in a kidney which can still maintain a normal reniform configuration.

Our pictorial review aims to highlight several interesting imaging findings of UTUC. Synchronous UTUC in known oesophageal carcinoma, recurrence of UTUC within an ileal conduit, parenchymal infiltration and filling defects seen on diagnostic ureteroscopies are among the cases which will be discussed.

Our review highlights the importance of diagnosing UTUC at an early stage to aid management and treatment.

**Conclusion:** A multimodality approach to imaging plays a key role in the diagnosis of UTUC. The multicentric nature and unusual as well as obscure imaging features of UTUC can make this condition difficult to diagnose. Therefore, recognition of all imaging manifestations should be recognised. In addition, early recognition of these conditions is key to treatment and better prognosis.

### P22 Tumoural pseudoangiomatous stromal hyperplasia (PASH): Radiological features of a rare entity

#### Khin YT, V Reynolds, TR Shimpi, HT Leung

##### Khoo Teck Puat Hospital, Singapore, Singapore

###### **Correspondence:** Khin YT (kythein@yahoo.com)

**Learning objectives:** To review the spectrum of imaging findings of tumoural pseudoangiomatous stromal hyperplasia (PASH), a histologically-proven rare benign breast lesion.

**Content organisation:** PASH is a benign hormone-related proliferation of breast stromal cells, commonly affecting premenopausal women or those on hormone replacement therapy. Histologically, it is characterized by slit-like pseudovascular spaces lined by endothelium-like spindle cells. The nodular or tumoural form of PASH is extremely rare. Tumoural PASH may presents as a palpable, well circumscribed solitary mass or may be multifocal. Despite its benign nature, it often grows over time and may also recur post excision, hence may need mastectomy. The mammographic findings of PASH vary from no identifiable abnormality to a circumscribed mass, the sonographic features from undetectable lesion to a well-defined focal mass with variable sonographic appearance. On sonoelastography, the strain ratio ranges between 2.5 to 5, stiffness equal to the surrounding tissue, indicative of benign aetiology. We also demonstrate through this pictoral review, a rare association of PASH with co-existing breast carcinoma. The imaging features of tumoural PASH are variable and hence may mimic other well defined masses, as fibroadenoma/ hamartoma or ill-defined masses such as breast carcinoma.

**Conclusion:** Tumoural PASH is a benign breast lesion that can have variable imaging features, rapid growth and post excision recurrence. It also has association with co-existing carcinoma and malignant transformation and mimics other benign and malignant breast lesions on imaging.

### P23 Lung adenocarcinoma: computed tomography features, revised pathology classification and prognosis prediction

#### ^1^Sandomenico F., ^2^Pizza S, ^2^Rusconi G, ^2^De Rosa G., ^1^Petrillo A

##### ^1^National Cancer Institute, IRCCS "G.Pascale Foundation", Department of Diagnostic Imaging, Naples, Italy; ^2^University of Naples Federico II, Department of Advanced Biomedic Science, Naples, Italy

###### **Correspondence:** Sandomenico F (f.sandomenico@virgilio.it)

**Learning objectives:** To review the spectrum of CT findings in lung adenocarcinoma in order to correlate them to the 2011 International Association for the Study of Lung Cancer/American Thoracic Society/European Respiratory Society (IASLC/ATS/ERS) Classification of lung cancer.

***Content*** **organisation** ***:*** We will review main CT features of lung adenocarcinoma and which of these are correlated to either a better or worse prognosis:

- solid, sub-solid or pure “ground glass” nodules (GGN)

- margins (smooth, lobular, spiculated)

- air bronchogram

- bubble-like areas

- concave notches

- pleural tag or retraction.

We will discuss 2011 IASLC/ATS/ERS classification of lung cancer (above all relatively to the adenocarcinoma) and its new terminology introduced to better reflect the heterogeneous group of cancer formerly known as bronchoalveolar cell carcinoma:

- adenocarcinoma in situ (AIS)

- minimally invasive adenocarcinomas (MIA)

- lepidic predominant adenocarcinoma (LPA)

- predominantly invasive non-mucinous adenocarcinoma (plus a lepidic component)

- invasive mucinous adenocarcinoma.

Fleischner Society guidelines for management of sub-solid pulmonary nodules are discussed.

Finally, main surgical managements are briefly discussed:

- wedge resection

- lobectomy

- pneumonectomy.

**Conclusions:** Radiologists must be aware of classification of lung adenocarcinoma and guidelines for management of GGNs as they play an important role in differentiating pre-invasive, minimally invasive and frankly invasive lesions. This could allow more accurate staging, treatment and prognosis prediction of these patients.

### P24 Evolution of PET/CT attenuation and artifacts & factors affecting SUV: corrective measures

#### Mythri Shankar, Mohan HK, Rajendra Kulkarni

##### Cytecare Cancer Hospitals, Bangalore, India

###### **Correspondence:** Mythri Shankar (dr.mythrishankar@gmail.com)

Technological developments have led to a newer modality of Hybrid Fusion Imaging – PETCT, which involves fusion of 2 separate image series (Positron Emission Tomography & Computerized Tomography) onto each other. Acquisition of good quality images and meaningful interpretation of the scans often requires a deeper understanding of the parameters involved which cause these artifacts. Artifacts may lead to a false positive or negative diagnosis, which may be caused by administration of contrast (Oral or IV), presence of metallic objects, Patient motion during image acquisition and truncation /mis-registration of the fused Images. It is also important to understand the concept of attenuation coefficient. PET images are corrected with CT attenuation, sometimes artifacts occur when attenuation correction (from CT) is used in the corrected PET image. PET photons (Monochromatic photons) always have the exact same energy. CT x-rays (polychromatic x-rays) vary in their energy beam by at least 1%. This results in an alteration of the attenuation coefficient and therefore in artifacts.

### P25 Normalised periprostatic fat volume at MRI predicts prostate cancer aggressiveness in patients undergoing radical prostatectomy

#### N. Dahran, M. Szewczyk-Bieda, C. Wei, G. Nabi, S. Vinnicombe

##### University of Jeddah, Jeddah, Saudi Arabia and University of Dundee, Dundee, UK

###### **Correspondence:** N. Dahran (n.dahran@dundee.ac.uk)

**Aim**: Periprostatic fat has been shown to influence prostate cancer behaviour through secretion of chemokines and growth factors. The aim of this study was to investigate the relationship between periprostatic fat volume (PFV), measured on pelvic MRI, and prostate cancer (PCa) grade (Gleason score; GS) and pathological staging (pT) following radical prostatectomy (RP).

**Materials and methods:** PFV was determined using a semi-automated segmentation technique on contiguous T_1_-weighted axial MRI slices from the level of the prostate base to the apex. PFV was normalised to prostate volume (PV) to account for variations in PV (NPFV = PFV/PV). Abdominal fat area (AFA) and subcutaneous fat thickness (SFT) were measured on T_1_-weighted axial slices at the level of the umbilicus and the upper border of the symphysis pubis, respectively. Patients were stratified into three risk groups according to post-operative GS: ≤6, 7(3 + 4), and ≥7(4 + 3).

**Results**: NPFV was significantly different between risk groups (*p* = 0.001) and positively correlated with post-operative GS (ρ = 0.294, *p* < 0.001). Men with stage pT_2_ had significantly lower NPFV (2.28 ± 0.98) than men with stage pT_3_ (2.76 ± 1.25), t(160) = -2.760, *p* = 0.006. There was a difference in NPFV between those with upgraded from GS 6 post prostatectomy (2.43 ± 0.98; *n* = 26) compared to those with no upgrade from GS 6 (1.99 ± 0.82; *n* = 17); however, this did not reach statistical significance (*p* = 0.11). There was no correlation between BMI, AFA and SFT and PCa aggressiveness.

**Conclusions**: NPFV measured at MRI is strongly correlated with aggressivity of PCa. Its role as a potential non-invasive imaging biomarker of PCa behaviour warrants exploration.

### P26 Essentially clinically occult brainstem strokes associated with resection of large cerebellopontine cistern vestibular schwannomas

#### D. Quint, T. Hollon, L. Savastano, D. Argersinger, B.G. Thompson

##### University of Michigan Medical Center, Ann Arbor, MI, USA

###### **Correspondence:** D. Quint (djquint@umich.edu)

**Aim**: Acute essentially clinically silent brainstem strokes have been demonstrated within weeks of surgical resection of large cerebellopontine angle (CPA) cistern vestibular schwannomas. In this study, we retrospectively reviewed a series of these patients who underwent post-operative MRI in an attempt to determine the incidence of such strokes. We also performed a cadaveric anatomic study to investigate the possible underlying pathophysiology of these strokes.

**Subjects & methods**: 258 patients underwent vestibular schwannoma resection surgery over an 11 year period at our institution. 54 of these patients underwent MRI during the 2 weeks immediately following surgery. Patient demographics, clinical history, tumour size, operative approach and MRI results were reviewed.

Four cadaveric dissections of CPA cisterns were performed with special attention to microvascular anatomy.

**Results**: 4 patients (7.4%) demonstrated acute ischemic changes on diffusion MRI in the region of the middle cerebellar peduncle. Two of these patients demonstrated post-operative nystagmus and ipsilateral dysmetria. Symptoms resolved in all 4 patients within 4 weeks of surgery. All 4 patients demonstrated chronic lacunar ischemic changes on follow-up MRI. Cadaveric dissections revealed that microvessels arising from the anterior inferior cerebellar artery supplying the cranial nerve 7-8 complex may have been injured during the vestibular schwannoma CPA surgery.

**Conclusion**: Microvascular brainstem ischemia is an under-recognized complication of CPA vestibular schwannoma surgery, but does not appear to result in long-term clinical sequelae.

### P27 Whole-Body MRI for cancer screening in asymptomatic subjects; frequency and management of relevant findings

#### G. Petralia^1^, A.R. Padhani^2^, E. Tagliabue^4^, P. Summers^1^, C. Giorgi^3^, E. Di Taranto^3^, E. Pace^3^, P. Pricolo^1^, F. Zugni^1^, M. Bellomi^1^

##### ^1^Istituto Europeo di Oncologia, Milan, Italy; ^2^Paul Strickland Scanner Centre, Northwood, UK; ^3^Advanced Screening Centres Italia, Sarnico, Italy; ^4^Clinical Trial Center, Scientific Directorate, Fondazione IRCCS Istituto Nazionale dei Tumori, Milan, Italy

###### **Correspondence:** F. Zugni (fabio.zugni@ieo.it)

**Aim:** To report the prevalence of incidental findings in asymptomatic subjects undergoing diffusion whole-body magnetic resonance imaging (DWB-MRI) for cancer screening.

**Methods:** We included all consecutive asymptomatic subjects who underwent DWB-MRI from January to April 2017. Individuals with cancer were excluded. All examinations were performed on a 1.5T MR (Magnetom Avanto, Siemens Healthineers, Germany), with the following protocol: axial images (T1, T2, diffusion weighted) from head to thigh, sagittal T1 and STIR of the spine. Findings were located in the following body regions (head, neck, chest, abdomen, pelvis, limbs, bone) and assigned a 5-point Likert score: 1 (normal), 2 (highly likely to be benign), 3 (likely benign requiring follow-up or further investigation), 4 (likely malignant requiring further investigation), 5 (highly likely to be malignant requiring biopsy and/or immediate referral).

**Results:** Out of the 222 subjects (84 F, 138 M; mean age 51.6 years) included, DWB-MRI was not performed in 2 due to body size (BMI > 38) and in 8 due to claustrophobia. In the 212 subjects analyzed, we found Likert 1 in 10 subjects (4.7%), Likert 2 in 179 (84.4%), Likert 3 in 20 (9.4%), Likert 4 in one (0.5%), Likert 5 in two (1.0%). Malignant cancer was found in two subjects (colon with liver metastases in one, bladder and kidney cancer in the other, respectively).

**Conclusions:** The prevalence of incidental findings was high, but in the majority no follow-up or further investigations were needed to confirm their likely benign nature. The diagnosis of cancer in two asymptomatic subjects allowed immediate referral.

### P28 Comparison of magnetic resonance imaging findings of primary adenocarcinoma and non-adenocarcinomatous malignant tumours of the rectum

#### N Niyamanon, K Hongsakul, T Tubtawee

##### Prince of Songkla University, Songkhla, Thailand

###### **Correspondence:** T Tubtawee (ttubtawee@gmail.com)

**Aim**: To compare the magnetic resonance imaging (MRI) findings in adenocarcinomas and non-adenomatous malignant tumours of the rectum, by focusing on morphology, basic MRI sequence signal changes and pattern enhancement.

**Materials and methods:** Retrospectively review of MRI studies of the rectum was done in pathologically proven rectal adenocarcinomas (n = 57) and non-adenomatous malignant tumours (n = 7); consisted of neuroendocrine tumours, gastrointestinal stromal tumours (GIST), malignant melanomas and leimyosarcoma. MR images were interpreted by two radiologists with 5 and 6 years of experience. All MRI studies were analyzed for MRI quality, border, T1W/T2W intensity, internal hemorrhage/calcification, cystic portion/necrosis, pattern of enhancement and extension. Fisher exact tests were used with significant *p*-value set at less than 0.05.

**Results**: Adenocarcinomas showed predominant mural thickening (98.2%) while non-adenomatous malignant tumours preferred focal intraluminal or exophytic locations (85.7%) with a statistically significant difference (*p* < 0.01). An irregular border was frequently observed in 82.5% of the adenocarcinomas while a smooth or lobulated border was present in all non-adenomatous malignant tumours (*p* < 0.01). Although hemorrhage/calcification and internal cystic portion were observed more frequently in non-adenomatous malignant tumours (42.9%, 42.9%) than adenocarcinomas (12.3%, 10.5%), no statistical significance was found (*p* = 0.07, 0.52).

**Conclusions**: Rectal mass with a mural thickening pattern and irregular border were likely to be typical adenocarcinomas. Intraluminal and exophytic lesions more correlate with non-adenomatous tumours.

### P29 Positive predictive value of prostatic multiparametric MRI in patients with high PSA levels: Nairobi experience

#### Mutala TM, Lazaro E, Odhiambo AO, Kimani NM

##### University of Nairobi, Nairobi, Kenya

###### **Correspondence:** Mutala TM (musilamutala@gmail.com)

**Aim:** To correlate positive mp- MRI findings with histological diagnosis in patients with high PSA levels**.**

**Methods:** A prospective study recruited 50 patients with raised PSA and suspicious lesions on rectal examination who underwent mp-MRI examination between May to November 2016. The examination was conducted using 1.5T Philips MRI machine. Sequences for study were T2W, diffusion weighted imaging (DWI) and dynamic contrast enhancement (DCE). Lesions were graded according to the prostate imaging and reporting and data system (PI-RADS). Histological diagnosis was made following targeted biopsy on lesions having PI-RADS 3, 4 and 5 characteristics. Two cut-off sets, one at PI-RADS 3 and another at PI-RADS 4 were compared for statistically significant difference on the positive predictive value (PPV).

**Results:** The mean age of the patients was 68.9 years (SD ± 10.7). The mean PSA level was 53.7 (± 67.7). The total number of PI-RADS 3, 4 or 5 lesions was 65. On histopathology, 59 of these lesions turned to be positive for prostatic carcinoma giving a positive predictive value of 90.7%. PI-RADS 3 (intermediate) lesions alone were seven (10.8%). PIRADS 4 or 5 (higher probability for malignancy) lesions were 58 with 56 of them being positive on histopathology giving a positive predictive value of 96.6%. Comparison of the PPVs for cut offs at PI-RADS 3 and 4 was not statistically significant (P = 0.1875).

**Conclusion:** From our experience, prostatic mp-MRI has a high positive predictive value for both PI-RADS 3 and 4 cut-offs in patients with raised PSA levels. No statistically significant difference was found between these two cut-offs.

### P30 Value of MRI in patients with penile cancer: histopathological correlation

#### S. Mukhopadhyay , S. Sen, A. Chandra, D. Lingegowda , P. Ghosh

##### Department of Radiology, Tata Medical Center, Kolkata, India

###### **Correspondence:** S. Mukhopadhyay (dr_sumit007@yahoo.co.in)

**Aim**

1) To evaluate the accuracy of MRI in the preoperative staging of penile cancer

2) To evaluate the most effective MRI protocol and role of IV contrast

3) To determine accuracy of MRI in the non-erect stage of Penis

**Material and Methods:** A total of 49 patients with carcinoma underwent MRI examination.

All the patients had definitive surgery and hitopathological examination

The images were evaluated in consensus by two Radiologists

All the examinations were performed in 3.0Tesla magnet (Siemens, Verio)

High resolution, small FOV images were obtained with Penis taped to the anterior abdominal wall

Intracavernosal agent (Alprostadil) was not used –Non erectile technique

Precontrast T1w, T2w, Fat saturated T2w, post contrast T1w and diffusion weighted sequences were obtained

**Results:** As compared to the histopathology MRI showed overall 89% agreement with sensitivity 84% and specificity 94%. MRI downstaged one case from T2 to T1

As regards to the involvement of subepithelial tissue MRI showed 100% agreement and sensitivity as compared to histopathological examination

MRI also demonstrated 96% and 97% specificity in case of corpora cavernosum and corpora spongiosum involvement respectively

**Conclusion:** Radiological pre-operative stage of carcinoma penis with MRI and can be co-related with histopathological staging with significant accuracy

Significant accuracy can be achieved in MRI even without intracavernosal injection-induced artificial erection

### P31 Prognostic value of ^18^FDG PET/CT in patients listed for liver transplantation for hepatocellular carcinoma

#### Reizine E, Oubaya N, Evangelista E, Luciani A, Mulé S, Rahmouni A, Lerman L, Natella PA, Calderaro J, Laurent A, Duvoux C, Decaens T, Itti E

##### CHU Henri Mondor, Créteil, France

###### **Correspondence:** Itti E (emmanuel.itti@aphp.fr)

**Aim:** Retrospective studies suggest that a positive ^18^FDG PET/CT is associated with an increased risk of tumour recurrence after liver transplantation (LT) for hepatocellular carcinoma (HCC). This study sought to assess its prognostic value in a prospective cohort.

**Methods:** A prospective cohort study was conducted in 371 HCC patients listed for LT to assess independent predictive factors of tumour recurrence after LT. Between 01/2009 and 12/2012, 70 patients underwent whole-body ^18^FDG PET/CT within 3 months of inclusion. Images were interpreted by consensus of 2 observers and ^18^FDG uptake was quantified by calculating tumour-to-normal liver ratio (T/N).

**Results:** Among the 70 patients (87% males, age 58 ± 4 years), PET/CT was positive in 16 patients with a median T/N ratio of 1.30 (range, 1.12-3.08) and negative in 54 with a T/N ratio ≤1. Clinical, and biological features at listing (risk factors, underlying hepatopathy, Child-Pugh, alfa-FP) were similar between PET-positive and PET-negative patients. In the 58 transplanted patients, pathological features on explanted livers (micro-vascular invasion, Edmondson grade, capsule invasion) were not statistically different. Interestingly, PET-positive patients dropped-off more frequently from the waiting list than PET-negative patients, 18.8% vs. 3.8% (P = 0.08) and had slightly lower 2-year overall survival, 61.5% vs. 77.5% (P = 0.23). With a T/N positivity cutoff >1.15, drop-off frequencies were 21.4% vs. 3.6%, respectively (P = 0.05).

**Conclusion:** In this prospective series of very-good prognosis HCC with 75% 2-year overall survival, ^18^FDG PET/CT could predict LT list drop-off rate. Longer follow-up is needed to study its prognostic value on overall survival (NCT01198704).

### P32 Recurrence in breast cancer: local versus distant relapse related to time-interval and breast cancer sub-type

#### Pinto K., Bansal J.

##### Cardiff & Vale University Trust, Cardiff, UK

###### **Correspondence:** Pinto K (kevinpinto5@gmail.com)

**Aim**: To evaluate the patterns of recurrence of breast cancers in relation to subtypes and elapsed time since original cancer. This would potentially guide follow up imaging.

**Methods**: Between June 2006 and December 2016, 202 patients were discussed in breast multi-disciplinary team meeting (MDTM) with computed tomography of chest, abdomen and pelvis (CT CAP). Patients were grouped based on primary breast cancer staging, local recurrence or distant recurrence from a previous breast cancer. Time interval since the primary cancer was stratified in three time frames; 5 or less years, 6-10 years and more than 10 years (≤5yrs;6-10 yrs;>10yrs).

**Results**: 50/202 (24.7%) patients had recurrent cancers. From 50 patients, 24 (48%) had local recurrence only, whereas 26 (52%) had distant relapse. 10/26 had local and distant relapse. Within the local recurrence category, majority relapsed less than 5 years from the primary diagnosis (13 patients), followed by more than 10 years (7 patients) with only 4 patients in the 6-10 years’ time interval. In patients with distant relapse, ≤5yrs; 6-10 yrs;>10yrs was 16:6:4 respectively. Mean time till recurrence was 6.85 years for ER positive cancer versus 3.1 years for triple negative cancer, which also had the highest percent of recurrence (37.8%).

**Conclusion**: Most recurrent cancers (local and distant) present within 5 years, but lesser numbers continue to present in later years. Triple negative cancer present the earliest with highest percent of recurrence compared to other subtypes.

### P33 Diagnostic value of F18-FDG-PET/CT in CUP syndrome: a single-centre retrospective analysis in a private practice

#### I. Sacarea, J. Müller-Hübenthal, A. Zöllich

##### Zentrum für Nuklearmedizin und PET/CT Bremen, Praxis im Köln Triangle, Praxis im Köln Triangle, Cologne, Germany

###### **Correspondence:** I. Sacarea (ilinca.sacarea@gmx.de)

**Aim**: Metastatic malignancies without a primary tumour are known as cancer of unknown primary or CUP Syndrome. For this study, we distinguish between cervical tumours (mostly squamous) and extracervical tumours (usually adenocarcinomas). The life expectancy of these patients may increase by proper identification of the original tumour and consequent adapted therapy. The aim of this analysis is to show the value of F18-FDG-PET/CT in the diagnosis of primary tumours in comparison to conventional diagnostics based on the database of Praxis im Köln Triangle, Germany.

**Methods:** Between 2008 and 2017 out of a total cohort of 4464 cases we performed 227 F18-FDG PET/CTs for 194 patients (90 male/104 female, age 23-93y.) with CUP Syndrome. So far 94 cases (42%) were tracked (medical letters, histology, therapeutic approach). Conventional diagnostics (CT, MRI, scintigraphy, panendoscopy) was primarily performed in almost all patients.

**Results**: We could identify a possible primary in 147 cases (65%), 102 cases additional to conventional diagnostics. For cervical tumours, the primary was most commonly located in the Nasopharynx. Adenocarcinomas could be mostly identified in lungs and GI Tract. Previously unknown tumour metastases have been identified in 42% of cases. The examination-related sensitivity for the detection of primary tumours was 92% for cervical tumours and the specificity was 87%. For extracervical tumours these values were 93%, respectively 90%.

**Conclusion**: The F18-FDG PET/CT can identify additional primary tumours and metastases in 52% of cases when compared to conventional diagnostics and facilitate possible life-prolonging therapy.

